# Supplementary material for: Association between surgeon grade and implant survival following hip and knee replacement: a systematic review and meta-analysis
Source: BMJ Open. 2021 Nov 10;11(11):e047882. doi: 10.1136/bmjopen-2020-047882 (PMC8587578; doi:10.1136/bmjopen-2020-047882)
Supplement: Supplementary data [file bmjopen-2020-047882supp001.pdf]

## Online Supplementary Material

### Contents:

### Supplementary Methods

- Methods (page 2-3): Search strategy using Ovid (Medline + Embase)
- Methods (page 4): Eligibility criteria
- Methods (page 5): Reasons for Exclusion

### Supplementary Table

- Table 3 (page 6): Risk of Bias (ROBINS-I) assessment of methodological quality



Fowler TJ, *et al. BMJ Open* 2021; 11:e047882. doi: 10.1136/bmjopen-2020-047882

**Methods: Search strategy using Ovid (Medline + Embase). Performed by TF & AS.**

**Hip Search**

**Hip replacement**

Hip Prosthesis/ OR Arthroplasty, Replacement, Hip/ OR

(hip adj2 arthroplast$.mp) OR (hip adj2 replacement?.mp) OR (hip adj2 prosthes$.mp) OR

THA.mp OR THR.mp OR (TJR$.mp AND hip$.mp)

**AND**

**Training**

exp Education, Medical/ OR exp Inservice Training/ OR Clinical Competence/ OR

training.mp OR trainee.mp OR

experience.mp OR

junior.mp OR

senior$.mp OR

(surgeon adj2 grade).mp OR

consultant.mp OR attending?.mp OR registrar.mp OR SpR.mp OR StR.mp OR ST?.mp OR residen$.mp OR fellow$.mp OR intern.mp OR

(house adj2 officer).mp OR (foundation adj2 doctor).mp

**AND**

**Survival**

exp Prosthesis Failure/ OR exp Survival Analysis/ OR Reoperation/ OR

cox.mp OR proportional?hazard?.mp OR proportional hazard?.mp OR

cumulative?incidence?function.mp OR cumulative incidence function.mp OR CIF.mp OR

failure.mp OR

survival.mp OR survivor?ship.mp OR

revision?.mp OR

re?operation.mp OR re operation.mp OR

Kaplan?meier.mp OR Kaplan meier.mp OR KM.mp OR

product?limit?method.mp OR product limit method.mp

**AND**

**Case-series**

exp Cohort Studies/ OR Controlled Clinical Trials

follow?up.mp OR follow up.mp OR series.mp OR cohort.mp OR observational.mp OR longitudinal.mp OR prospective.mp OR retrospective.mp OR registry.mp OR registries.mp



Fowler TJ, *et al. BMJ Open* 2021; 11:e047882. doi: 10.1136/bmjopen-2020-047882

**Knee search**

**Knee replacement**

Knee Prosthesis/ OR Arthroplasty, Replacement, Knee/ OR

(knee adj2 arthroplast$.mp) OR (knee adj2 replacement?.mp) OR (knee adj2 prosthes$.mp) OR

TKA.mp OR TKR.mp OR (TJR$.mp AND knee$.mp) OR

UKA.mp OR UKR.mp

**AND**

**Training**

exp Education, Medical/ OR exp Inservice Training/ OR Clinical Competence/ OR

training.mp OR trainee.mp OR

experience.mp OR

junior.mp OR

senior$.mp OR

(surgeon adj2 grade).mp OR

consultant.mp OR attending?.mp OR registrar.mp OR SpR.mp OR StR.mp OR ST?.mp OR residen$.mp OR fellow$.mp OR intern.mp OR

(house adj2 officer).mp OR (foundation adj2 doctor).mp

**AND**

**Survival**

exp Prosthesis Failure/ OR exp Survival Analysis/ OR Reoperation/ OR

cox.mp OR proportional?hazard?.mp OR proportional hazard?.mp OR

cumulative?incidence?function.mp OR cumulative incidence function.mp OR CIF.mp OR

failure.mp OR

survival.mp OR survivor?ship.mp OR

revision?.mp OR

re?operation.mp OR re operation.mp OR

Kaplan?meier.mp OR Kaplan meier.mp OR KM.mp OR

product?limit?method.mp OR product limit method.mp

**AND**

**Case-series**

exp Cohort Studies/ OR Controlled Clinical Trials

follow?up.mp OR follow up.mp OR series.mp OR cohort.mp OR observational.mp OR longitudinal.mp OR prospective.mp OR retrospective.mp OR registry.mp OR registries.mp

## Eligibility criteria

### Inclusion criteria:

- Study of predominantly adult patients (≥ 18 years old) undergoing primary hip or knee replacement (including THR, TKR, UKR and hip resurfacing), predominantly for the treatment of osteoarthritis.
- Included articles needed to report the primary and/or secondary outcome measure for two different groups of surgeons defined according to their grade (e.g. trainee vs. consultant). Additional terms used to describe this variable were deemed eligible during screening:
    - **Trainee:** registrar; resident; junior/young surgeon; fellow.
    - **Consultant:** attending; senior surgeon; trainer.
- Minimum follow-up of five years with clearly defined length of follow up.

### Exclusion criteria:

- Index operation performed prior to 1990.
- Follow-up not clearly defined.
- Irrelevant study design, or outcomes (therefore not meeting inclusion criteria above).

Specific examples for exclusion (documented in online supplementary materials page 5 and figure 1):

- Principally a study of surgeon/hospital volume
- Principally a study of implant positioning
- No revision rates/survival analysis reported according to surgeon grade
- No reporting of outcomes according to surgeon grade
- Insufficient reporting of follow-up
- Study of operations performed prior to 1990
- Hip fracture cohort
- Single surgeon series
- Irrelevant systematic review
- Study of cost-analysis



Fowler TJ, *et al. BMJ Open* 2021; 11:e047882. doi: 10.1136/bmjopen-2020-047882

| **Reasons for Exclusion – Hip Papers** | |
|---|---|
| **First author/Year of study** | **Reason for Exclusion** |
| De Vries, 2011 | Principally a study of surgeon/hospital volume |
| Fender, 2003 | Principally a study of surgeon/hospital volume |
| Hooper, 2009 | Principally a study of surgeon/hospital volume |
| Johnsson, 1994 | Principally a study of surgeon/hospital volume |
| Namba, 2012 | Principally a study of surgeon/hospital volume |
| Ravi, 2014 | Principally a study of surgeon/hospital volume |
| Canadian Arthroplasty Soc., | Principally a study of surgeon/hospital volume |
| MacBride, 2010 | Principally a study of surgeon/hospital volume |
| Enocson, 2009 | No revision rates/survival analysis reported according to surgeon grade |
| Field, 2006 | No revision rates/survival analysis reported according to surgeon grade |
| Leguerrand, 2018 | No revision rates/survival analysis reported according to surgeon grade |
| Moran, 2004 | No revision rates/survival analysis reported according to surgeon grade |
| Smith, 2018 | No revision rates/survival analysis reported according to surgeon grade |
| Wilson, 2016 | No revision rates/survival analysis reported according to surgeon grade |
| Wroblewski, 1998 | No revision rates/survival analysis reported according to surgeon grade |
| Schoenfeld, 2013 | No revision rates/survival analysis reported according to surgeon grade |
| Inglis, 2013 | Insufficient reporting of follow-up |
| Marston, 1996 | Study of operations performed prior to 1990 |
| Khatod, 2014 | No reporting of outcomes according to surgeon grade |
| Whitehouse, 2014 | No reporting of outcomes according to surgeon grade |
| Williams, 2002 | No reporting of outcomes according to surgeon grade |
| Zwartele, 2005 | No reporting of outcomes according to surgeon grade |
| Kim, 2017 | Principally a study of implant positioning |
| MacDonald, 2020 | Hip fracture cohort |
| DeAngelis, 2020 | Hip fracture cohort |
| N.B. Multiple reasons for some papers | |

| **Reasons for Exclusion – Knee Papers** | |
|---|---|
| **First author/Year of study** | **Reason for Exclusion** |
| Bini, 2013 | Principally a study of surgeon/hospital volume |
| Namba, 2012 | Principally a study of surgeon/hospital volume |
| Zambianchi, 2014 | Principally a study of surgeon/hospital volume |
| Rissolio, 2021 | Principally a study of surgeon/hospital volume |
| Liddle, 2014 | No revision rates/survival analysis reported according to surgeon grade |
| Beattie, 2016 | No revision rates/survival analysis reported according to surgeon grade |
| Haughom, 2014 | No revision rates/survival analysis reported according to surgeon grade |
| Khakha, 2015 | No revision rates/survival analysis reported according to surgeon grade |
| Schoenfeld, 2013 | No revision rates/survival analysis reported according to surgeon grade |
| Windisch, 2017 | No revision rates/survival analysis reported according to surgeon grade |
| Wilson, 2016 | No revision rates/survival analysis reported according to surgeon grade |
| Woolson, 2007 | No revision rates/survival analysis reported according to surgeon grade |
| Atrey, 2014 | No reporting of outcomes according to surgeon grade |
| Back, 2000 | No reporting of outcomes according to surgeon grade |
| Singh, 2021 | No reporting of outcomes according to surgeon grade |
| Gaillard, 2016 | Principally a study of implant positioning |
| Mahaluxmivala, 2001 | Principally a study of implant positioning |
| Storey, 2018 | Insufficient reporting of follow-up |
| Theelen, 2018 | Insufficient reporting of follow-up |
| Jasper, 2016 | Irrelevant systematic review |
| Lacko, 2018 | Single surgeon series |
| Matas-Diez, 2018 | Principally a study of learning curve |
| Lavernia, 2000 | Study of cost-analysis |
| N.B. Multiple reasons for some papers | |

**Supplementary Table 1: Risk of Bias (ROBINS-I) assessment**

| **ROBINS-I** | **Alvand, 2021** | **Bottomley, 2016** | **Faulkner, 2017** | **Hernigou, 2009** | **Hasegawa, 2015** | **Jain, 2018** | **Muller, 2013** | **Palan, 2009** | **Reidy, 2016** |
|---|---|---|---|---|---|---|---|---|---|
| Bias due to confounding | ⊕⊕ | ⊕⊕ | ⊕ | ⊕⊕ | ⊕⊕ | ⊕ | ⊕⊕ | ⊕ | ⊕ |
| Bias in selection of patients | ⊕ | ⊕ | ⊕ | ⊕ | ⊕ | ⊕ | ⊕ | ⊕ | ⊕ |
| Bias in classification of interventions | ⊖ | ⊖ | ⊕ | ⊕ | ⊕⊕ | ⊕ | ⊕⊕ | ⊕ | ⊕ |
| Bias due to deviations from interventions | ⊕ | ⊕ | ⊕ | ⊕⊕ | ⊕⊕ | ⊕ | ⊕⊕ | ⊕ | ⊕ |
| Bias due to missing data | ⊖ | ⊖ | ⊕ | ⊕⊕ | ⊕⊕ | ⊕ | ⊕ | ⊖ | ⊕ |
| Bias in measurement of outcome | ⊖ | ⊖ | ⊕ | ⊖ | ⊕ | ⊖ | ⊖ | ⊖ | ⊕ |
| Bias in selection of the reported result | ⊖ | ⊖ | ⊖ | ⊖ | ⊕ | ⊖ | ⊕ | ⊖ | ⊖ |
| Overall risk of Bias | ⊕ | ⊕ | ⊕ | ⊕⊕ | ⊕⊕ | ⊕ | ⊕⊕ | ⊕ | ⊕ |
| **Key:** ⊖ = low risk of bias; ⊕ = moderate risk of bias; ⊕⊕ = serious risk of bias; ⊕⊕⊕ = critical risk of bias | | | | | | | | | |
