# Supplementary material for: Association between surgeon grade and implant survival following hip and knee replacement: a systematic review and meta-analysis
Source: BMJ Open. 2021 Nov 10;11(11):e047882. doi: 10.1136/bmjopen-2020-047882 (PMC8587578; doi:10.1136/bmjopen-2020-047882)
Supplement: Author's manuscript [file bmjopen-2020-047882.draft_revisions.pdf]

BMJ Open is committed to open peer review. As part of this commitment we make the peer review history of every article we publish publicly available.

When an article is published we post the peer reviewers' comments and the authors' responses online. We also post the versions of the paper that were used during peer review. These are the versions that the peer review comments apply to.

The versions of the paper that follow are the versions that were submitted during the peer review process. They are not the versions of record or the final published versions. They should not be cited or distributed as the published version of this manuscript.

BMJ Open is an open access journal and the full, final, typeset and author-corrected version of record of the manuscript is available on our site with no access controls, subscription charges or pay-per-view fees (http://bmjopen.bmj.com).

If you have any questions on BMJ Open's open peer review process please email info.bmjopen@bmj.com

BMJ Open

The association between surgeon training status and implant survival following hip and knee replacement: a systematic review and meta-analysis

Journal:	*BMJ Open*
Manuscript ID	bmjopen-2020-047882
Article Type:	Original research
Date Submitted by the Author:	10-Dec-2020
Complete List of Authors:	Fowler, Timothy; University of Bristol Medical School, Musculoskeletal Research Unit<br>Aquilina, Alex; Bristol Medical School, Musculoskeletal Research Unit<br>Blom, AW; University of Bristol Medical School, Musculoskeletal Research Unit; National Institute for Health Research Bristol Biomedical Research Centre, University Hospitals Bristol NHS Foundation Trust, University of Bristol<br>Sayers, Adrian; University of Bristol Medical School, Musculoskeletal Research Unit<br>Whitehouse, Michael; University of Bristol Medical School, Musculoskeletal Research Unit; National Institute for Health Research Bristol Biomedical Research Centre, University Hospitals Bristol NHS Foundation Trust, University of Bristol
Keywords:	Hip < ORTHOPAEDIC & TRAUMA SURGERY, Knee < ORTHOPAEDIC & TRAUMA SURGERY, Orthopaedic & trauma surgery < SURGERY, MEDICAL EDUCATION & TRAINING, Health policy < HEALTH SERVICES ADMINISTRATION & MANAGEMENT

SCHOLARONE™
Manuscripts

I, the Submitting Author has the right to grant and does grant on behalf of all authors of the Work (as defined in the below author licence), an exclusive licence and/or a non-exclusive licence for contributions from authors who are: i) UK Crown employees; ii) where BMJ has agreed a CC-BY licence shall apply, and/or iii) in accordance with the terms applicable for US Federal Government officers or employees acting as part of their official duties; on a worldwide, perpetual, irrevocable, royalty-free basis to BMJ Publishing Group Ltd ("BMJ") its licensees and where the relevant Journal is co-owned by BMJ to the co-owners of the Journal, to publish the Work in this journal and any other BMJ products and to exploit all rights, as set out in our licence.

The Submitting Author accepts and understands that any supply made under these terms is made by BMJ to the Submitting Author unless you are acting as an employee on behalf of your employer or a postgraduate student of an affiliated institution which is paying any applicable article publishing charge ("APC") for Open Access articles. Where the Submitting Author wishes to make the Work available on an Open Access basis (and intends to pay the relevant APC), the terms of reuse of such Open Access shall be governed by a Creative Commons licence – details of these licences and which Creative Commons licence will apply to this Work are set out in our licence referred to above.

Other than as permitted in any relevant BMJ Author's Self Archiving Policies, I confirm this Work has not been accepted for publication elsewhere, is not being considered for publication elsewhere and does not duplicate material already published. I confirm all authors consent to publication of this Work and authorise the granting of this licence.

For peer review only

The association between surgeon training status and implant survival following hip and knee replacement: a systematic review and meta-analysis

Authors

Timothy J. Fowler, MRCS[1]

Alex L. Aquilina, MRCS[1]

Ashley W. Blom, PhD[1,2]

*Adrian Sayers, MSc[1]

*Michael R. Whitehouse, PhD[1,2]

**Joint Senior Authors*

Author Affiliations

[1]Musculoskeletal Research Unit, Translational Health Sciences, Bristol Medical School, Southmead Hospital, Bristol, UK

[2]National Institute for Health Research Bristol Biomedical Research Centre, University Hospitals Bristol NHS Foundation Trust, University of Bristol, Bristol, UK

Corresponding Author

Mr Timothy J Fowler, BMBCh (Oxon) MA MRCS

Address: Musculoskeletal Research Unit, Translational Health Sciences, Bristol Medical School, Southmead Hospital, Bristol, UK, BS10 5NB

Email: t.j.fowler@bristol.ac.uk; *Phone:* +44 (0)117 414 7865

Word count

2948

For peer review only

Abstract

Objective: To investigate the association between the training status of the surgeon (i.e. trainee vs. consultant) and implant survival following primary hip and knee replacement.

Design: A systematic review and meta-analysis of observational studies.

Data sources: MEDLINE® and Embase® from inception until December 10th, 2020.

Setting: Units performing primary hip and/or knee replacements since 1990.

Participants: Adult patients undergoing either a primary hip or knee replacement, predominantly for osteoarthritis.

Intervention: Whether the surgeon recorded as performing the procedure was a trainee or not.

Primary and secondary outcome measures: The primary outcome was net implant survival (i.e. absence of revision surgery), reported as a Kaplan-Meir survival estimate. The secondary outcome was crude revision rate. Both outcomes were reported according to the training status of the surgeon.

Results: Eight cohort studies capturing 4066 total hip replacements (THRs), 936 total knee replacements (TKRs), and 1084 unicompartmental knee replacements (UKRs) were included. The pooled net implant survival estimates for THRs at five years follow up were 97.9% (95% CI 96.6 to 99.2) for trainees and 98.1% (95% CI 97.1 to 99.2) for consultants. For TKRs, the net implant survival estimates at ten years follow up were 96.2% (95% CI 94.0 to 98.4) for trainees and 95.1% (95% CI 93.0 to 97.2) for consultants.

Conclusions: There is no strong evidence in the existing literature that trainee surgeons have worse outcomes compared to their consultant colleagues, in terms of the net survival or crude revision rate of hip and knee replacements at five to ten years follow up. These findings are applicable to countries with established orthopaedic training programmes.

For peer review only

For peer review only - http://bmjopen.bmj.com/site/about/guidelines.xhtml

Article Summary

Strengths and limitations of this study

- To our knowledge, this is the first meta-analysis of the association between surgeon training status and implant survival following hip and knee replacement.
- We performed a comprehensive systematic review according to current best practice guidelines.
- The findings of this review are limited by the strength of the existing published data from a relatively small number of predominantly retrospective observational studies.

For peer review only

Introduction

Hip and knee replacements are effective surgical interventions for the treatment of end stage degenerative conditions of the hip and knee.[1 2] More than 200,000 are performed per year in the United Kingdom alone.[3] These procedures are performed by surgeons at various stages in their training, with varying levels of senior supervision. Contemporary training practices must ensure a balance between protecting development opportunities for the next generation of surgeons, while limiting the exposure of patients to unnecessary risk during the training process.

Implant survival, which is determined by the absence of revision surgery, is an important and commonly used measure of surgical performance.[4 5] Net survival estimates are calculated using statistical methods of survival analysis (e.g. Kaplan-Meier analysis), which look at time to a defined failure 'event' (e.g. revision) and account for censored data that arise due to incomplete follow up, or death.[6] Another commonly reported metric is crude revision rate, which is defined as the observed number of failure events in a specified period of time.

The survival of hip and knee replacements according to the training status of the surgeon is poorly understood. Higher rates of complications and longer operative times have been identified in orthopaedic procedures performed by trainees.[7 8] Radiographic studies have indicated that trainees achieve different implant alignment to their senior colleagues, in terms of acetabular anteversion,[9] hip centre of rotation,[10] and various measures of knee replacement component positioning.[11] However, the causative impact of these findings on implant survival has not been established. It has been suggested that when trainees are appropriately supervised, they can obtain comparable functional outcomes and implant survivorship to their consultant colleagues when performing total hip replacement (THR),[12-14] total knee replacement (TKR)[15] and unicompartmental knee replacement (UKR).[16]

The aim of this study was to conduct a systematic review and meta-analysis using the existing literature on the association between the training status of the surgeon (trainee vs. consultant) and implant survival outcomes in hip and knee replacement surgery.

For peer review only - http://bmjopen.bmj.com/site/about/guidelines.xhtml

Methods

Data sources and search strategy

This review was conducted using methods described in the Cochrane Handbook for Systematic Reviews of Interventions, with reporting in accordance with the Meta-analyses Of Observational Studies in Epidemiology (MOOSE) checklist.[17 18] The study was registered with the PROSPERO database at inception (CRD42019150494).

We searched for cohort studies reporting implant survival estimates and/or revision rates of hip or knee replacements, according to the training status of the surgeon. Separate searches were performed for hips and knees. We conducted searches of MEDLINE® and Embase™ from inception until December 10th, 2020. Searches used keywords and MeSH (Medical Subject Headings) terms relating to hip and knee replacement, implant survival, revision surgery and surgeon training status (see online supplementary methods). There were no language restrictions. Titles and abstracts of potentially relevant non-English language citations were translated. We manually screened the bibliographies of full text articles and used Web of Science™ citation tracking to identify additional relevant studies.

Eligibility criteria

We included studies if they involved predominantly unselected adult patients (≥ 18 years old) undergoing primary hip or knee replacement (including THR, TKR, UKR and hip resurfacing), predominantly for the treatment of osteoarthritis. Included articles needed to report the primary and/or secondary outcome measure for two different groups of surgeons according to their training status (e.g. trainee vs. consultant). We defined a minimum follow up of five years and articles that did not clearly define the length of follow up were excluded. For example, we excluded studies reporting the revision rate 'per 100 component years', as these did not explicitly define the length of follow up. We excluded studies in which the index operation was performed prior to 1990; thereby, including studies that are representative of contemporary training practices, but also allowing for inclusion of studies reporting up to 30 years of follow up.

For peer review only - http://bmjopen.bmj.com/site/about/guidelines.xhtml

Primary exposure

The primary exposure was whether the surgeon recorded as performing the procedure was a trainee or not. The 'training status' of the surgeon is a measure of the designated level of surgical experience and seniority, which we considered to be a binary variable: either 'trainee', or 'consultant'. Consultant surgeons have completed their formal training in orthopaedic surgery and have been appointed to a senior position in which they can practice independently and supervise trainee surgeons. The term 'consultant' is used synonymously with 'attending surgeon' in many healthcare settings including the United States. Additional terms used to describe this variable were deemed eligible during screening (e.g. Trainee: registrar; resident; junior/young surgeon; fellow. Consultant: attending; senior surgeon; trainer).

Outcome measures

The primary outcome was net implant survival, reported as a Kaplan-Meir survival estimate. The secondary outcome measure was crude revision rate, which was defined as the observed number of revisions in a specified period of time.

Screening and data extraction

Two authors (TJF and ALA) independently screened all titles and abstracts of journal articles using Rayyan (Rayyan QCRI, Doha). Cases of disagreement were resolved through re-review and consensus. Full texts of potentially relevant studies were reviewed in detail and disagreements on final inclusion were resolved through discussion with a senior author (MRW).

Data were extracted in duplicate using a standardised proforma. We recorded data on the following: healthcare setting, study period, implant type, age, sex, indication, level of supervision, crude revision rate, and net implant survival estimates (including confidence intervals [CI]). Life tables were reviewed, and estimates were extracted for all available five-year intervals of follow up. Discrepancies in data collection were resolved through re-review and consensus. Where survival

For peer review only - http://bmjopen.bmj.com/site/about/guidelines.xhtml

For peer review only

estimates, CIs and revision rates were incompletely reported, we contacted corresponding authors to request missing data.

Quality assessment

The risk of bias was assessed using the Cochrane ROBINS-I tool for the risk of bias in non-randomised cohort studies.[19] We assessed the quality of evidence for each outcome using the Grading of Recommendations Assessment, Development and Evaluation (GRADE) approach, which considers the imprecision, inconsistency, indirectness, and risk of bias in a body of evidence.[20]

Statistical analysis

Statistical analysis was performed using Stata (Version SE 15.1; StataCorp, Texas). For the primary outcome measure of net implant survival, we performed separate meta-analyses for each implant type, by training status and length of follow up. We pooled survival estimates, assuming that survivorship approximated risk, with fixed effects meta-analysis weighting each study on the overall pooled estimate according to its standard error, which was calculated from published CIs; a method described by Evans *et al*.[4 5] The effect size (survival) for trainees and consultants, was compared using a Wald test. For the secondary outcome measure, we derived and meta-analysed the relative risk (RR) of revision for each implant type by training status and length of follow up. We used a fixed effects model using the Mantel-Haenszel method.[21] Heterogeneity was assessed with chi-squared tests, with I^2 used to quantify inconsistency.[22] Publication bias was assessed by inspecting funnel plot symmetry.[23]

Patient and public involvement

There was no direct patient or public involvement in the design or conduct of this review.

For peer review only - http://bmjopen.bmj.com/site/about/guidelines.xhtml

Results

Separate searches for hip and knee replacements identified 1106 and 589 articles, respectively. After removal of duplicates and abstract screening, 29 hip papers and 24 knee papers remained. Through review of full text articles, we identified five hip and three knee studies eligible for inclusion. We identified no randomised controlled trials (RCTs) on this subject. This process of review is summarised as a flow diagram in figure 1 and the characteristics of included studies are summarised in table 1. Five studies were conducted in the UK, with the remaining three studies originating from France, Switzerland, and Japan.

Quality assessment

Online supplementary table 3 provides a summary of the ROBINS-I assessment, which indicates a moderate to severe risk of bias in all studies. Funnel plot asymmetry and statistical tests for funnel plot asymmetry as a means of assessing publication bias were not applicable due to the small number of studies.[24] The GRADE assessment for the quality of evidence for each outcome indicates a low, or very low quality of evidence for all outcomes (table 2).

Hip replacement

The five included hip studies represent 1464 THRs performed by trainees and 2602 THRs performed by consultants, with follow up ranging from five to ten years. Four studies were retrospective cohort studies;[12 13 25 26] one was a non-randomised prospective cohort study.[27] No articles on hip resurfacing met the inclusion criteria. One author provided additional unpublished data in the form of net survival estimates.[26] Reidy *et al* reported survival estimates, but no CIs.[13] Net survival estimates and corresponding CIs were thus extracted from three studies at five years and one study at ten years. Crude revision rates were reported in three studies at five years and two studies at ten years.

For peer review only - http://bmjopen.bmj.com/site/about/guidelines.xhtml

For peer review only

Primary outcome: Net implant survival (THR)

Meta-analysis showed net survivorship of 97.9% (95% CI 96.6 to 99.2) at five years for THRs performed by trainees, compared to 98.1% (95% CI 97.1 to 99.2) for THRs performed by consultants (figure 2). There was no strong evidence of an association between training status and net implant survival at this interval of follow up (Wald test: 0.28; 95% CI -1.37 to 1.93; p=0.74).

Meta-analysis was not possible for the ten-year data given the availability of only one study for this timepoint. In a cohort of 1082 reverse hybrid THRs, Jain *et al* demonstrated overall 97.2% implant survival at ten years. Additional data provided by the author indicate that they found no evidence of a difference in implant survival according to the training status of the surgeon (Trainee: 98.1%; 95% CI 95.9 to 99.1; Consultant: 96.7%; 95% CI 94.7 to 97.9; log rank: p=0.36).[26]

Secondary outcome: Crude revision rate (THR)

Meta-analysis showed no strong evidence of an association between training status and the crude revision rate at five, or ten years. The RR of revision at five and ten years was 0.88 (95% CI 0.46 to 1.70; Z=0.37; p=0.71) and 0.68 (95% CI 0.37 to 1.26; Z=1.22; p=0.22), respectively (figure 3).

Knee replacement

The three knee studies represent 1059 knee replacements performed by trainees and 961 performed by consultants, with follow up ranging from ten to fifteen years. All three were retrospective cohort studies.[15 16 28] Only one study reported on UKRs,[16] thus further quantitative analysis was limited to the two TKR papers.[15 28] Faulkner *et al* provided additional unpublished survival data from which we calculated corresponding CIs for their published survival estimates.[15] Net survival estimates and CIs were thus extracted from both TKR studies at ten years. Crude revision rates were only available from one study at each five-year interval of follow up.

For peer review only - http://bmjopen.bmj.com/site/about/guidelines.xhtml

Primary outcome: Net implant survival (TKR)

Meta-analysis showed net survivorship of 96.2% (95% CI 94.0 to 98.4) at ten years for TKRs performed by trainees, compared to 95.1% (95% CI 93.0 to 97.2) for TKRs performed by consultants (Fig. 4). There was no strong evidence of an association between training status and net implant survival at this interval of follow up (Wald test: 1.08; 95% CI -1.95 to 4.10; p=0.49).

Secondary outcome: Crude revision rate (TKR)

Two studies reported crude revision rates according to surgeon training status; however, with data from only one study available at each interval of follow up, meta-analysis was not feasible. Instead, we provide a narrative summary. Faulkner *et al* provided additional unpublished data, which indicated crude revision rates at five years for trainees and consultants of 2.1% and 4.4%, respectively.[15] This rises to 3.4% (trainees) and 5.8% (consultants) at ten years. These data represent a RR of revision of 0.49 (95% CI 0.19 to 1.28) at five years and 0.60 (95% CI 0.28 to 1.31) at ten years. Hernigou published crude revision rates at 15 years of 2.7% for junior surgeons and 4% for senior surgeons, which represents a RR of revision of 0.68 (95% CI 0.17 to 2.64).[28]

Unicompartmental knee replacement (UKR)

A single study reported survivorship outcomes for UKRs according to training status.[16] Bottomley *et al* conducted a retrospective cohort study of 1084 consecutive UKRs. They demonstrated that consultants and trainees had cumulative 9-year survival estimates of 93.9% and 93%, respectively. They found no strong evidence of a difference in implant survival between the groups (log rank: p=0.30).[16]

For peer review only - http://bmjopen.bmj.com/site/about/guidelines.xhtml

Discussion

The results of this study suggest that, in the context of contemporary practice, trainees do not achieve worse hip and knee replacement survival outcomes compared to their consultant colleagues at five to ten years follow up. We found no strong evidence of an association between the training status of the surgeon and the net survival of THRs at five years (trainees: 97.9% vs consultants: 98.1%). There was no association between training status and the crude revision rate of THRs at either five, or ten years follow up. Furthermore, we found no strong evidence of an association between training status and the net survival of TKRs at ten years (trainees: 96.2% vs consultants: 95.1%).

Strengths and limitations

This review has a number of strengths. We conducted a comprehensive systematic review with an exhaustive search according to current best practice guidelines and published the protocol for the methodology at inception. However, the data captured by this review have several limitations, which we have attempted to address through quality assessment. The GRADE assessment, which incorporates our risk of bias analysis, indicates the quality of evidence for each outcome to be low/very low, which is consistent with the predominantly retrospective design of included studies. Thus, the conclusions of this review are limited by the strength of the existing published data from a relatively small number of observational studies.

Meta-analysis of the primary outcome measure was only possible at five and ten years for THRs and ten years for TKRs, which limits the generalisability of our findings to these short and medium-term intervals of follow up. The included studies originated from the UK, France, Switzerland, and Japan, which limits the generalisability of the findings to countries with established orthopaedic training programmes.

Implant survival is a key determinant of good outcome in joint replacement surgery and is the sole variable considered in the current benchmarking strategies for the assessment of implant components. However, this review did not consider other factors that may be important when evaluating surgical

For peer review only - http://bmjopen.bmj.com/site/about/guidelines.xhtml

outcomes, such as patient reported outcome measures, or complications other than failure. Published literature did not consistently report age, sex, comorbidities, implant details, or the level of senior supervision; making it very difficult to adjust for these variables. It is reasonable to suggest that the predominantly superior survival outcomes observed in the trainee cohorts are a product of patient selection and close senior supervision, with good trainers selecting appropriately complex cases for their trainees.

Comparison with other studies

A single study was excluded because the THRs under follow up were performed prior to 1990;[29] thus not considered representative of contemporary training practices. The authors of this ten-year study of 413 THRs reported a significantly higher rate of revision for trainees, with 15 of 16 revised hips performed by trainees. Inclusion of this study in our meta-analysis of ten-year THR crude revision rates increases the RR of revision to 1.12 (95% CI 0.66 to 1.92; p=0.67), in favour of THRs performed by consultants. One explanation for this is that the model of training in the UK at the time differed, with trainees more often operating without appropriate senior supervision.

Our findings are consistent with those of the New Zealand Joint Registry.[30 31] In a cohort of 35 415 THRs, of which 4049 were performed by trainees, the authors reported no significant difference in the revision rate between surgeon groups.[31] In a further cohort of 79 671 TKRs and 8854 UKRs, of which approximately 10% were performed by trainees, they reported no significant difference in the revision rates of knee replacements performed by trainees and consultants.[30] These studies were not included in this meta-analysis because the authors did not report net survival estimates and revision rates were reported ‘per 100 component years’, rather than for clearly defined periods of follow up, which cannot be calculated from the data presented.

Implications

There is a delicate balance between ensuring optimal outcomes for patients and the necessity to train the next generation of surgeons. Reidy and Faulkner suggest that the availability of surgeon level

For peer review only - http://bmjopen.bmj.com/site/about/guidelines.xhtml

registry data as a means of benchmarking performance, may lead to a desire to avoid perceived poor performance and thus a reluctance among consultants to let trainees operate.[13 15] However, the findings of this review are encouraging and support the notion that in the context of contemporary practice, in countries with established orthopaedic training programmes, trainees can achieve implant survival outcomes equivalent to their consultant colleagues. The senior supervision of trainees was inconsistently reported in the studies included in this review but is likely to play an important role in the successful outcome of trainee performed hip and knee replacements.

An adequately powered non-inferiority RCT with ten years follow up assuming an acceptable revision rate of 5% and a 1% absolute non-inferiority delta ($\alpha = 0.05$; power = 0.80; 1:1 allocation ratio), would require a sample size of 6400 patients.[32] However, factors inherent to the training process, such as variation amongst trainees, the need for case selection and varying levels of supervision based on a trainee's experience, may preclude an inclusive and therefore generalisable RCT.

Conclusions

In conclusion, there is no strong evidence in the existing literature that trainee surgeons have worse outcomes than their consultant surgeon colleagues, in terms of the net survival, or crude revision rate of hip and knee replacements at five to ten years follow up. This may mean that there is no difference, or that appropriate case mix selection and supervision of trainees is currently employed and is safe to continue. Our results are concordant with published registry data,[30 31] and represent the best available evidence.

For peer review only

References

1. Ferguson RJ, Palmer AJ, Taylor A, et al. Hip replacement. *Lancet* 2018;392(10158):1662-71. doi: 10.1016/S0140-6736(18)31777-X
2. Price AJ, Alvand A, Troelsen A, et al. Knee replacement. *Lancet* 2018;392(10158):1672-82. doi: 10.1016/S0140-6736(18)32344-4
3. 17th Annual Report: The National Joint Registry for England, Wales, Northern Ireland and the Isle of Man, 2020.
4. Evans JT, Evans JP, Walker RW, et al. How long does a hip replacement last? A systematic review and meta-analysis of case series and national registry reports with more than 15 years of follow-up. *Lancet* 2019;393(10172):647-54. doi: 10.1016/S0140-6736(18)31665-9
5. Evans JT, Walker RW, Evans JP, et al. How long does a knee replacement last? A systematic review and meta-analysis of case series and national registry reports with more than 15 years of follow-up. *Lancet* 2019;393(10172):655-63. doi: 10.1016/S0140-6736(18)32531-5
6. Sayers A, Evans JT, Whitehouse MR, et al. Are competing risks models appropriate to describe implant failure? *Acta Orthop* 2018;89(3):256-58. doi: 10.1080/17453674.2018.1444876
7. Hedlundh U, Ahnfelt L, Hybbinette CH, et al. Surgical experience related to dislocations after total hip arthroplasty. *J Bone Joint Surg Br* 1996;78(2):206-9.
8. Schoenfeld AJ, Serrano JA, Waterman BR, et al. The impact of resident involvement on post-operative morbidity and mortality following orthopaedic procedures: a study of 43,343 cases. *Arch Orthop Trauma Surg* 2013;133(11):1483-91. doi: 10.1007/s00402-013-1841-3
9. Moran M, Yap SL, Walmsley P, et al. Clinical and radiologic outcome of total hip arthroplasty performed by trainee compared with consultant orthopedic surgeons. *J Arthroplasty* 2004;19(7):853-7. doi: 10.1016/j.arth.2004.06.026
10. Kim SC, Lim YW, Kwon SY, et al. Level of surgical experience is associated with change in hip center of rotation following cementless total hip arthroplasty: A radiographic assessment. *PLoS One* 2017;12(5):e0178300. doi: 10.1371/journal.pone.0178300
11. Kazarian GS, Lawrie CM, Barrack TN, et al. The Impact of Surgeon Volume and Training Status on Implant Alignment in Total Knee Arthroplasty. *J Bone Joint Surg Am* 2019;101(19):1713-23. doi: 10.2106/JBJS.18.01205
12. Palan J, Gulati A, Andrew JG, et al. The trainer, the trainee and the surgeons' assistant: clinical outcomes following total hip replacement. *J Bone Joint Surg Br* 2009;91(7):928-34. doi: 10.1302/0301-620X.91B7.22021
13. Reidy MJ, Faulkner A, Shitole B, et al. Do trainee surgeons have an adverse effect on the outcome after total hip arthroplasty?: a ten-year review. *Bone Joint J* 2016;98-B(3):301-6. doi: 10.1302/0301-620X.98B3.35997
14. Beattie N, Maempel JF, Roberts S, et al. Surgery performed by supervised registrars does not adversely affect medium-term functional outcomes after total knee replacement. *Ann R Coll Surg Engl* 2018;100(1):57-62. doi: 10.1308/rcsann.2017.0164
15. Faulkner A, Reidy MJ, Davies PSE, et al. Balancing training and outcomes in total knee replacement: A ten-year review. *Surgeon* 2018;16(4):227-31. doi: 10.1016/j.surge.2017.10.003
16. Bottomley N, Jones LD, Rout R, et al. A survival analysis of 1084 knees of the Oxford unicompartmental knee arthroplasty: a comparison between consultant and trainee surgeons. *Bone Joint J* 2016;98-B(10 Supple B):22-27. doi: 10.1302/0301-620X.98B10.BJJ-2016-0483.R1
17. Higgins JPT, Green S, Cochrane Collaboration. Cochrane handbook for systematic reviews of interventions. Chichester, England ; Hoboken, NJ: Wiley-Blackwell 2008.
18. Stroup DF, Berlin JA, Morton SC, et al. Meta-analysis of observational studies in epidemiology: a proposal for reporting. Meta-analysis Of Observational Studies in Epidemiology (MOOSE) group. *JAMA* 2000;283(15):2008-12. doi: 10.1001/jama.283.15.2008
19. Sterne JA, Hernan MA, Reeves BC, et al. ROBINS-I: a tool for assessing risk of bias in non-randomised studies of interventions. *BMJ* 2016;355:i4919. doi: 10.1136/bmj.i4919

For peer review only - http://bmjopen.bmj.com/site/about/guidelines.xhtml

20. Atkins D, Best D, Briss PA, et al. Grading quality of evidence and strength of recommendations. *BMJ* 2004;328(7454):1490. doi: 10.1136/bmj.328.7454.1490
21. Mantel N, Haenszel W. Statistical aspects of the analysis of data from retrospective studies of disease. *J Natl Cancer Inst* 1959;22(4):719-48.
22. Higgins JP, Thompson SG. Quantifying heterogeneity in a meta-analysis. *Stat Med* 2002;21(11):1539-58. doi: 10.1002/sim.1186
23. Egger M, Davey Smith G, Schneider M, et al. Bias in meta-analysis detected by a simple, graphical test. *BMJ* 1997;315(7109):629-34. doi: 10.1136/bmj.315.7109.629
24. Sterne JA, Sutton AJ, Ioannidis JP, et al. Recommendations for examining and interpreting funnel plot asymmetry in meta-analyses of randomised controlled trials. *BMJ* 2011;343:d4002. doi: 10.1136/bmj.d4002
25. Muller DA, Zingg PO, Dora C. Anterior minimally invasive approach for total hip replacement: five-year survivorship and learning curve. *Hip Int* 2014;24(3):277-83. doi: 10.5301/hipint.5000108
26. Jain S, Magra M, Dube B, et al. Reverse hybrid total hip arthroplasty. *Bone Joint J* 2018;100-B(8):1010-17. doi: 10.1302/0301-620X.100B8.BJJ-2017-1297.R1
27. Hasegawa Y, Amano T. Surgical skills training for primary total hip arthroplasty. *Nagoya J Med Sci* 2015;77(1-2):51-7.
28. Hernigou P, Manicom O, Flouzat-Lachaniete CH, et al. Fifteen year outcome of the ceraver hermes posterior-stabilized total knee arthroplasty: safety of the procedure with experienced and inexperienced surgeons. *Open Orthop J* 2009;3:36-9. doi: 10.2174/1874325000903010036
29. Marston RA, Cobb AG, Bentley G. Stanmore compared with Charnley total hip replacement. A prospective study of 413 arthroplasties. *J Bone Joint Surg Br* 1996;78(2):178-84.
30. Storey R, Frampton C, Kieser D, et al. Does Orthopaedic Training Compromise the Outcome in Knee Joint Arthroplasty? *J Surg Educ* 2018;75(5):1292-98. doi: 10.1016/j.jsurg.2018.02.011
31. Inglis T, Dalzell K, Hooper G, et al. Does orthopedic training compromise the outcome in total hip arthroplasty? *J Surg Educ* 2013;70(1):76-80. doi: 10.1016/j.jsurg.2012.08.003
32. Sayers A, Crowther MJ, Judge A, et al. Determining the sample size required to establish whether a medical device is non-inferior to an external benchmark. *BMJ Open* 2017;7(8):e015397. doi: 10.1136/bmjopen-2016-015397

For peer review only

For peer review only - http://bmjopen.bmj.com/site/about/guidelines.xhtml

Figure and Title Legends

Fig. 1 – Study flow diagram

Fig. 2 – Meta-analysis of net implant survival of THRs at five years according to the training status of the surgeon

Fig. 3 – Meta-analysis of the relative risk of revision of THRs at five and ten years according to the training status of the surgeon

Fig. 4 – Meta-analysis of net implant survival of TKRs at ten years according to the training status of the surgeon

Table 1 – Characteristics of included studies

Table 2 – GRADE Summary of Findings Table

Supplementary Table 3 – Risk of bias (ROBINS-I) assessment of methodological quality

For peer review only

For peer review only - http://bmjopen.bmj.com/site/about/guidelines.xhtml

Contributors

TF, AB, AS, and MW conceived and designed the study; TF and AA independently screened the articles and performed data extraction in duplicate; TF and AS were responsible for data analysis; all authors were responsible for interpreting the data; TF drafted the manuscript; AB, AA, AS, and MW revised the article critically for important intellectual content; all authors reviewed the final version of the manuscript and gave approval for submission for publication. The corresponding author attests that all listed authors meet authorship criteria and that no others meeting the criteria have been omitted.

Funding

This study was also supported by the National Institute for Health Research (NIHR) Biomedical Research Centre at the University Hospitals Bristol NHS Foundation Trust and the University of Bristol. TF was supported by a NIHR Academic Clinical Fellowship. AS was supported by an MRC strategic skills fellowship (MR/L01226X/1). The views expressed in this publication are those of the authors and not necessarily those of the NHS, the NIHR, or the Department of Health and Social Care. The NIHR had no role in the design and conduct of the study; the collection, management, analysis, and interpretation of the data; the preparation, review, or approval of the manuscript; or the decision to submit the manuscript for publication.

Competing Interests

None declared

Patient consent for publication

Not required

Provenance and peer review

For peer review only

Not commissioned; externally peer reviewed

Data availability statement

All data relevant to the study are included in the article or as online supplementary material.

For peer review only

Table 1 - Characteristics of included studies

Source, Year	Country	Study period	Study design	Implant	Training status terminology	Follow up (years)	Number of cases (trainee)	Implant brand (stem/cup if hip)	Sex (%female)	Mean age (SD or range)	Indication (%OA)	Supervision reported	Survival analysis	Revision rates reported	ROBINS-I overall risk of bias†
Hasegawa,[27] 2015	Japan	2006-10	PC	THR	Trainee vs. instructor	5	483 (259)	Multiple	-	61.3 (SD 11.6)	-	No	Yes	No	Serious
Jain,[26] 2018	UK	2005-12	RC	THR	Trainee vs. consultant	5, 10	1082 (348)	Corail/multiple	61.3	69.2 (21-94)	91	No	Yes (Add.)	Yes	Moderate
Muller,[25] 2013	Switzerland	2005-06	RC	THR	Junior vs. senior	5	130 (43)	Quadra-H /Versafit-CC	52	64 (SD 12.36)	86	No	Yes	Yes	Serious
Palan,[12] 2009	UK	1999-02	RC	THR	Trainee vs. consultant trainer	5	1501 (528)	Exeter/multiple	-	68.4 (21-94)	-	No	No	Yes	Moderate
Reidy,[13] 2016	UK	2003-04	RC	THR	Trainee vs. consultant	10	870 (286)	Multiple	60.5	69.5 (37-94)	94.8	Yes	Yes (no CIs)	Yes	Moderate
Faulkner,[15] 2017	UK	2003-04	RC	TKR	Trainee vs. consultant	5, 10	686 (236)	Multiple	-	69.9 (30-94)	93.1	No	Yes (Add.)	Yes	Moderate
Hernigou,[28] 2009	France	1990-95	RC	TKR	Young (<30) vs. senior	10, 15	250 (150)	Ceraver Hermes	69.7	73 (46-88)	-	No	Yes	No	Serious
Bottomley,[16] 2016	UK	1998-08	RC	UKR	Trainee vs. consultant	10	1084 (673)	Oxford	51.4	66.5 (SD 9.6)	100	Yes	Yes	Yes	Moderate

PC, prospective cohort; RC, retrospective cohort; Add., additional data provided by author; CIs, confidence intervals; SD, standard deviation; † see supplementary table 3 for full risk of bias assessment

For peer review only - http://bmjopen.bmj.com/site/about/guidelines.xhtml

Table 2 – GRADE Summary of Findings Table

Outcomes	Follow up (years)	Trainee revision/cases†, n	Consultant revisions/cases†, n	Net survival/relative risk (95% CI)	Participants (studies), n	Quality of Evidence	Comments
THR: net implant survival	5	650	1045	NS: Trainee 97.9% (96.6 to 99.2)<br>NS: Consultant 98.1% (97.1 to 99.2)	1695 (3)[25-27]	Very low	Serious ROB, indirectness, and imprecision
	10	348	734	NS: Trainee 98.1% (95.9 to 99.1)<br>NS: Consultant 96.7% (94.7 to 97.9)	1082 (1)[26]	Low	Serious indirectness and imprecision
THR: crude revision rate	5	13/919	29/1794	RR: 0.88 (0.46 to 1.70)	2713 (3)[12 25 26]	Very low	Serious ROB, indirectness, and imprecision
	10	13/634	40/1318	RR: 0.68 (0.37 to 1.26)	1952 (2)[13 26]	Low	Serious indirectness and imprecision
TKR: net implant survival	5	236	450	NS: Trainee 97.9% (95.0 to 99.2)<br>NS: Consultant 95.4% (93.0 to 97.0)	686 (1)[15]	Low	Serious imprecision
	10	386	550	NS: Trainee 96.2% (94.0 to 98.4)<br>NS: Consultant 95.1% (93.0 to 97.2)	936 (2)[15 28]	Very low	Serious inconsistency and imprecision
	15	150	100	NS: Trainee 91.0% (85.0 to 97.0)<br>NS: Consultant 92.0% (90.0 to 94.0)	250 (1)[28]	Very low	Serious inconsistency and very serious imprecision
TKR: crude revision rate	5	5/236	20/450	RR: 0.47 (0.18 to 1.25)	686 (1)[15]	Low	Serious imprecision
	10	8/236	26/450	RR: 0.58 (0.27 to 1.27)	686 (1)[15]	Low	Serious imprecision
	15	4/150	4/100	RR: 0.67 (0.17 to 2.60)	250 (1)[28]	Very low	Serious inconsistency and very serious imprecision
UKR: net implant survival	10	673	411	NS: Trainee 93.0% (90.3 to 95.7)<br>NS: Consultant 93.9% (90.2 to 97.6)	1084 (1)[16]	Low	Serious imprecision
UKR: crude revision rate	10	31/673	15/411	RR: 1.26 (0.69 to 2.31)	1084 (1)[16]	Low	Serious imprecision

GRADE, Grading of Recommendations Assessment, Development and Evaluation; CI, confidence interval; NS, net survival; RR, relative risk; †, number of revisions not reported for net implant survival; ROB, risk of bias

For peer review only

For peer review only - http://bmjopen.bmj.com/site/about/guidelines.xhtml

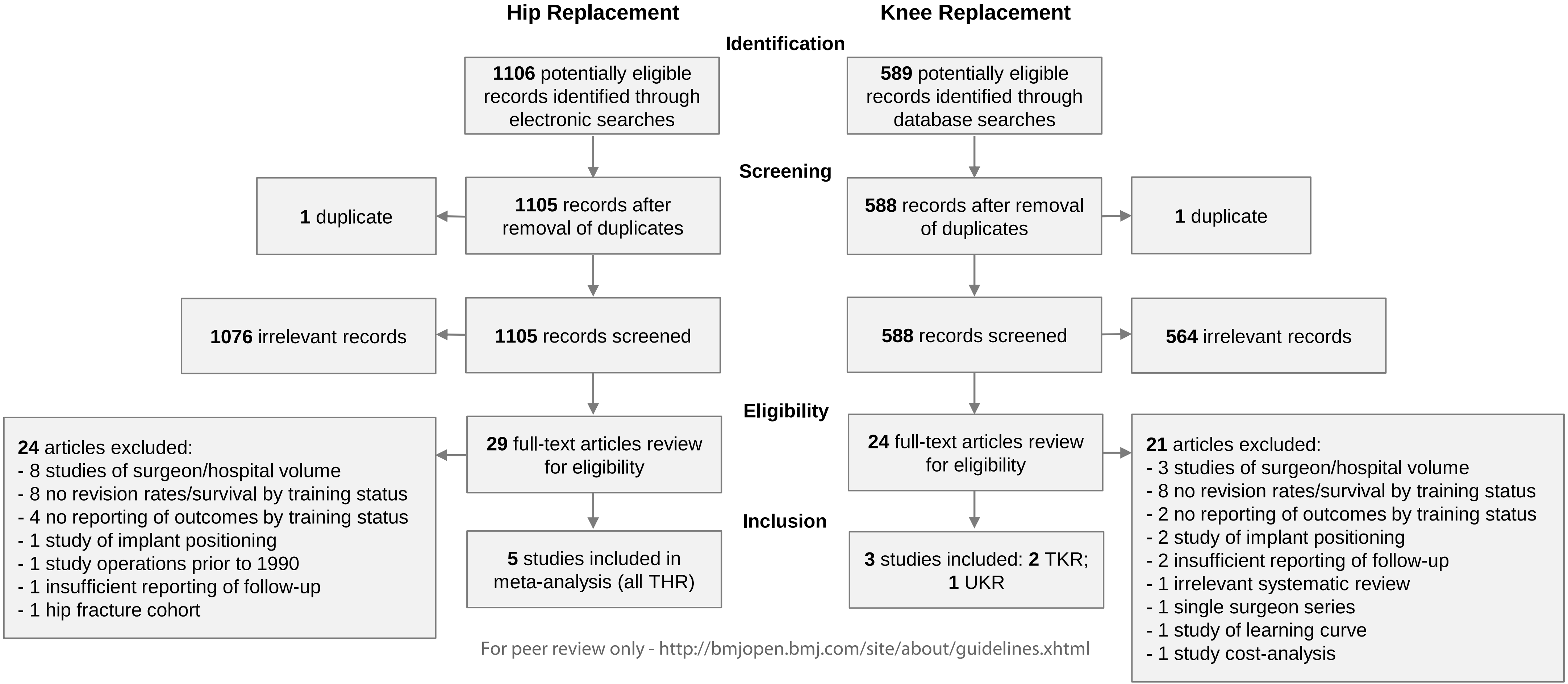
Hip Replacement
Knee Replacement
Identification
1106 potentially eligible records identified through electronic searches
589 potentially eligible records identified through database searches
Screening
1 duplicate
1105 records after removal of duplicates
588 records after removal of duplicates
1 duplicate
1076 irrelevant records
1105 records screened
588 records screened
564 irrelevant records
Eligibility
24 articles excluded:
- 8 studies of surgeon/hospital volume
- 8 no revision rates/survival by training status
- 4 no reporting of outcomes by training status
- 1 study of implant positioning
- 1 study operations prior to 1990
- 1 insufficient reporting of follow-up
- 1 hip fracture cohort
29 full-text articles review for eligibility
24 full-text articles review for eligibility
21 articles excluded:
- 3 studies of surgeon/hospital volume
- 8 no revision rates/survival by training status
- 2 no reporting of outcomes by training status
- 2 study of implant positioning
- 2 insufficient reporting of follow-up
- 1 irrelevant systematic review
- 1 single surgeon series
- 1 study of learning curve
- 1 study cost-analysis
Inclusion
5 studies included in meta-analysis (all THR)
3 studies included: 2 TKR; 1 UKR

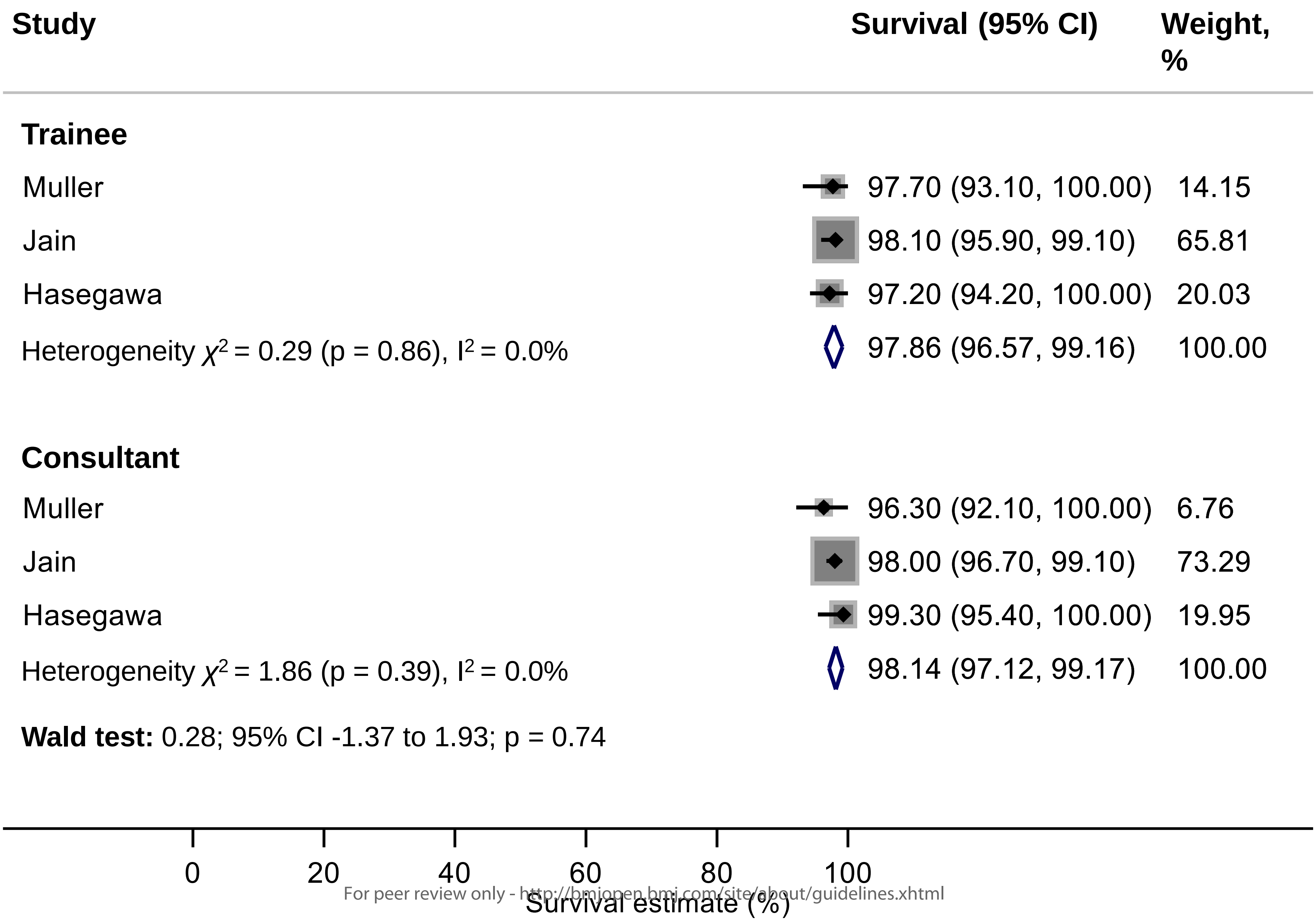

For peer review only - http://bmjopen.bmj.com/site/about/guidelines.xhtml

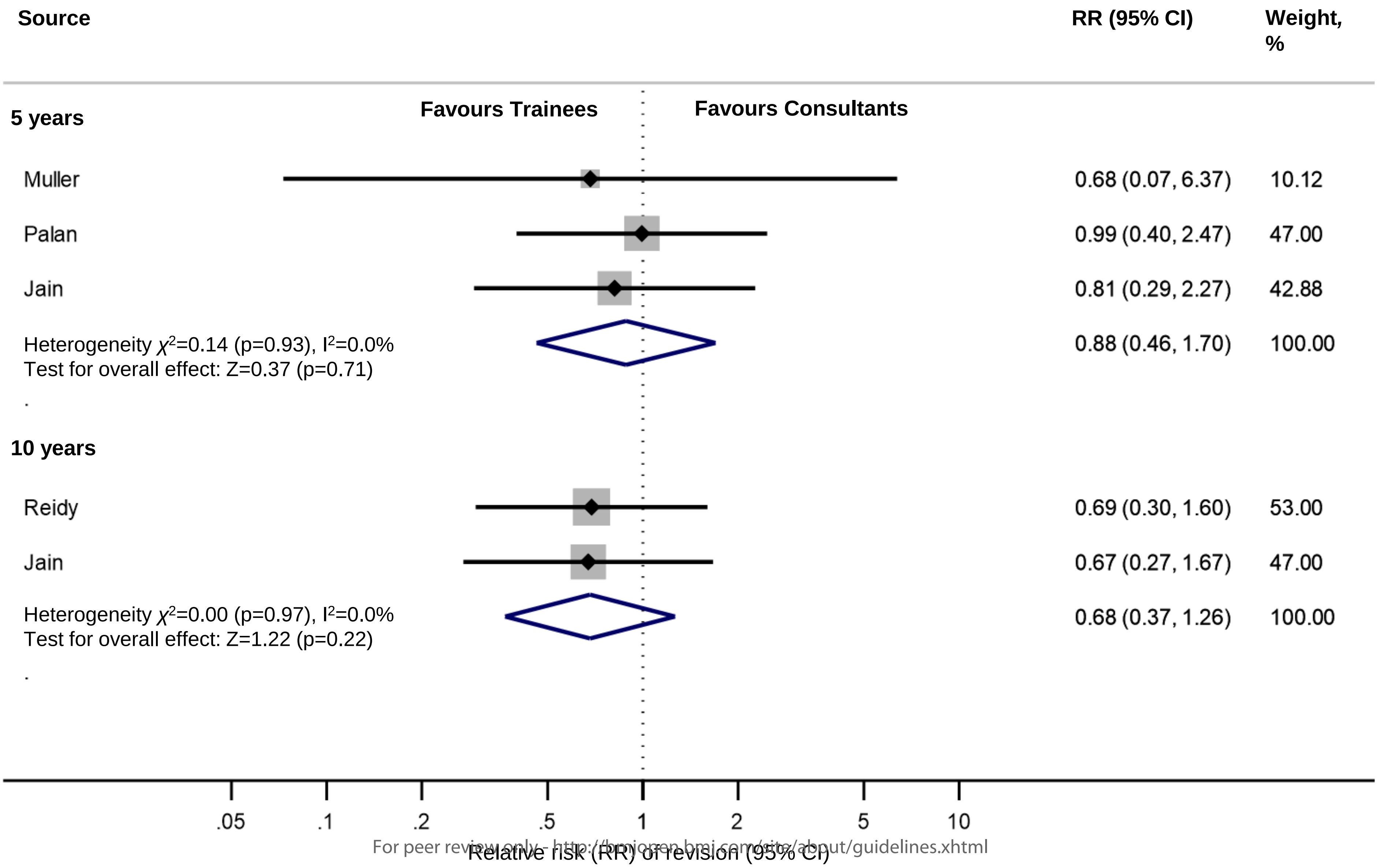
Source
RR (95% CI)
Weight, %
5 years
Favours Trainees
Favours Consultants
Muller
0.68 (0.07, 6.37)
10.12
Palan
0.99 (0.40, 2.47)
47.00
Jain
0.81 (0.29, 2.27)
42.88
Heterogeneity χ2=0.14 (p=0.93), I2=0.0%
Test for overall effect: Z=0.37 (p=0.71)
0.88 (0.46, 1.70)
100.00
10 years
Reidy
0.69 (0.30, 1.60)
53.00
Jain
0.67 (0.27, 1.67)
47.00
Heterogeneity χ2=0.00 (p=0.97), I2=0.0%
Test for overall effect: Z=1.22 (p=0.22)
0.68 (0.37, 1.26)
100.00
.05
.1
.2
.5
1
2
5
10
Relative risk (RR) of revision (95% CI)

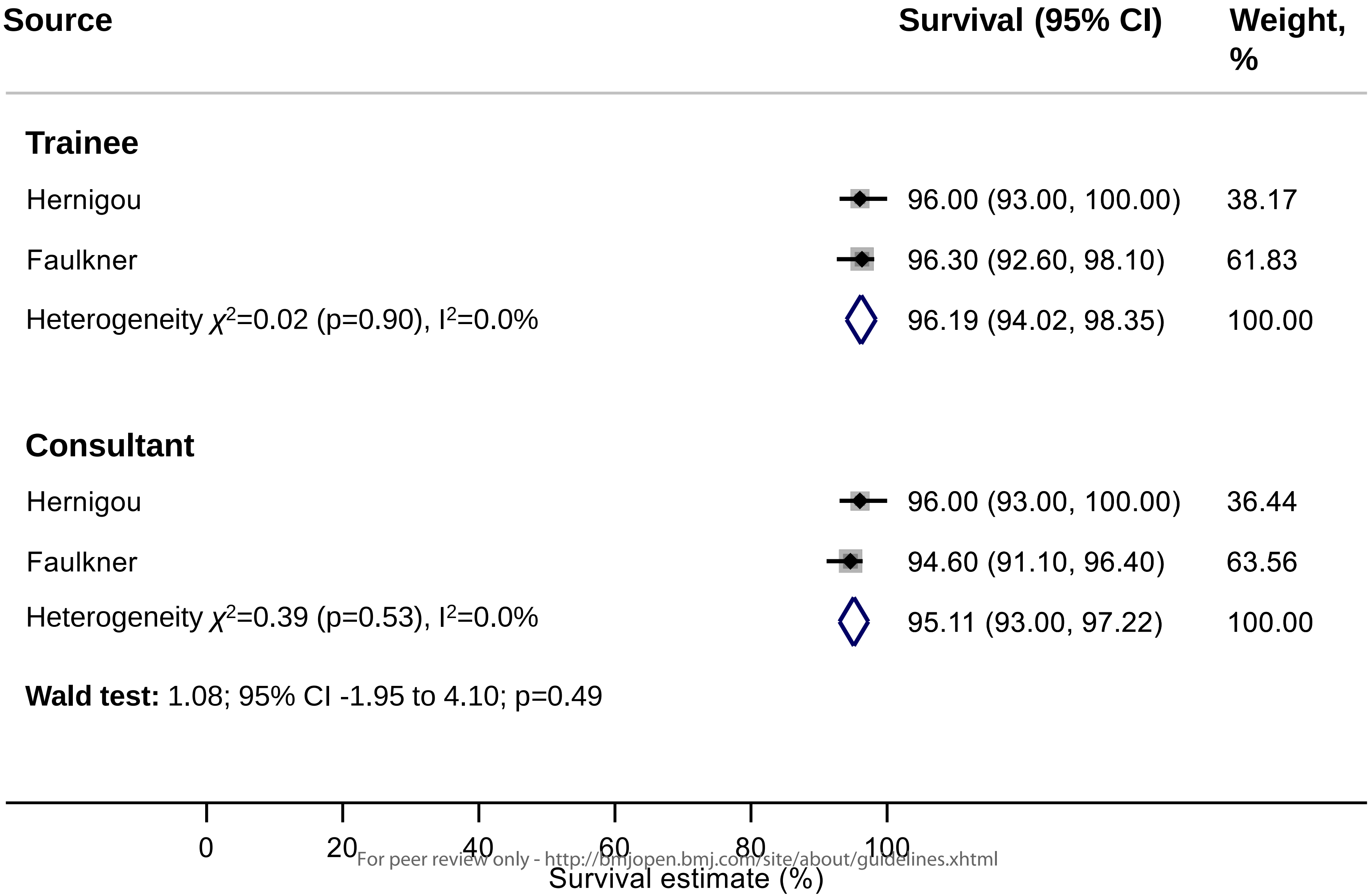

For peer review only - http://bmjopen.bmj.com/site/about/guidelines.xhtml

Online Supplementary Material

Contents:

Supplementary Methods

- Methods (page 2-3): Search strategy using Ovid (Medline + Embase)
- Methods (page 4): Reasons for Exclusion

Supplementary Table

- Table 3 (page 5): Risk of Bias (ROBINS-I) assessment of methodological quality

For peer review only

For peer review only - http://bmjopen.bmj.com/site/about/guidelines.xhtml

Methods: Search strategy using Ovid (Medline + Embase). Performed by TF & AS.

Hip Search

Hip replacement

Hip Prosthesis/ OR Arthroplasty, Replacement, Hip/ OR

(hip adj2 arthroplast$.mp) OR (hip adj2 replacement?.mp) OR (hip adj2 prosthes$.mp) OR

THA.mp OR THR.mp OR (TJR$.mp AND hip$.mp)

AND

Training

exp Education, Medical/ OR exp Inservice Training/ OR Clinical Competence/ OR

training.mp OR trainee.mp OR

experience.mp OR

junior.mp OR

senior$.mp OR

(surgeon adj2 grade).mp OR

consultant.mp OR attending?.mp OR registrar.mp OR SpR.mp OR StR.mp OR ST?.mp OR residen$.mp OR fellow$.mp OR intern.mp OR

(house adj2 officer).mp OR (foundation adj2 doctor).mp

AND

Survival

exp Prosthesis Failure/ OR exp Survival Analysis/ OR Reoperation/ OR

cox.mp OR proportional?hazard?.mp OR proportional hazard?.mp OR

cumulative?incidence?function.mp OR cumulative incidence function.mp OR CIF.mp OR

failure.mp OR

survival.mp OR survivor?ship.mp OR

revision?.mp OR

re?operation.mp OR re operation.mp OR

Kaplan?meier.mp OR Kaplan meier.mp OR KM.mp OR

product?limit?method.mp OR product limit method.mp

AND

Case-series

exp Cohort Studies/ OR Controlled Clinical Trials

follow?up.mp OR follow up.mp OR series.mp OR cohort.mp OR observational.mp OR longitudinal.mp OR prospective.mp OR retrospective.mp OR registry.mp OR registries.mp

For peer review only - http://bmjopen.bmj.com/site/about/guidelines.xhtml

Knee search

Knee replacement

Knee Prosthesis/ OR Arthroplasty, Replacement, Knee/ OR

(knee adj2 arthroplast$.mp) OR (knee adj2 replacement?.mp) OR (knee adj2 prosthes$.mp) OR

TKA.mp OR TKR.mp OR (TJR$.mp AND knee$.mp) OR

UKA.mp OR UKR.mp

AND

Training

exp Education, Medical/ OR exp Inservice Training/ OR Clinical Competence/ OR

training.mp OR trainee.mp OR

experience.mp OR

junior.mp OR

senior$.mp OR

(surgeon adj2 grade).mp OR

consultant.mp OR attending?.mp OR registrar.mp OR SpR.mp OR StR.mp OR ST?.mp OR residen$.mp OR fellow$.mp OR intern.mp OR

(house adj2 officer).mp OR (foundation adj2 doctor).mp

AND

Survival

exp Prosthesis Failure/ OR exp Survival Analysis/ OR Reoperation/ OR

cox.mp OR proportional?hazard?.mp OR proportional hazard?.mp OR

cumulative?incidence?function.mp OR cumulative incidence function.mp OR CIF.mp OR

failure.mp OR

survival.mp OR survivor?ship.mp OR

revision?.mp OR

re?operation.mp OR re operation.mp OR

Kaplan?meier.mp OR Kaplan meier.mp OR KM.mp OR

product?limit?method.mp OR product limit method.mp

AND

Case-series

exp Cohort Studies/ OR Controlled Clinical Trials

follow?up.mp OR follow up.mp OR series.mp OR cohort.mp OR observational.mp OR longitudinal.mp OR prospective.mp OR retrospective.mp OR registry.mp OR registries.mp

For peer review only - http://bmjopen.bmj.com/site/about/guidelines.xhtml

Reasons for Exclusion – Hip Papers	
**First author/Year of study**	**Reason for Exclusion**
De Vries, 2011	Principally a study of surgeon/hospital volume
Fender, 2003	Principally a study of surgeon/hospital volume
Hooper, 2009	Principally a study of surgeon/hospital volume
Johnsson, 1994	Principally a study of surgeon/hospital volume
Namba, 2012	Principally a study of surgeon/hospital volume
Ravi, 2014	Principally a study of surgeon/hospital volume
Canadian Arthroplasty Soc., 2013	Principally a study of surgeon/hospital volume
MacBride, 2010	Principally a study of surgeon/hospital volume
Enocson, 2009	No revision rates/survival analysis reported according to grade
Field, 2006	No revision rates/survival analysis reported according to grade
Leguerrand, 2018	No revision rates/survival analysis reported according to grade
Moran, 2004	No revision rates/survival analysis reported according to grade
Smith, 2018	No revision rates/survival analysis reported according to grade
Wilson, 2016	No revision rates/survival analysis reported according to grade
Wroblewski, 1998	No revision rates/survival analysis reported according to grade
Schoenfeld, 2013	No revision rates/survival analysis reported according to grade
Inglis, 2013	Insufficient reporting of follow-up
Marston, 1996	Study of operations performed prior to 1990
Khatod, 2014	No reporting of outcomes according to training status
Whitehouse, 2014	No reporting of outcomes according to training status
Williams, 2002	No reporting of outcomes according to training status
Zwartele, 2005	No reporting of outcomes according to training status
Kim, 2017	Principally a study of implant positioning
MacDonald, 2020	Hip fracture cohort; insufficient follow-up
N.B. Multiple reasons for some papers	

Reasons for Exclusion – Knee Papers	
**First author/Year of study**	**Reason for Exclusion**
Bini, 2013	Principally a study of surgeon/hospital volume
Namba, 2012	Principally a study of surgeon/hospital volume
Zambianchi, 2014	Principally a study of surgeon/hospital volume
Liddle, 2014	No revision rates/survival analysis reported according to grade
Beattie, 2016	No revision rates/survival analysis reported according to grade
Haughom, 2014	No revision rates/survival analysis reported according to grade
Khakha, 2015	No revision rates/survival analysis reported according to grade
Schoenfeld, 2013	No revision rates/survival analysis reported according to grade
Windisch, 2017	No revision rates/survival analysis reported according to grade
Wilson, 2016	No revision rates/survival analysis reported according to grade
Woolson, 2007	No revision rates/survival analysis reported according to grade
Atrey, 2014	No reporting of outcomes according to training status
Back, 2000	No reporting of outcomes according to training status
Gaillard, 2016	Principally a study of implant positioning
Mahaluxmivala, 2001	Principally a study of implant positioning
Storey, 2018	Insufficient reporting of follow-up
Theelen, 2018	Insufficient reporting of follow-up
Jasper, 2016	Irrelevant systematic review
Lacko, 2018	Single surgeon series
Matas-Diez, 2018	Principally a study of learning curve
Lavernia, 2000	Study of cost-analysis
N.B. Multiple reasons for some papers	

For peer review only - http://bmjopen.bmj.com/site/about/guidelines.xhtml

Supplementary Table 3: Risk of Bias (ROBINS-I) assessment

ROBINS-I	Bottomley, 2016	Faulkner, 2017	Hernigou, 2009	Hasegawa, 2015	Jain, 2018	Muller, 2013	Palan, 2009	Reidy, 2016
Bias due to confounding	⊕⊕	⊕	⊕⊕	⊕⊕	⊕	⊕⊕	⊕	⊕
Bias in selection of patients	⊕	⊕	⊕	⊕	⊕	⊕	⊕	⊕
Bias in classification of interventions	⊖	⊕	⊕	⊕⊕	⊕	⊕⊕	⊕	⊕
Bias due to deviations from interventions	⊕	⊕	⊕⊕	⊕⊕	⊕	⊕⊕	⊕	⊕
Bias due to missing data	⊖	⊕	⊕⊕	⊕⊕	⊕	⊕	⊖	⊕
Bias in measurement of outcome	⊖	⊕	⊖	⊕	⊖	⊖	⊖	⊕
Bias in selection of the reported result	⊖	⊖	⊖	⊕	⊖	⊕	⊖	⊖
Overall risk of Bias	⊕	⊕	⊕⊕	⊕⊕	⊕	⊕⊕	⊕	⊕

Key: ⊖ = low risk of bias; ⊕ = moderate risk of bias; ⊕⊕ = serious risk of bias; ⊕⊕⊕ = critical risk of bias

For peer review only

For peer review only - http://bmjopen.bmj.com/site/about/guidelines.xhtml

MOOSE Checklist for Meta-analyses of Observational Studies

Item No	Recommendation	Reported on Page No
Reporting of background should include		
1	Problem definition	4-5
2	Hypothesis statement	4-5
3	Description of study outcome(s)	4
4	Type of exposure or intervention used	4
5	Type of study designs used	6
6	Study population	6-7
Reporting of search strategy should include		
7	Qualifications of searchers (e.g., librarians and investigators)	Online supplementary methods
8	Search strategy, including time period included in the synthesis and key words	6 & online supplementary methods
9	Effort to include all available studies, including contact with authors	7, 9
10	Databases and registries searched	7
11	Search software used, name and version, including special features used (e.g., explosion)	7-8
12	Use of hand searching (e.g., reference lists of obtained articles)	6
13	List of citations located and those excluded, including justification	Online supplementary methods
14	Method of addressing articles published in languages other than English	6
15	Method of handling abstracts and unpublished studies	6-7
16	Description of any contact with authors	7, 10-12
Reporting of methods should include		
17	Description of relevance or appropriateness of studies assembled for assessing the hypothesis to be tested	6-8
18	Rationale for the selection and coding of data (e.g., sound clinical principles or convenience)	6-8
19	Documentation of how data were classified and coded (e.g., multiple raters, blinding and interrater reliability)	7-8
20	Assessment of confounding (e.g., comparability of cases and controls in studies where appropriate)	8
21	Assessment of study quality, including blinding of quality assessors, stratification or regression on possible predictors of study results	8
22	Assessment of heterogeneity	8
23	Description of statistical methods (e.g., complete description of fixed or random effects models, justification of whether the chosen models account for predictors of study results, dose-response models, or cumulative meta-analysis) in sufficient detail to be replicated	8-9
24	Provision of appropriate tables and graphics	Table 1-2, Figures 1-4
Reporting of results should include		
25	Graphic summarizing individual study estimates and overall estimate	Figures 2-4
26	Table giving descriptive information for each study included	Table 1-2
27	Results of sensitivity testing (e.g., subgroup analysis)	N/A, justification

For peer review only

		13-14
28	Indication of statistical uncertainty of findings	10-15

Item No	Recommendation	Reported on Page No
Reporting of discussion should include		
29	Quantitative assessment of bias (e.g., publication bias)	10
30	Justification for exclusion (e.g., exclusion of non-English language citations)	13-14
31	Assessment of quality of included studies	10, Table 2 & supplementary Table 3
Reporting of conclusions should include		
32	Consideration of alternative explanations for observed results	13-15
33	Generalization of the conclusions (i.e., appropriate for the data presented and within the domain of the literature review)	13-15
34	Guidelines for future research	15
35	Disclosure of funding source	20

From: Stroup DF, Berlin JA, Morton SC, et al, for the Meta-analysis Of Observational Studies in Epidemiology (MOOSE) Group. Meta-analysis of Observational Studies in Epidemiology. A Proposal for Reporting. *JAMA*. 2000;283(15):2008-2012. doi: 10.1001/jama.283.15.2008.

For peer review only

BMJ Open

The association between surgeon training status and implant survival following hip and knee replacement: a systematic review and meta-analysis

Journal:	*BMJ Open*
Manuscript ID	bmjopen-2020-047882.R1
Article Type:	Original research
Date Submitted by the Author:	12-May-2021
Complete List of Authors:	Fowler, Timothy; University of Bristol Medical School, Musculoskeletal Research Unit<br>Aquilina, Alex; University of Bristol Medical School, Musculoskeletal Research Unit<br>Blom, AW; University of Bristol Medical School, Musculoskeletal Research Unit; National Institute for Health Research Bristol Biomedical Research Centre, University Hospitals Bristol NHS Foundation Trust, University of Bristol<br>Sayers, Adrian; University of Bristol Medical School, Musculoskeletal Research Unit<br>Whitehouse, Michael; University of Bristol Medical School, Musculoskeletal Research Unit; National Institute for Health Research Bristol Biomedical Research Centre, University Hospitals Bristol NHS Foundation Trust, University of Bristol
**Primary Subject Heading**:	Surgery
Secondary Subject Heading:	Medical education and training, Health policy, Evidence based practice
Keywords:	Hip < ORTHOPAEDIC & TRAUMA SURGERY, Knee < ORTHOPAEDIC & TRAUMA SURGERY, Orthopaedic & trauma surgery < SURGERY, MEDICAL EDUCATION & TRAINING, Health policy < HEALTH SERVICES ADMINISTRATION & MANAGEMENT

SCHOLARONE™
Manuscripts

I, the Submitting Author has the right to grant and does grant on behalf of all authors of the Work (as defined in the below author licence), an exclusive licence and/or a non-exclusive licence for contributions from authors who are: i) UK Crown employees; ii) where BMJ has agreed a CC-BY licence shall apply, and/or iii) in accordance with the terms applicable for US Federal Government officers or employees acting as part of their official duties; on a worldwide, perpetual, irrevocable, royalty-free basis to BMJ Publishing Group Ltd ("BMJ") its licensees and where the relevant Journal is co-owned by BMJ to the co-owners of the Journal, to publish the Work in this journal and any other BMJ products and to exploit all rights, as set out in our licence.

The Submitting Author accepts and understands that any supply made under these terms is made by BMJ to the Submitting Author unless you are acting as an employee on behalf of your employer or a postgraduate student of an affiliated institution which is paying any applicable article publishing charge ("APC") for Open Access articles. Where the Submitting Author wishes to make the Work available on an Open Access basis (and intends to pay the relevant APC), the terms of reuse of such Open Access shall be governed by a Creative Commons licence – details of these licences and which Creative Commons licence will apply to this Work are set out in our licence referred to above.

Other than as permitted in any relevant BMJ Author's Self Archiving Policies, I confirm this Work has not been accepted for publication elsewhere, is not being considered for publication elsewhere and does not duplicate material already published. I confirm all authors consent to publication of this Work and authorise the granting of this licence.

For peer review only

The association between surgeon training status and implant survival following hip and knee replacement: a systematic review and meta-analysis

Authors

Timothy J. Fowler, MRCS[1]

Alex L. Aquilina, MRCS[1]

Ashley W. Blom, PhD[1,2]

*Adrian Sayers, MSc[1]

*Michael R. Whitehouse, PhD[1,2]

**Joint Senior Authors*

Author Affiliations

[1]Musculoskeletal Research Unit, Translational Health Sciences, Bristol Medical School, Southmead Hospital, Bristol, UK

National Institute for Health Research Bristol Biomedical Research Centre, University Hospitals Bristol NHS Foundation Trust, University of Bristol, Bristol, UK

Corresponding Author

Mr Timothy J Fowler, BMBCh (Oxon) MA MRCS

Address: Musculoskeletal Research Unit, Translational Health Sciences, Bristol Medical School, Southmead Hospital, Bristol, UK, BS10 5NB

Email: t.j.fowler@bristol.ac.uk; *Phone:* +44 (0)117 414 7865

Word count

3121

For peer review only - http://bmjopen.bmj.com/site/about/guidelines.xhtml

For peer review only

Abstract

Objective: To investigate the association between the training status of the surgeon (i.e. trainee vs. consultant) and implant survival following primary hip and knee replacement.

Design: A systematic review and meta-analysis of observational studies.

Data sources: MEDLINE® and Embase® from inception until December 10th, 2020.

Setting: Units performing primary hip and/or knee replacements since 1990.

Participants: Adult patients undergoing either a primary hip or knee replacement, predominantly for osteoarthritis.

Intervention: Whether the surgeon recorded as performing the procedure was a trainee or not.

Primary and secondary outcome measures: The primary outcome was net implant survival (i.e. absence of revision surgery), reported as a Kaplan-Meir survival estimate. The secondary outcome was crude revision rate. Both outcomes were reported according to the training status of the surgeon.

Results: Eight cohort studies capturing 4066 total hip replacements (THRs), 936 total knee replacements (TKRs), and 1084 unicompartmental knee replacements (UKRs) were included. The pooled net implant survival estimates for THRs at five years follow up were 97.9% (95% CI 96.6 to 99.2) for trainees and 98.1% (95% CI 97.1 to 99.2) for consultants. The relative risk of revision of THRs at five and ten years was 0.88 (95% CI 0.46 to 1.70) and 0.68 (95% CI 0.37 to 1.26), respectively. For TKRs, the net implant survival estimates at ten years follow up were 96.2% (95% CI 94.0 to 98.4) for trainees and 95.1% (95% CI 93.0 to 97.2) for consultants.

Conclusions: There is no strong evidence in the existing literature that trainee surgeons have worse outcomes compared to their consultant colleagues, in terms of the net survival or crude revision rate of hip and knee replacements at five to ten years follow up. These findings are applicable to countries with established orthopaedic training programmes.

For peer review only

For peer review only - http://bmjopen.bmj.com/site/about/guidelines.xhtml

Article Summary

Strengths and limitations of this study

- To our knowledge, this is the first meta-analysis of the association between surgeon training status and implant survival following hip and knee replacement.
- We performed a comprehensive systematic review according to current best practice guidelines.
- The findings of this review are limited by the strength of the existing published data from a relatively small number of predominantly retrospective observational studies.

For peer review only

For peer review only - http://bmjopen.bmj.com/site/about/guidelines.xhtml

Introduction

Hip and knee replacements are effective surgical interventions for the treatment of end stage degenerative conditions of the hip and knee.[1, 2] More than 200,000 are performed per year in the United Kingdom alone.[3] These procedures are performed by surgeons at various stages in their training, with varying levels of senior supervision. Contemporary training practices must ensure a balance between protecting development opportunities for the next generation of surgeons, while limiting the exposure of patients to unnecessary risk during the training process.

Implant survival, which is determined by the absence of revision surgery, is an important and commonly used measure of surgical performance.[4, 5] Net survival estimates are calculated using statistical methods of survival analysis (e.g. Kaplan-Meier analysis), which look at time to a defined failure 'event' (e.g. revision) and account for censored data that arise due to incomplete follow up, or death.[6] Another commonly reported metric is crude revision rate, which is defined as the observed number of failure events in a specified period of time.

The survival of hip and knee replacements according to the training status of the surgeon is poorly understood. Higher rates of complications and longer operative times have been identified in orthopaedic procedures performed by trainees.[7, 8] Radiographic studies comparing trainee and consultant joint replacement have identified differences in acetabular anteversion,[9] hip centre of rotation,[10] and various measures of knee replacement component positioning.[11] However, the causative impact of these findings on implant survival has not been established. It has been suggested that when trainees are appropriately supervised, they can obtain comparable functional outcomes and implant survivorship to their consultant colleagues when performing total hip replacement (THR),[12-14] total knee replacement (TKR)[15] and unicompartmental knee replacement (UKR).[16]

The aim of this study was to conduct a systematic review and meta-analysis using the existing literature on the association between the training status of the surgeon (trainee vs. consultant) and implant survival outcomes in hip and knee replacement surgery.

For peer review only - http://bmjopen.bmj.com/site/about/guidelines.xhtml

Methods

This review was conducted using methods described in the Cochrane Handbook for Systematic Reviews of Interventions, with reporting in accordance with the Meta-analyses Of Observational Studies in Epidemiology (MOOSE) checklist.[17, 18] The study was registered with the PROSPERO database at inception (CRD42019150494).

Data sources and search strategy

We searched for cohort studies reporting implant survival estimates and/or revision rates of hip or knee replacements, according to the training status of the surgeon. Separate searches were performed for hips and knees. We conducted searches of MEDLINE® and Embase™ from inception until December 10th, 2020. Searches used keywords and MeSH (Medical Subject Headings) terms relating to hip and knee replacement, implant survival, revision surgery and surgeon training status (see online supplementary methods). There were no language restrictions. Titles and abstracts of potentially relevant non-English language citations were translated. We manually screened the bibliographies of full text articles and used Web of Science™ citation tracking to identify additional relevant studies.

Eligibility criteria

We included studies if they involved predominantly unselected adult patients (≥ 18 years old) undergoing primary hip or knee replacement (including THR, TKR, UKR and hip resurfacing), predominantly for the treatment of osteoarthritis. Included articles needed to report the primary and/or secondary outcome measure for two different groups of surgeons according to their training status (e.g. trainee vs. consultant). We defined a minimum follow up of five years and articles that did not clearly define the length of follow up were excluded. For example, we excluded studies reporting the revision rate ‘per 100 component years’, as these did not explicitly define the length of follow up. We excluded studies in which the index operation was performed prior to 1990; thereby, including studies that are representative of contemporary training practices, but also allowing for inclusion of studies reporting up to 30 years of follow up.

For peer review only - http://bmjopen.bmj.com/site/about/guidelines.xhtml

Primary exposure

The primary exposure was whether the surgeon recorded as performing the procedure was a trainee or not. The 'training status' of the surgeon is a measure of the designated level of surgical experience and seniority, which we considered to be a binary variable: either 'trainee', or 'consultant'. Consultant surgeons have completed their formal training in orthopaedic surgery and have been appointed to a senior position in which they can practice independently and supervise trainee surgeons. The term 'consultant' is used synonymously with 'attending surgeon' in many healthcare settings including the United States. Additional terms used to describe this variable were deemed eligible during screening (e.g. Trainee: registrar; resident; junior/young surgeon; fellow. Consultant: attending; senior surgeon; trainer).

Outcome measures

The primary outcome was net implant survival, reported as a Kaplan-Meir survival estimate. The secondary outcome measure was crude revision rate, which was defined as the observed number of revisions in a specified period of time.

Screening and data extraction

Two authors (TJF and ALA) independently screened all titles and abstracts of journal articles using Rayyan (Rayyan QCRI, Doha).[19] Cases of disagreement were resolved through re-review and consensus. Full texts of potentially relevant studies were reviewed in detail and disagreements on final inclusion were resolved through discussion with a senior author (MRW).

Data were extracted in duplicate using a standardised proforma. We recorded data on the following: healthcare setting, study period, implant type, age, sex, indication, level of supervision, crude revision rate, and net implant survival estimates (including confidence intervals [CI]). Life tables were reviewed, and estimates were extracted for all available five-year intervals of follow up. Discrepancies in data collection were resolved through re-review and consensus. Where survival

For peer review only - http://bmjopen.bmj.com/site/about/guidelines.xhtml

estimates, CIs and revision rates were incompletely reported, we contacted corresponding authors to request missing data.

Risk of bias and quality of evidence assessment

The risk of bias was assessed using the Cochrane ROBINS-I tool for the risk of bias in non-randomised cohort studies.[20] We assessed the quality of evidence for each outcome using the Grading of Recommendations Assessment, Development and Evaluation (GRADE) approach, which considers the imprecision, inconsistency, indirectness, and risk of bias in a body of evidence.[21]

Statistical analysis

Statistical analysis was performed using Stata (Version SE 15.1; StataCorp, Texas). For the primary outcome measure of net implant survival, we performed separate meta-analyses for each implant type, by training status and length of follow up. We pooled survival estimates, assuming that survivorship approximated risk, with fixed effects meta-analysis weighting each study on the overall pooled estimate according to its standard error, which was calculated from published CIs; a method described by Evans *et al*.[4, 5] The effect size (survival) for trainees and consultants, was compared using a Wald test. For the secondary outcome measure, we derived and meta-analysed the relative risk (RR) of revision for each implant type by training status and length of follow up. We used a fixed effects model using the Mantel-Haenszel method.[22] Heterogeneity was assessed with chi-squared tests, with I^2 used to quantify inconsistency.[23] Publication bias was assessed by inspecting funnel plot symmetry.[24]

Patient and public involvement

There was no direct patient or public involvement in the design or conduct of this review.

For peer review only - http://bmjopen.bmj.com/site/about/guidelines.xhtml

Results

Separate searches for hip and knee replacements identified 1106 and 589 articles, respectively. After removal of duplicates and abstract screening, 29 hip papers and 24 knee papers remained. Through review of full text articles, we identified five hip and three knee studies eligible for inclusion. This process of review is summarised as a flow diagram in figure 1 and the characteristics of included studies are summarised in table 1. Five studies were conducted in the UK, with the remaining three studies originating from France, Switzerland, and Japan.

Risk of bias assessment

Supplementary table 3 provides a summary of the ROBINS-I assessment, which indicates a moderate to severe risk of bias in all studies. Funnel plot asymmetry and statistical tests for funnel plot asymmetry as a means of assessing publication bias were not applicable due to the small number of studies.[25]

Hip replacement

The five included hip studies represent 1464 THRs performed by trainees and 2602 THRs performed by consultants, with follow up ranging from five to ten years. Four studies were retrospective cohort studies;[12, 13, 26, 27] one was a non-randomised prospective cohort study.[28] No articles on hip resurfacing met the inclusion criteria. One author provided additional unpublished data in the form of net survival estimates.[27] Reidy *et al* reported survival estimates, but no CIs.[13] Net survival estimates and corresponding CIs were thus extracted from three studies at five years and one study at ten years. Crude revision rates were reported in three studies at five years and two studies at ten years.

For peer review only - http://bmjopen.bmj.com/site/about/guidelines.xhtml

Primary outcome: Net implant survival (THR)

Meta-analysis showed net survivorship of 97.9% (95% CI 96.6 to 99.2) at five years for THRs performed by trainees, compared to 98.1% (95% CI 97.1 to 99.2) for THRs performed by consultants (figure 2). There was no strong evidence of an association between training status and net implant survival at this interval of follow up (Wald test: p=0.74).

Meta-analysis was not possible for the ten-year data given the availability of only one study for this timepoint. In a cohort of 1082 reverse hybrid THRs, Jain *et al* demonstrated overall 97.2% implant survival at ten years. Additional data provided by the author indicate that they found no evidence of a difference in implant survival according to the training status of the surgeon (Trainee: 98.1%; 95% CI 95.9 to 99.1; Consultant: 96.7%; 95% CI 94.7 to 97.9).[27]

Secondary outcome: Crude revision rate (THR)

Meta-analysis showed no strong evidence of an association between training status and the crude revision rate at five, or ten years. The RR of revision at five and ten years was 0.88 (95% CI 0.46 to 1.70) and 0.68 (95% CI 0.37 to 1.26), respectively (figure 3).

Knee replacement

The three knee studies represent 1059 knee replacements performed by trainees and 961 performed by consultants, with follow up ranging from ten to fifteen years. All three were retrospective cohort studies.[15, 16, 29] Only one study reported on UKRs,[16] thus further quantitative analysis was limited to the two TKR papers.[15, 29] Faulkner *et al* provided additional unpublished survival data from which we calculated corresponding CIs for their published survival estimates.[15] Net survival estimates and CIs were thus extracted from both TKR studies at ten years. Crude revision rates were only available from one study at each five-year interval of follow up.

For peer review only - http://bmjopen.bmj.com/site/about/guidelines.xhtml

Primary outcome: Net implant survival (TKR)

Meta-analysis showed net survivorship of 96.2% (95% CI 94.0 to 98.4) at ten years for TKRs performed by trainees, compared to 95.1% (95% CI 93.0 to 97.2) for TKRs performed by consultants (Fig. 4). There was no strong evidence of an association between training status and net implant survival at this interval of follow up (Wald test: p=0.49).

Secondary outcome: Crude revision rate (TKR)

Two studies reported crude revision rates according to surgeon training status; however, with data from only one study available at each interval of follow up, meta-analysis was not feasible. Instead, we provide a narrative summary. Faulkner *et al* provided additional unpublished data, which indicated crude revision rates at five years for trainees and consultants of 2.1% and 4.4%, respectively.[15] This rises to 3.4% (trainees) and 5.8% (consultants) at ten years. These data represent a RR of revision of 0.49 (95% CI 0.19 to 1.28) at five years and 0.60 (95% CI 0.28 to 1.31) at ten years. Hernigou published crude revision rates at 15 years of 2.7% for junior surgeons and 4% for senior surgeons, which represents a RR of revision of 0.68 (95% CI 0.17 to 2.64).[29]

Unicompartmental knee replacement (UKR)

A single study reported survivorship outcomes for UKRs according to training status.[16] Bottomley *et al* conducted a retrospective cohort study of 1084 consecutive UKRs. They demonstrated that consultants and trainees had cumulative 9-year survival estimates of 93.9% and 93%, respectively. They found no strong evidence of a difference in implant survival between the groups (log rank: p=0.30).[16]

Assessment of the quality of evidence

The GRADE assessment of the quality of evidence for each outcome indicates a low, or very low quality of evidence for all outcomes (table 2).

For peer review only - http://bmjopen.bmj.com/site/about/guidelines.xhtml

Discussion

The results of this study suggest that, in the context of contemporary practice, trainees do not achieve worse hip and knee replacement survival outcomes compared to their consultant colleagues at five to ten years follow up. We found no strong evidence of an association between the training status of the surgeon and the net survival of THRs at five years (trainees: 97.9% vs consultants: 98.1%). There was no association between training status and the crude revision rate of THRs at either five, or ten years follow up. Furthermore, we found no strong evidence of an association between training status and the net survival of TKRs at ten years (trainees: 96.2% vs consultants: 95.1%).

Strengths and limitations

This review has a number of strengths. We conducted a comprehensive systematic review with an exhaustive search according to current best practice guidelines and published the protocol for the methodology at inception. However, the data captured by this review have several limitations, which we have attempted to address through quality of evidence assessment and risk of bias analysis. The GRADE assessment indicates a low to very low quality of evidence for each outcome. Furthermore, the ROBINS-I assessment indicates a moderate to severe risk of bias in the included studies. These findings are consistent with the predominantly retrospective design of the included studies. The conclusions of this review are therefore limited by the strength and quality of the existing published data, which originate from a relatively small number of observational studies.

Meta-analysis of the primary outcome measure was only possible at five and ten years for THRs and ten years for TKRs, which limits the generalisability of our findings to these short and medium-term intervals of follow up. The included studies originated from the UK, France, Switzerland, and Japan, which limits the generalisability of the findings to countries with established orthopaedic training programmes.

Formal orthopaedic training is a long process (lasting up to ten years in some countries); therefore, individual trainees have varying levels of experience, which are not captured by the binary variables

For peer review only - http://bmjopen.bmj.com/site/about/guidelines.xhtml

used in this study, or in the existing literature. The included studies did not provide sufficient data to perform meaningful adjustment or sensitivity analysis according to specific training grade, or the level of senior supervision. Furthermore, our study captures cases performed between 1990 and 2012 (table 1) and we were unable to adjust for variations in training practice (such as the level of senior supervision) that may have occurred over this 22-year period.

Implant survival is a key determinant of good outcome in joint replacement surgery and is the sole variable considered in the current benchmarking strategies for the assessment of implant components. However, this review did not consider other factors that may be important when evaluating surgical outcomes, such as patient reported outcome measures, or complications other than failure, which have previously been found to occur in higher rates when joint replacements are performed by less experienced surgeons.[7, 8]

Published literature did not consistently report age, sex, comorbidities, implant design, or the level of senior supervision; making it very difficult to adjust for these variables. It is reasonable to suggest that the predominantly superior survival outcomes observed in the trainee cohorts are a product of patient selection and close senior supervision, with good trainers selecting appropriately complex cases for their trainees.

Comparison with other studies

A single study was excluded because the THRs under follow up were performed prior to 1990;[30] thus not considered representative of contemporary training practices. The authors of this ten-year study of 413 THRs reported a significantly higher rate of revision for trainees, with 15 of 16 revised hips performed by trainees. Inclusion of this study in our meta-analysis of ten-year THR crude revision rates increases the RR of revision to 1.12 (95% CI 0.66 to 1.92), in favour of THRs performed by consultants. One explanation for this is that the model of training in the UK at the time differed, with trainees more often operating without appropriate senior supervision.

For peer review only - http://bmjopen.bmj.com/site/about/guidelines.xhtml

Our findings are consistent with those of the New Zealand Joint Registry.[31, 32] In a cohort of 35 415 THRs, of which 4049 were performed by trainees, the authors reported no significant difference in the revision rate between surgeon groups.[32] In a further cohort of 79 671 TKRs and 8854 UKRs, of which approximately 10% were performed by trainees, they reported no significant difference in the revision rates of knee replacements performed by trainees and consultants.[31] These studies were not included in this meta-analysis because the authors did not report net survival estimates and revision rates were reported 'per 100 component years', rather than for clearly defined periods of follow up, which cannot be calculated from the data presented.

Implications

There is a delicate balance between ensuring optimal outcomes for patients and the necessity to train the next generation of surgeons. Reidy and Faulkner suggest that the availability of surgeon level registry data as a means of benchmarking performance, may lead to a desire to avoid perceived poor performance and thus a reluctance among consultants to let trainees operate.[13, 15] However, the findings of this review are encouraging and support the notion that in the context of contemporary practice, in countries with established and regulated orthopaedic training programmes, trainees can achieve implant survival outcomes equivalent to their consultant colleagues. The senior supervision of trainees was inconsistently reported in the studies included in this review but is likely to play an important role in the successful outcome of trainee performed hip and knee replacements.

An adequately powered non-inferiority RCT with ten years follow up assuming an acceptable revision rate of 5% and a 1% absolute non-inferiority delta ($\alpha = 0.05$; power = 0.80; 1:1 allocation ratio), would require a sample size of 6400 patients.[33] However, factors inherent to the training process, such as variation amongst trainees, the need for case selection and varying levels of supervision based on a trainee's experience, may preclude an inclusive and therefore generalisable RCT. Further investigation should focus on the associations between senior supervision, specific surgeon training grade, and the risk of revision following trainee-performed hip and knee replacements. The analysis

For peer review only - http://bmjopen.bmj.com/site/about/guidelines.xhtml

of unselected patient data recorded in a mandatory national joint replacement registry would be an appropriate means of further investigation.

Conclusions

In conclusion, there is no strong evidence in the existing literature that trainee surgeons have worse outcomes than their consultant surgeon colleagues, in terms of the net survival, or crude revision rate of hip and knee replacements at five to ten years follow up. This may mean that there is no difference, or that appropriate case mix selection and supervision of trainees is currently employed and is safe to continue. Our results are concordant with published registry data,[31, 32] and represent the best available evidence.

For peer review only

For peer review only - http://bmjopen.bmj.com/site/about/guidelines.xhtml

References

1. Ferguson RJ, Palmer AJ, Taylor A, et al. Hip replacement. Lancet. 2018;**392**(10158):1662-71.
2. Price AJ, Alvand A, Troelsen A, et al. Knee replacement. Lancet. 2018;**392**(10158):1672-82.
3. 17th Annual Report: The National Joint Registry for England, Wales, Northern Ireland and the Isle of Man. 2020.
4. Evans JT, Evans JP, Walker RW, et al. How long does a hip replacement last? A systematic review and meta-analysis of case series and national registry reports with more than 15 years of follow-up. Lancet. 2019;**393**(10172):647-54.
5. Evans JT, Walker RW, Evans JP, et al. How long does a knee replacement last? A systematic review and meta-analysis of case series and national registry reports with more than 15 years of follow-up. Lancet. 2019;**393**(10172):655-63.
6. Sayers A, Evans JT, Whitehouse MR, et al. Are competing risks models appropriate to describe implant failure? Acta Orthop. 2018;**89**(3):256-8.
7. Hedlundh U, Ahnfelt L, Hybbinette CH, et al. Surgical experience related to dislocations after total hip arthroplasty. J Bone Joint Surg Br. 1996;**78**(2):206-9.
8. Schoenfeld AJ, Serrano JA, Waterman BR, et al. The impact of resident involvement on post-operative morbidity and mortality following orthopaedic procedures: a study of 43,343 cases. Arch Orthop Trauma Surg. 2013;**133**(11):1483-91.
9. Moran M, Yap SL, Walmsley P, et al. Clinical and radiologic outcome of total hip arthroplasty performed by trainee compared with consultant orthopedic surgeons. J Arthroplasty. 2004;**19**(7):853-7.
10. Kim SC, Lim YW, Kwon SY, et al. Level of surgical experience is associated with change in hip center of rotation following cementless total hip arthroplasty: A radiographic assessment. PLoS One. 2017;**12**(5):e0178300.
11. Kazarian GS, Lawrie CM, Barrack TN, et al. The Impact of Surgeon Volume and Training Status on Implant Alignment in Total Knee Arthroplasty. J Bone Joint Surg Am. 2019;**101**(19):1713-23.
12. Palan J, Gulati A, Andrew JG, et al. The trainer, the trainee and the surgeons' assistant: clinical outcomes following total hip replacement. J Bone Joint Surg Br. 2009;**91**(7):928-34.
13. Reidy MJ, Faulkner A, Shitole B, et al. Do trainee surgeons have an adverse effect on the outcome after total hip arthroplasty?: a ten-year review. Bone Joint J. 2016;**98-B**(3):301-6.
14. Beattie N, Maempel JF, Roberts S, et al. Surgery performed by supervised registrars does not adversely affect medium-term functional outcomes after total knee replacement. Ann R Coll Surg Engl. 2018;**100**(1):57-62.
15. Faulkner A, Reidy MJ, Davies PSE, et al. Balancing training and outcomes in total knee replacement: A ten-year review. Surgeon. 2018;**16**(4):227-31.
16. Bottomley N, Jones LD, Rout R, et al. A survival analysis of 1084 knees of the Oxford unicompartmental knee arthroplasty: a comparison between consultant and trainee surgeons. Bone Joint J. 2016;**98-B**(10 Supple B):22-7.
17. Higgins JPT, Green S, Cochrane Collaboration. Cochrane handbook for systematic reviews of interventions. Chichester, England ; Hoboken, NJ: Wiley-Blackwell; 2008. xxi, 649 p. p.
18. Stroup DF, Berlin JA, Morton SC, et al. Meta-analysis of observational studies in epidemiology: a proposal for reporting. Meta-analysis Of Observational Studies in Epidemiology (MOOSE) group. JAMA. 2000;**283**(15):2008-12.
19. Ouzzani M, Hammady H, Fedorowicz Z, et al. Rayyan-a web and mobile app for systematic reviews. Syst Rev. 2016;**5**(1):210.

For peer review only - http://bmjopen.bmj.com/site/about/guidelines.xhtml

20. Sterne JA, Hernan MA, Reeves BC, et al. ROBINS-I: a tool for assessing risk of bias in non-randomised studies of interventions. BMJ. 2016;**355**:i4919.
21. Atkins D, Best D, Briss PA, et al. Grading quality of evidence and strength of recommendations. BMJ. 2004;**328**(7454):1490.
22. Mantel N, Haenszel W. Statistical aspects of the analysis of data from retrospective studies of disease. J Natl Cancer Inst. 1959;**22**(4):719-48.
23. Higgins JP, Thompson SG. Quantifying heterogeneity in a meta-analysis. Stat Med. 2002;**21**(11):1539-58.
24. Egger M, Davey Smith G, Schneider M, et al. Bias in meta-analysis detected by a simple, graphical test. BMJ. 1997;**315**(7109):629-34.
25. Sterne JA, Sutton AJ, Ioannidis JP, et al. Recommendations for examining and interpreting funnel plot asymmetry in meta-analyses of randomised controlled trials. BMJ. 2011;**343**:d4002.
26. Muller DA, Zingg PO, Dora C. Anterior minimally invasive approach for total hip replacement: five-year survivorship and learning curve. Hip Int. 2014;**24**(3):277-83.
27. Jain S, Magra M, Dube B, et al. Reverse hybrid total hip arthroplasty. Bone Joint J. 2018;**100-B**(8):1010-7.
28. Hasegawa Y, Amano T. Surgical skills training for primary total hip arthroplasty. Nagoya J Med Sci. 2015;**77**(1-2):51-7.
29. Hernigou P, Manicom O, Flouzat-Lachaniete CH, et al. Fifteen year outcome of the ceraver hermes posterior-stabilized total knee arthroplasty: safety of the procedure with experienced and inexperienced surgeons. Open Orthop J. 2009;**3**:36-9.
30. Marston RA, Cobb AG, Bentley G. Stanmore compared with Charnley total hip replacement. A prospective study of 413 arthroplasties. J Bone Joint Surg Br. 1996;**78**(2):178-84.
31. Storey R, Frampton C, Kieser D, et al. Does Orthopaedic Training Compromise the Outcome in Knee Joint Arthroplasty? J Surg Educ. 2018;**75**(5):1292-8.
32. Inglis T, Dalzell K, Hooper G, et al. Does orthopedic training compromise the outcome in total hip arthroplasty? J Surg Educ. 2013;**70**(1):76-80.
33. Sayers A, Crowther MJ, Judge A, et al. Determining the sample size required to establish whether a medical device is non-inferior to an external benchmark. BMJ Open. 2017;**7**(8):e015397.

For peer review only

For peer review only - http://bmjopen.bmj.com/site/about/guidelines.xhtml

Figure and Title Legends

Fig. 1 – Study flow diagram

Fig. 2 – Meta-analysis of net implant survival of THRs at five years according to the training status of the surgeon

Fig. 3 – Meta-analysis of the relative risk of revision of THRs at five and ten years according to the training status of the surgeon

Fig. 4 – Meta-analysis of net implant survival of TKRs at ten years according to the training status of the surgeon

Table 1 – Characteristics of included studies

Table 2 – GRADE Summary of Findings Table

Supplementary Table 3 – Risk of bias (ROBINS-I) assessment of methodological quality

For peer review only

Contributors

TF, AB, AS, and MW conceived and designed the study; TF and AA independently screened the articles and performed data extraction in duplicate; TF and AS were responsible for data analysis; all authors were responsible for interpreting the data; TF drafted the manuscript; AB, AA, AS, and MW revised the article critically for important intellectual content; all authors reviewed the final version of the manuscript and gave approval for submission for publication. The corresponding author attests that all listed authors meet authorship criteria and that no others meeting the criteria have been omitted.

Funding

This study was also supported by the National Institute for Health Research (NIHR) Biomedical Research Centre at the University Hospitals Bristol NHS Foundation Trust and the University of Bristol. TF was supported by a NIHR Academic Clinical Fellowship. AS was supported by an MRC strategic skills fellowship (MR/L01226X/1). The views expressed in this publication are those of the authors and not necessarily those of the NHS, the NIHR, or the Department of Health and Social Care. The NIHR had no role in the design and conduct of the study; the collection, management, analysis, and interpretation of the data; the preparation, review, or approval of the manuscript; or the decision to submit the manuscript for publication.

Competing Interests

None declared.

Patient consent for publication

Not required

Provenance and peer review

For peer review only

For peer review only - http://bmjopen.bmj.com/site/about/guidelines.xhtml

Not commissioned; externally peer reviewed

Data availability statement

All data relevant to the study are included in the article or as online supplementary material.

For peer review only

For peer review only - http://bmjopen.bmj.com/site/about/guidelines.xhtml

Table 1 - Characteristics of included studies

Source, Year	Country	Study period	Study design	Implant	Training status terminology (primary exposure)	Follow up (years)	Number of cases (trainee)	Implant brand (stem/cup if hip)	Sex (%female)	Mean age (SD or range)	Indication (%OA)	Supervision reported	Survival analysis	Revision rates reported	ROBINS-I overall risk of bias†
Hasegawa,[28] 2015	Japan	2006-10	PC	THR	Trainee vs. instructor	5	483 (259)	Multiple	-	61.3 (SD 11.6)	-	No	Yes	No	Serious
Jain,[27] 2018	UK	2005-12	RC	THR	Trainee vs. consultant	5, 10	1082 (348)	Corail/multiple	61.3	69.2 (21-94)	91	No	Yes (Add.)	Yes	Moderate
Muller,[26] 2013	Switzerland	2005-06	RC	THR	Junior vs. senior	5	130 (43)	Quadra-H /Versafit-CC	52	64 (SD 12.36)	86	No	Yes	Yes	Serious
Palan,[12] 2009	UK	1999-02	RC	THR	Trainee vs. consultant trainer	5	1501 (528)	Exeter/multiple	-	68.4 (21-94)	-	No	No	Yes	Moderate
Reidy,[13] 2016	UK	2003-04	RC	THR	Trainee vs. consultant	10	870 (286)	Multiple	60.5	69.5 (37-94)	94.8	Yes	Yes (no CIs)	Yes	Moderate
Faulkner,[15] 2017	UK	2003-04	RC	TKR	Trainee vs. consultant	5, 10	686 (236)	Multiple	-	69.9 (30-94)	93.1	No	Yes (Add.)	Yes	Moderate
Hernigou,[29] 2009	France	1990-95	RC	TKR	Young (<30) vs. senior	10, 15	250 (150)	Ceraver Hermes	69.7	73 (46-88)	-	No	Yes	No	Serious
Bottomley,[16] 2016	UK	1998-08	RC	UKR	Trainee vs. consultant	10	1084 (673)	Oxford	51.4	66.5 (SD 9.6)	100	Yes	Yes	Yes	Moderate

PC, prospective cohort; RC, retrospective cohort; Add., additional data provided by author; CIs, confidence intervals; SD, standard deviation; † see supplementary table 3 for full risk of bias assessment

For peer review only - http://bmjopen.bmj.com/site/about/guidelines.xhtml

Table 2 – GRADE Summary of Findings Table

Outcomes	Follow up (years)	Trainee revision/cases†, n	Consultant revisions/cases†, n	Net survival/relative risk (95% CI)	Participants (studies), n	Quality of Evidence	Comments
THR: net implant survival	5	650	1045	NS: Trainee 97.9% (96.6 to 99.2)<br>NS: Consultant 98.1% (97.1 to 99.2)	1695 (3)[26-28]	Very low	Serious ROB, indirectness, and imprecision
	10	348	734	NS: Trainee 98.1% (95.9 to 99.1)<br>NS: Consultant 96.7% (94.7 to 97.9)	1082 (1)[27]	Low	Serious indirectness and imprecision
THR: crude revision rate	5	13/919	29/1794	RR: 0.88 (0.46 to 1.70)	2713 (3)[12, 26, 27]	Very low	Serious ROB, indirectness, and imprecision
	10	13/634	40/1318	RR: 0.68 (0.37 to 1.26)	1952 (2)[13, 27]	Low	Serious indirectness and imprecision
TKR: net implant survival	5	236	450	NS: Trainee 97.9% (95.0 to 99.2)<br>NS: Consultant 95.4% (93.0 to 97.0)	686 (1)[15]	Low	Serious imprecision
	10	386	550	NS: Trainee 96.2% (94.0 to 98.4)<br>NS: Consultant 95.1% (93.0 to 97.2)	936 (2)[15, 29]	Very low	Serious inconsistency and imprecision
	15	150	100	NS: Trainee 91.0% (85.0 to 97.0)<br>NS: Consultant 92.0% (90.0 to 94.0)	250 (1)[29]	Very low	Serious inconsistency and very serious imprecision
TKR: crude revision rate	5	5/236	20/450	RR: 0.47 (0.18 to 1.25)	686 (1)[15]	Low	Serious imprecision
	10	8/236	26/450	RR: 0.58 (0.27 to 1.27)	686 (1)[15]	Low	Serious imprecision
	15	4/150	4/100	RR: 0.67 (0.17 to 2.60)	250 (1)[29]	Very low	Serious inconsistency and very serious imprecision
UKR: net implant survival	10	673	411	NS: Trainee 93.0% (90.3 to 95.7)<br>NS: Consultant 93.9% (90.2 to 97.6)	1084 (1)[16]	Low	Serious imprecision
UKR: crude revision rate	10	31/673	15/411	RR: 1.26 (0.69 to 2.31)	1084 (1)[16]	Low	Serious imprecision

GRADE, Grading of Recommendations Assessment, Development and Evaluation; CI, confidence interval; NS, net survival; RR, relative risk; †, number of revisions not reported for net implant survival; ROB, risk of bias

For peer review only - http://bmjopen.bmj.com/site/about/guidelines.xhtml

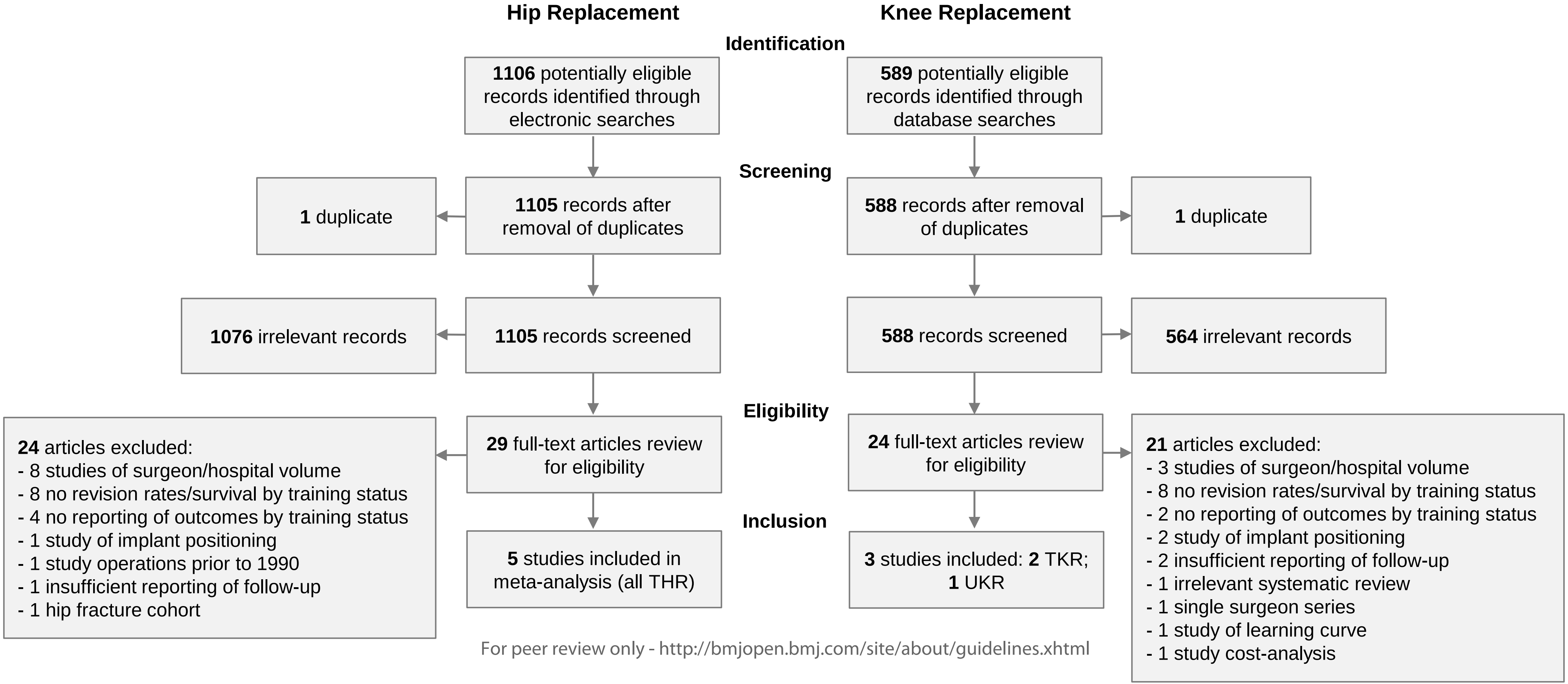
Hip Replacement
Knee Replacement
Identification
1106 potentially eligible records identified through electronic searches
589 potentially eligible records identified through database searches
Screening
1 duplicate
1105 records after removal of duplicates
588 records after removal of duplicates
1 duplicate
1076 irrelevant records
1105 records screened
588 records screened
564 irrelevant records
Eligibility
24 articles excluded:
- 8 studies of surgeon/hospital volume
- 8 no revision rates/survival by training status
- 4 no reporting of outcomes by training status
- 1 study of implant positioning
- 1 study operations prior to 1990
- 1 insufficient reporting of follow-up
- 1 hip fracture cohort
29 full-text articles review for eligibility
24 full-text articles review for eligibility
21 articles excluded:
- 3 studies of surgeon/hospital volume
- 8 no revision rates/survival by training status
- 2 no reporting of outcomes by training status
- 2 study of implant positioning
- 2 insufficient reporting of follow-up
- 1 irrelevant systematic review
- 1 single surgeon series
- 1 study of learning curve
- 1 study cost-analysis
Inclusion
5 studies included in meta-analysis (all THR)
3 studies included: 2 TKR; 1 UKR

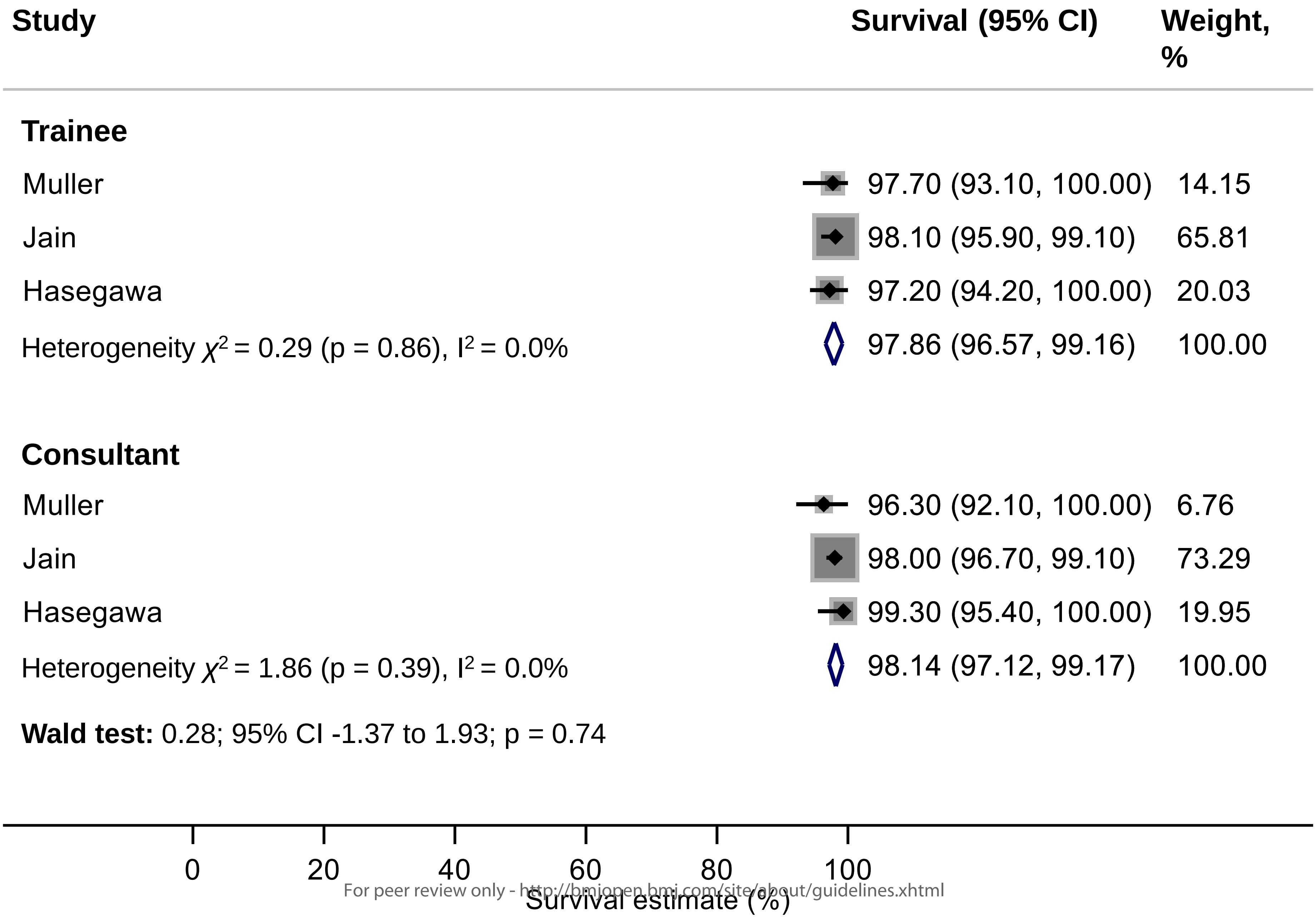
Study
Survival (95% CI)
Weight, %
Trainee
Muller
97.70 (93.10, 100.00)
14.15
Jain
98.10 (95.90, 99.10)
65.81
Hasegawa
97.20 (94.20, 100.00)
20.03
Heterogeneity χ^2 = 0.29 (p = 0.86), I^2 = 0.0%
97.86 (96.57, 99.16)
100.00
Consultant
Muller
96.30 (92.10, 100.00)
6.76
Jain
98.00 (96.70, 99.10)
73.29
Hasegawa
99.30 (95.40, 100.00)
19.95
Heterogeneity χ^2 = 1.86 (p = 0.39), I^2 = 0.0%
98.14 (97.12, 99.17)
100.00
Wald test: 0.28; 95% CI -1.37 to 1.93; p = 0.74
0
20
40
60
80
100
Survival estimate (%)

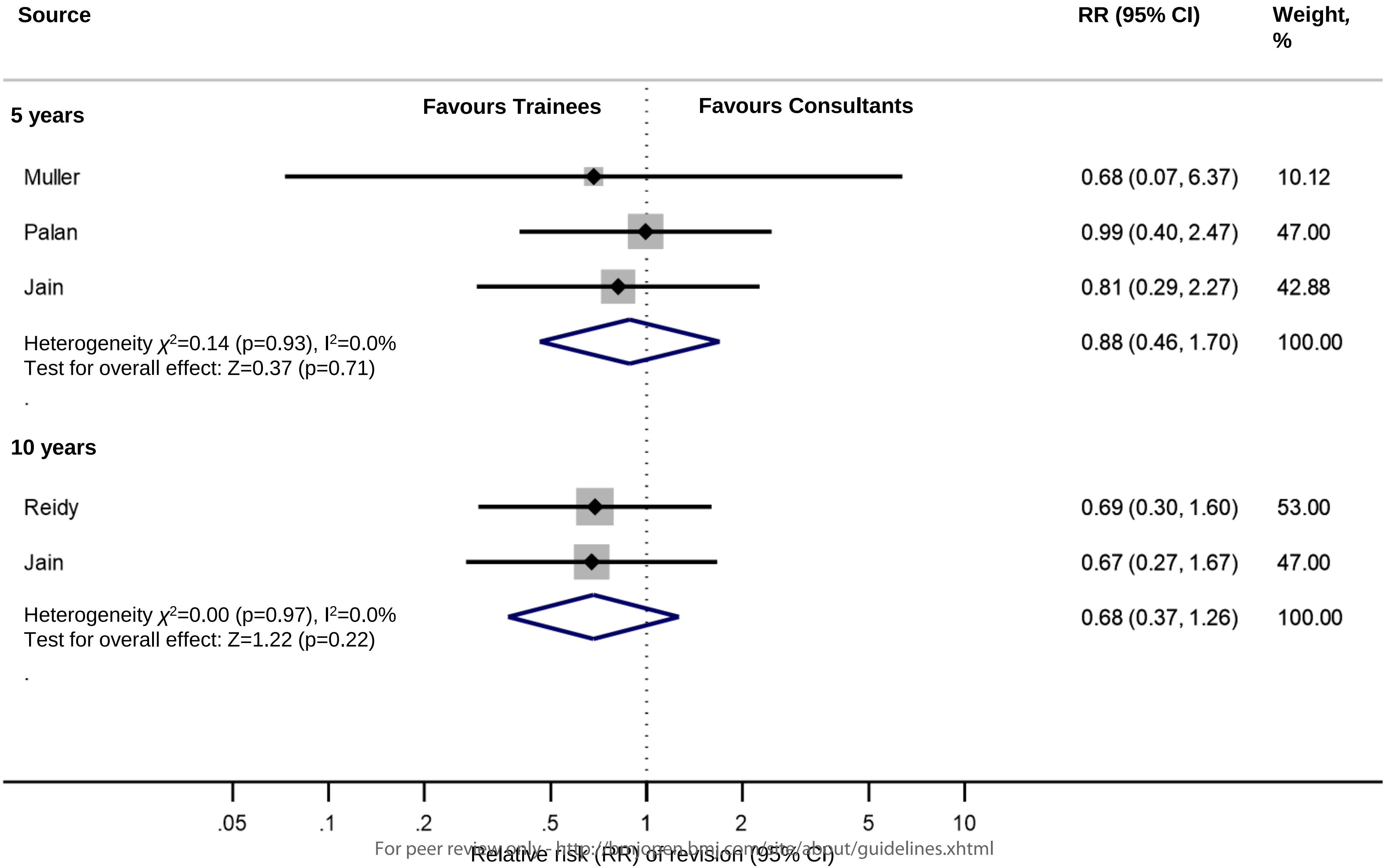

For peer review only - http://bmjopen.bmj.com/site/about/guidelines.xhtml

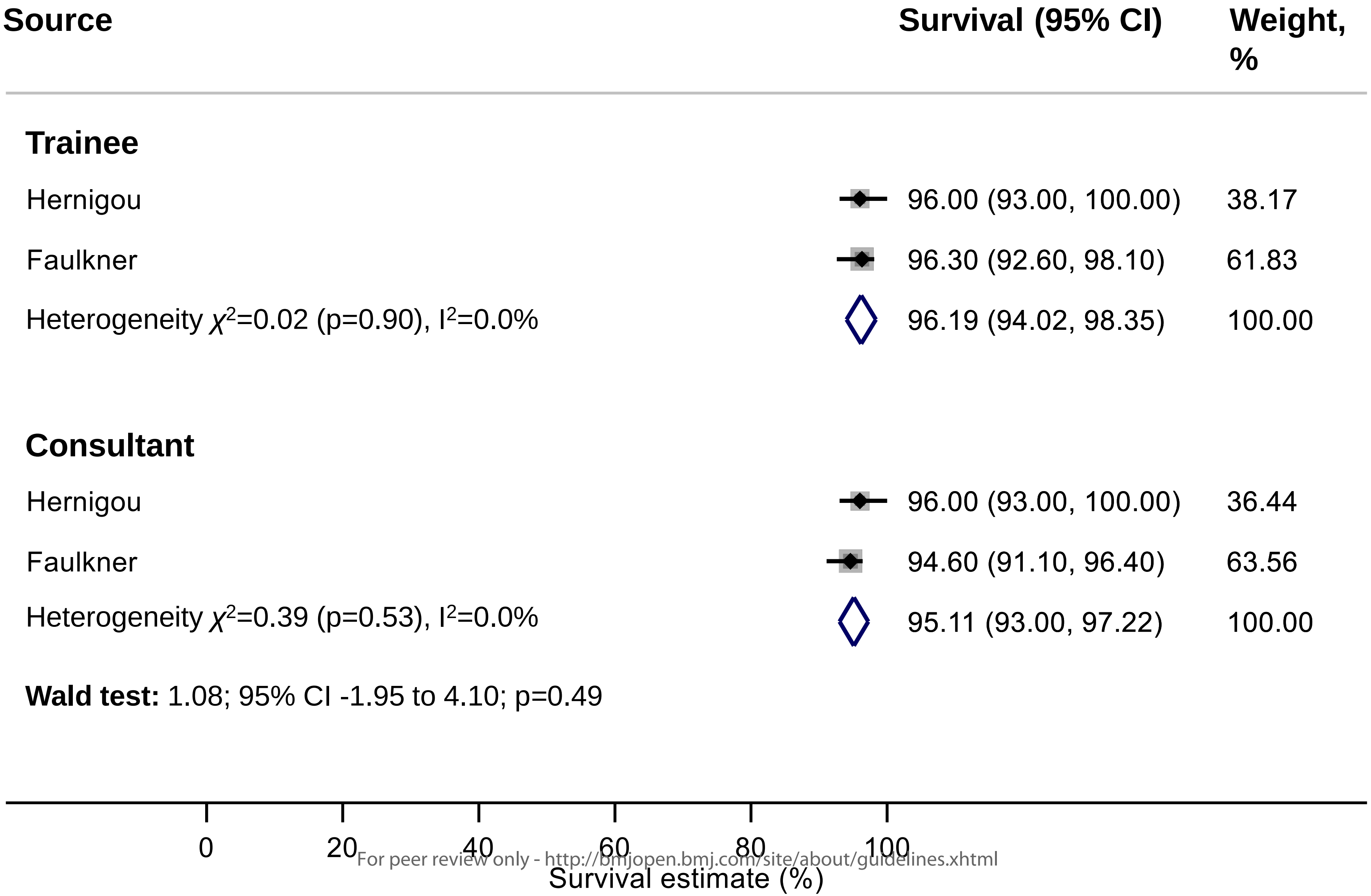

For peer review only - http://bmjopen.bmj.com/site/about/guidelines.xhtml

Online Supplementary Material

Contents:

Supplementary Methods

- Methods (page 2-3): Search strategy using Ovid (Medline + Embase)
- Methods (page 4): Reasons for Exclusion

Supplementary Table

- Table 3 (page 5): Risk of Bias (ROBINS-I) assessment of methodological quality

For peer review only

For peer review only - http://bmjopen.bmj.com/site/about/guidelines.xhtml

Methods: Search strategy using Ovid (Medline + Embase). Performed by TF & AS.

Hip Search

Hip replacement

Hip Prosthesis/ OR Arthroplasty, Replacement, Hip/ OR

(hip adj2 arthroplast$.mp) OR (hip adj2 replacement?.mp) OR (hip adj2 prosthes$.mp) OR

THA.mp OR THR.mp OR (TJR$.mp AND hip$.mp)

AND

Training

exp Education, Medical/ OR exp Inservice Training/ OR Clinical Competence/ OR

training.mp OR trainee.mp OR

experience.mp OR

junior.mp OR

senior$.mp OR

(surgeon adj2 grade).mp OR

consultant.mp OR attending?.mp OR registrar.mp OR SpR.mp OR StR.mp OR ST?.mp OR residen$.mp OR fellow$.mp OR intern.mp OR

(house adj2 officer).mp OR (foundation adj2 doctor).mp

AND

Survival

exp Prosthesis Failure/ OR exp Survival Analysis/ OR Reoperation/ OR

cox.mp OR proportional?hazard?.mp OR proportional hazard?.mp OR

cumulative?incidence?function.mp OR cumulative incidence function.mp OR CIF.mp OR

failure.mp OR

survival.mp OR survivor?ship.mp OR

revision?.mp OR

re?operation.mp OR re operation.mp OR

Kaplan?meier.mp OR Kaplan meier.mp OR KM.mp OR

product?limit?method.mp OR product limit method.mp

AND

Case-series

exp Cohort Studies/ OR Controlled Clinical Trials

follow?up.mp OR follow up.mp OR series.mp OR cohort.mp OR observational.mp OR longitudinal.mp OR prospective.mp OR retrospective.mp OR registry.mp OR registries.mp

For peer review only - http://bmjopen.bmj.com/site/about/guidelines.xhtml

Knee search

Knee replacement

Knee Prosthesis/ OR Arthroplasty, Replacement, Knee/ OR

(knee adj2 arthroplast$.mp) OR (knee adj2 replacement?.mp) OR (knee adj2 prosthes$.mp) OR

TKA.mp OR TKR.mp OR (TJR$.mp AND knee$.mp) OR

UKA.mp OR UKR.mp

AND

Training

exp Education, Medical/ OR exp Inservice Training/ OR Clinical Competence/ OR

training.mp OR trainee.mp OR

experience.mp OR

junior.mp OR

senior$.mp OR

(surgeon adj2 grade).mp OR

consultant.mp OR attending?.mp OR registrar.mp OR SpR.mp OR StR.mp OR ST?.mp OR residen$.mp OR fellow$.mp OR intern.mp OR

(house adj2 officer).mp OR (foundation adj2 doctor).mp

AND

Survival

exp Prosthesis Failure/ OR exp Survival Analysis/ OR Reoperation/ OR

cox.mp OR proportional?hazard?.mp OR proportional hazard?.mp OR

cumulative?incidence?function.mp OR cumulative incidence function.mp OR CIF.mp OR

failure.mp OR

survival.mp OR survivor?ship.mp OR

revision?.mp OR

re?operation.mp OR re operation.mp OR

Kaplan?meier.mp OR Kaplan meier.mp OR KM.mp OR

product?limit?method.mp OR product limit method.mp

AND

Case-series

exp Cohort Studies/ OR Controlled Clinical Trials

follow?up.mp OR follow up.mp OR series.mp OR cohort.mp OR observational.mp OR longitudinal.mp OR prospective.mp OR retrospective.mp OR registry.mp OR registries.mp

For peer review only - http://bmjopen.bmj.com/site/about/guidelines.xhtml

Reasons for Exclusion – Hip Papers	
**First author/Year of study**	**Reason for Exclusion**
De Vries, 2011	Principally a study of surgeon/hospital volume
Fender, 2003	Principally a study of surgeon/hospital volume
Hooper, 2009	Principally a study of surgeon/hospital volume
Johnsson, 1994	Principally a study of surgeon/hospital volume
Namba, 2012	Principally a study of surgeon/hospital volume
Ravi, 2014	Principally a study of surgeon/hospital volume
Canadian Arthroplasty Soc., 2013	Principally a study of surgeon/hospital volume
MacBride, 2010	Principally a study of surgeon/hospital volume
Enocson, 2009	No revision rates/survival analysis reported according to grade
Field, 2006	No revision rates/survival analysis reported according to grade
Leguerrand, 2018	No revision rates/survival analysis reported according to grade
Moran, 2004	No revision rates/survival analysis reported according to grade
Smith, 2018	No revision rates/survival analysis reported according to grade
Wilson, 2016	No revision rates/survival analysis reported according to grade
Wroblewski, 1998	No revision rates/survival analysis reported according to grade
Schoenfeld, 2013	No revision rates/survival analysis reported according to grade
Inglis, 2013	Insufficient reporting of follow-up
Marston, 1996	Study of operations performed prior to 1990
Khatod, 2014	No reporting of outcomes according to training status
Whitehouse, 2014	No reporting of outcomes according to training status
Williams, 2002	No reporting of outcomes according to training status
Zwartele, 2005	No reporting of outcomes according to training status
Kim, 2017	Principally a study of implant positioning
MacDonald, 2020	Hip fracture cohort; insufficient follow-up
N.B. Multiple reasons for some papers	

Reasons for Exclusion – Knee Papers	
**First author/Year of study**	**Reason for Exclusion**
Bini, 2013	Principally a study of surgeon/hospital volume
Namba, 2012	Principally a study of surgeon/hospital volume
Zambianchi, 2014	Principally a study of surgeon/hospital volume
Liddle, 2014	No revision rates/survival analysis reported according to grade
Beattie, 2016	No revision rates/survival analysis reported according to grade
Haughom, 2014	No revision rates/survival analysis reported according to grade
Khakha, 2015	No revision rates/survival analysis reported according to grade
Schoenfeld, 2013	No revision rates/survival analysis reported according to grade
Windisch, 2017	No revision rates/survival analysis reported according to grade
Wilson, 2016	No revision rates/survival analysis reported according to grade
Woolson, 2007	No revision rates/survival analysis reported according to grade
Atrey, 2014	No reporting of outcomes according to training status
Back, 2000	No reporting of outcomes according to training status
Gaillard, 2016	Principally a study of implant positioning
Mahaluxmivala, 2001	Principally a study of implant positioning
Storey, 2018	Insufficient reporting of follow-up
Theelen, 2018	Insufficient reporting of follow-up
Jasper, 2016	Irrelevant systematic review
Lacko, 2018	Single surgeon series
Matas-Diez, 2018	Principally a study of learning curve
Lavernia, 2000	Study of cost-analysis
N.B. Multiple reasons for some papers	

For peer review only - http://bmjopen.bmj.com/site/about/guidelines.xhtml

Supplementary Table 3: Risk of Bias (ROBINS-I) assessment

ROBINS-I	Bottomley, 2016	Faulkner, 2017	Hernigou, 2009	Hasegawa, 2015	Jain, 2018	Muller, 2013	Palan, 2009	Reidy, 2016
Bias due to confounding	⊕⊕	⊕	⊕⊕	⊕⊕	⊕	⊕⊕	⊕	⊕
Bias in selection of patients	⊕	⊕	⊕	⊕	⊕	⊕	⊕	⊕
Bias in classification of interventions	⊖	⊕	⊕	⊕⊕	⊕	⊕⊕	⊕	⊕
Bias due to deviations from interventions	⊕	⊕	⊕⊕	⊕⊕	⊕	⊕⊕	⊕	⊕
Bias due to missing data	⊖	⊕	⊕⊕	⊕⊕	⊕	⊕	⊖	⊕
Bias in measurement of outcome	⊖	⊕	⊖	⊕	⊖	⊖	⊖	⊕
Bias in selection of the reported result	⊖	⊖	⊖	⊕	⊖	⊕	⊖	⊖
Overall risk of Bias	⊕	⊕	⊕⊕	⊕⊕	⊕	⊕⊕	⊕	⊕

Key: ⊖ = low risk of bias; ⊕ = moderate risk of bias; ⊕⊕ = serious risk of bias; ⊕⊕⊕ = critical risk of bias

For peer review only

For peer review only - http://bmjopen.bmj.com/site/about/guidelines.xhtml

MOOSE Checklist for Meta-analyses of Observational Studies

Item No	Recommendation	Reported on Page No
Reporting of background should include		
1	Problem definition	4-5
2	Hypothesis statement	4-5
3	Description of study outcome(s)	4
4	Type of exposure or intervention used	4
5	Type of study designs used	6
6	Study population	6-7
Reporting of search strategy should include		
7	Qualifications of searchers (e.g., librarians and investigators)	Online supplementary methods
8	Search strategy, including time period included in the synthesis and key words	6 & online supplementary methods
9	Effort to include all available studies, including contact with authors	7, 9
10	Databases and registries searched	7
11	Search software used, name and version, including special features used (e.g., explosion)	7-8
12	Use of hand searching (e.g., reference lists of obtained articles)	6
13	List of citations located and those excluded, including justification	Online supplementary methods
14	Method of addressing articles published in languages other than English	6
15	Method of handling abstracts and unpublished studies	6-7
16	Description of any contact with authors	7, 10-12
Reporting of methods should include		
17	Description of relevance or appropriateness of studies assembled for assessing the hypothesis to be tested	6-8
18	Rationale for the selection and coding of data (e.g., sound clinical principles or convenience)	6-8
19	Documentation of how data were classified and coded (e.g., multiple raters, blinding and interrater reliability)	7-8
20	Assessment of confounding (e.g., comparability of cases and controls in studies where appropriate)	8
21	Assessment of study quality, including blinding of quality assessors, stratification or regression on possible predictors of study results	8
22	Assessment of heterogeneity	8
23	Description of statistical methods (e.g., complete description of fixed or random effects models, justification of whether the chosen models account for predictors of study results, dose-response models, or cumulative meta-analysis) in sufficient detail to be replicated	8-9
24	Provision of appropriate tables and graphics	Table 1-2, Figures 1-4
Reporting of results should include		
25	Graphic summarizing individual study estimates and overall estimate	Figures 2-4
26	Table giving descriptive information for each study included	Table 1-2
27	Results of sensitivity testing (e.g., subgroup analysis)	N/A, justification

For peer review only - http://bmjopen.bmj.com/site/about/guidelines.xhtml

		13-14
28	Indication of statistical uncertainty of findings	10-15

Item No	Recommendation	Reported on Page No
Reporting of discussion should include		
29	Quantitative assessment of bias (e.g., publication bias)	10
30	Justification for exclusion (e.g., exclusion of non-English language citations)	13-14
31	Assessment of quality of included studies	10, Table 2 & supplementary Table 3
Reporting of conclusions should include		
32	Consideration of alternative explanations for observed results	13-15
33	Generalization of the conclusions (i.e., appropriate for the data presented and within the domain of the literature review)	13-15
34	Guidelines for future research	15
35	Disclosure of funding source	20

From: Stroup DF, Berlin JA, Morton SC, et al, for the Meta-analysis Of Observational Studies in Epidemiology (MOOSE) Group. Meta-analysis of Observational Studies in Epidemiology. A Proposal for Reporting. *JAMA*. 2000;283(15):2008-2012. doi: 10.1001/jama.283.15.2008.

For peer review only

BMJ Open

The association between surgeon grade and implant survival following hip and knee replacement: a systematic review and meta-analysis

Journal:	*BMJ Open*
Manuscript ID	bmjopen-2020-047882.R2
Article Type:	Original research
Date Submitted by the Author:	11-Oct-2021
Complete List of Authors:	Fowler, Timothy; University of Bristol Medical School, Musculoskeletal Research Unit, Learning and Research Building, Southmead Hospital<br>Aquilina, Alex; University of Bristol Medical School, Musculoskeletal Research Unit, Learning and Research Building, Southmead Hospital<br>Blom, AW; University of Bristol Medical School, Musculoskeletal Research Unit, Learning and Research Building, Southmead Hospital; National Institute for Health Research, National Institute for Health Research Bristol Biomedical Research Centre, University Hospitals Bristol NHS Foundation Trust, University of Bristol<br>Sayers, Adrian; University of Bristol Medical School, Musculoskeletal Research Unit, Learning and Research Building, Southmead Hospital<br>Whitehouse, Michael; University of Bristol Medical School, Musculoskeletal Research Unit, Learning and Research Building, Southmead Hospital; National Institute for Health Research, National Institute for Health Research Bristol Biomedical Research Centre, University Hospitals Bristol NHS Foundation Trust, University of Bristol
**Primary Subject Heading**:	Surgery
Secondary Subject Heading:	Medical education and training, Health policy, Evidence based practice
Keywords:	Hip < ORTHOPAEDIC & TRAUMA SURGERY, Knee < ORTHOPAEDIC & TRAUMA SURGERY, Orthopaedic & trauma surgery < SURGERY, MEDICAL EDUCATION & TRAINING, Health policy < HEALTH SERVICES ADMINISTRATION & MANAGEMENT

SCHOLARONE™
Manuscripts

I, the Submitting Author has the right to grant and does grant on behalf of all authors of the Work (as defined in the below author licence), an exclusive licence and/or a non-exclusive licence for contributions from authors who are: i) UK Crown employees; ii) where BMJ has agreed a CC-BY licence shall apply, and/or iii) in accordance with the terms applicable for US Federal Government officers or employees acting as part of their official duties; on a worldwide, perpetual, irrevocable, royalty-free basis to BMJ Publishing Group Ltd ("BMJ") its licensees and where the relevant Journal is co-owned by BMJ to the co-owners of the Journal, to publish the Work in this journal and any other BMJ products and to exploit all rights, as set out in our licence.

The Submitting Author accepts and understands that any supply made under these terms is made by BMJ to the Submitting Author unless you are acting as an employee on behalf of your employer or a postgraduate student of an affiliated institution which is paying any applicable article publishing charge ("APC") for Open Access articles. Where the Submitting Author wishes to make the Work available on an Open Access basis (and intends to pay the relevant APC), the terms of reuse of such Open Access shall be governed by a Creative Commons licence – details of these licences and which Creative Commons licence will apply to this Work are set out in our licence referred to above.

Other than as permitted in any relevant BMJ Author's Self Archiving Policies, I confirm this Work has not been accepted for publication elsewhere, is not being considered for publication elsewhere and does not duplicate material already published. I confirm all authors consent to publication of this Work and authorise the granting of this licence.

For peer review only

The association between surgeon grade and implant survival following hip and knee replacement: a systematic review and meta-analysis

Authors

Timothy J. Fowler, MRCS[1]

Alex L. Aquilina, MRCS[1]

Ashley W. Blom, PhD[1,2]

*Adrian Sayers, PhD[1]

*Michael R. Whitehouse, PhD[1,2]

**Joint Senior Authors*

Author Affiliations

[1]Musculoskeletal Research Unit, Translational Health Sciences, Bristol Medical School, Southmead Hospital, Bristol, UK

[2]National Institute for Health Research Bristol Biomedical Research Centre, University Hospitals Bristol NHS Foundation Trust, University of Bristol, Bristol, UK

Corresponding Author

Mr Timothy J Fowler, BMBCh (Oxon) MA MRCS

Address: Musculoskeletal Research Unit, Translational Health Sciences, Bristol Medical School, Southmead Hospital, Bristol, UK, BS10 5NB

Email: t.j.fowler@bristol.ac.uk; *Phone:* +44 (0)117 414 7865

Word count

3471

For peer review only

Abstract

Objective: To investigate the association between surgeon grade (trainee vs. consultant) and implant survival following primary hip and knee replacement.

Design: A systematic review and meta-analysis of observational studies.

Data sources: MEDLINE® and Embase® from inception until October 6th, 2021.

Setting: Units performing primary hip and/or knee replacements since 1990.

Participants: Adult patients undergoing either a primary hip or knee replacement, predominantly for osteoarthritis.

Intervention: Whether the surgeon recorded as performing the procedure was a trainee or not.

Primary and secondary outcome measures: The primary outcome was net implant survival reported as a Kaplan-Meir survival estimate. The secondary outcome was crude revision rate. Both outcomes were reported according to surgeon grade.

Results: Nine cohort studies capturing 4066 total hip replacements (THRs), 936 total knee replacements (TKRs), and 1357 unicompartmental knee replacements (UKRs) were included (five THR studies, two TKR studies, and two UKR studies). The pooled net implant survival estimates for THRs at five years were 97.9% (95% CI 96.6 to 99.2) for trainees and 98.1% (95% CI 97.1 to 99.2) for consultants. The relative risk of revision of THRs at five and ten years was 0.88 (95% CI 0.46 to 1.70) and 0.68 (95% CI 0.37 to 1.26), respectively. For TKRs, the net implant survival estimates at ten years were 96.2% (95% CI 94.0 to 98.4) for trainees and 95.1% (95% CI 93.0 to 97.2) for consultants. We report a narrative summary of UKR outcomes.

Conclusions: There is no strong evidence in the existing literature that trainee surgeons have worse outcomes compared to consultants, in terms of the net survival or crude revision rate of hip and knee replacements at five to ten years follow up. These findings are limited by the quality of the existing published data and are applicable to countries with established orthopaedic training programmes.

For peer review only

Article Summary

Strengths and limitations of this study

- To our knowledge, this is the first meta-analysis of the association between surgeon grade and implant survival following hip and knee replacement.
- We performed a comprehensive systematic review according to current best practice guidelines.
- The findings of this review are limited by the strength of the existing published data from a relatively small number of predominantly retrospective observational studies.

For peer review only

Introduction

Hip and knee replacements are effective surgical interventions for the treatment of end stage degenerative conditions of the hip and knee.[1, 2] More than 200,000 are performed per year in the United Kingdom alone.[3] These procedures are performed by surgeons at various stages in their training, with varying levels of senior supervision. Contemporary training practices must ensure a balance between protecting development opportunities for the next generation of surgeons, while limiting the exposure of patients to unnecessary risk during the training process.

Implant survival, which is determined by the absence of revision surgery, is an important and commonly used measure of surgical performance.[4, 5] Net survival estimates are calculated using statistical methods of survival analysis (e.g. Kaplan-Meier analysis), which look at time to a defined failure 'event' (e.g. revision) and account for censored data that arise due to incomplete follow up, or death.[6] Another commonly reported metric is crude revision rate, which is defined as the observed number of failure events in a specified period of time.

The survival of hip and knee replacements according to surgeon grade is poorly understood. Higher rates of complications and longer operative times have been identified in orthopaedic procedures performed by trainees.[7, 8] Radiographic studies comparing trainee and consultant joint replacement have identified differences in acetabular anteversion,[9] hip centre of rotation,[10] and various measures of knee replacement component positioning.[11] However, the relative impact of these findings on implant survival has not been established. It has been suggested that when trainees are appropriately supervised, they can obtain comparable functional outcomes and implant survivorship to their consultant colleagues when performing total hip replacement (THR),[12-14] total knee replacement (TKR)[15] and unicompartmental knee replacement (UKR).[16]

The aim of this study was to conduct a systematic review and meta-analysis using the existing literature on the association between surgeon grade (trainee vs. consultant) and implant survival outcomes in hip and knee replacement surgery. We aimed to answer the question – do trainees

For peer review only - http://bmjopen.bmj.com/site/about/guidelines.xhtml

achieve equivalent implant survival outcomes to consultants when performing primary hip and knee replacement?

Methods

This review was conducted using methods described in the Cochrane Handbook for Systematic Reviews of Interventions, with reporting in accordance with the Meta-analyses Of Observational Studies in Epidemiology (MOOSE) checklist.[17, 18] The study was registered with the PROSPERO database at inception (CRD42019150494).

Data sources and search strategy

We searched for cohort studies reporting implant survival estimates and/or revision rates of hip or knee replacements, according to surgeon grade. Separate searches were performed for hips and knees. We conducted searches of MEDLINE® and Embase™ from inception until October 6th, 2021. Searches used keywords and MeSH (Medical Subject Headings) terms relating to hip and knee replacement, implant survival, revision surgery and surgeon grade (see online supplementary methods). There were no language restrictions. Titles and abstracts of potentially relevant non-English language citations were translated. We manually screened the bibliographies of full text articles and used Web of Science™ citation tracking to identify additional relevant studies.

Eligibility criteria

We included studies if they involved predominantly unselected adult patients (≥ 18 years old) undergoing primary hip or knee replacement (including THR, TKR, UKR and hip resurfacing), predominantly for the treatment of osteoarthritis. Included articles needed to report the primary and/or secondary outcome measure for two different groups of surgeons defined according to their grade (e.g. trainee vs. consultant). We defined a minimum follow up of five years and articles that did not clearly define the length of follow up were excluded. For example, we excluded studies reporting the revision rate 'per 100 component years', as these did not explicitly define the length of follow up. We

For peer review only - http://bmjopen.bmj.com/site/about/guidelines.xhtml

excluded studies in which the index operation was performed prior to 1990; thereby, including studies that are representative of contemporary training practices, but also allowing for inclusion of studies reporting in excess of 30 years of follow up (see online supplementary methods).

Primary exposure

The primary exposure was whether the surgeon recorded as performing the procedure was a trainee or not. Surgeon grade is a measure of the designated level of surgical experience and seniority, which we considered to be a binary variable: either 'trainee', or 'consultant'. Consultant surgeons have completed their formal training in orthopaedic surgery and have been appointed to a senior position in which they can practice independently and supervise trainee surgeons. The term 'consultant' is used synonymously with 'attending surgeon' in many healthcare settings including the United States. Additional terms used to describe this variable were deemed eligible during screening (e.g. Trainee: registrar; resident; junior/young surgeon; fellow. Consultant: attending; senior surgeon; trainer).

Outcome measures

The primary outcome was net implant survival, reported as a Kaplan-Meir survival estimate. The secondary outcome measure was crude revision rate, which was defined as the observed number of revisions in a specified period of time.

Screening and data extraction

Two authors (TF and AA) independently screened all titles and abstracts of journal articles using Rayyan (Rayyan QCRI, Doha).[19] Studies were initially screened for relevance according to information contained within the title and abstract. Cases of disagreement were resolved through re-review and consensus. Full texts of potentially relevant studies were reviewed in detail and disagreements on final inclusion were resolved through discussion with a senior author (MW). Specific indications for exclusion were documented following full text review (figure 1 and online supplementary methods).

For peer review only - http://bmjopen.bmj.com/site/about/guidelines.xhtml

Data were extracted in duplicate using a standardised proforma. We recorded data on the following: healthcare setting, study period, implant type, age, sex, indication, level of supervision, crude revision rate, and net implant survival estimates (including confidence intervals [CI]). Life tables were reviewed, and estimates were extracted for all available five-year intervals of follow up. Discrepancies in data collection were resolved through re-review and consensus. Where survival estimates, CIs and revision rates were incompletely reported, we contacted corresponding authors to request missing data.

For peer review only

Risk of bias and quality of evidence assessment

The risk of bias was assessed using the Cochrane ROBINS-I tool for the risk of bias in non-randomised cohort studies.[20] We assessed the quality of evidence for each outcome using the Grading of Recommendations Assessment, Development and Evaluation (GRADE) approach, which considers the imprecision, inconsistency, indirectness, and risk of bias in a body of evidence.[21]

Statistical analysis

Statistical analysis was performed using Stata (Version SE 15.1; StataCorp, Texas). For the primary outcome measure of net implant survival, we performed separate meta-analyses for each implant type, by surgeon grade and length of follow up. We pooled survival estimates, assuming that survivorship approximated risk, with fixed effects meta-analysis weighting each study on the overall pooled estimate according to its standard error, which was calculated from published CIs; an established method for the meta-analysis of implant survival estimates described by Evans *et al*.[4, 5] The effect size (survival) for trainees and consultants, was compared using a Wald test. For the secondary outcome measure, we derived and meta-analysed the relative risk (RR) of revision for each implant type by surgeon grade and length of follow up. We used a fixed effects model using the Mantel-Haenszel method.[22] Heterogeneity was assessed with chi-squared tests, with I^2 used to quantify inconsistency.[23] Publication bias was assessed by inspecting funnel plot symmetry.[24]

Patient and public involvement

For peer review only - http://bmjopen.bmj.com/site/about/guidelines.xhtml

There was no direct patient or public involvement in the design or conduct of this review.

For peer review only

Results

Separate searches for hip and knee replacements identified 1178 and 634 articles, respectively. After removal of duplicates and abstract screening, 30 hip papers and 27 knee papers remained. Through review of full text articles, we identified five hip and four knee studies eligible for inclusion. This process of review is summarised as a flow diagram in figure 1 and the characteristics of included studies are summarised in table 1. Six studies were conducted in the UK, with the remaining three studies originating from France, Switzerland, and Japan.

Risk of bias assessment

Supplementary table 1 provides a summary of the ROBINS-I assessment, which indicates a moderate to severe risk of bias in all studies. Funnel plot asymmetry and statistical tests for funnel plot asymmetry as a means of assessing publication bias were not applicable due to the small number of studies.[25]

Hip replacement

The five included hip studies represent 1464 THRs performed by trainees and 2602 THRs performed by consultants, with follow up ranging from five to ten years. Four studies were retrospective cohort studies;[12, 13, 26, 27] one was a non-randomised prospective cohort study.[28] No articles on hip resurfacing met the inclusion criteria. One author provided additional unpublished data in the form of net survival estimates.[27] Reidy *et al* reported survival estimates, but no CIs.[13] Net survival estimates and corresponding CIs were thus extracted from three studies at five years and one study at ten years. Crude revision rates were reported in three studies at five years and two studies at ten years.

For peer review only - http://bmjopen.bmj.com/site/about/guidelines.xhtml

Primary outcome: Net implant survival (THR)

Meta-analysis showed net survivorship of 97.9% (95% CI 96.6 to 99.2) at five years for THRs performed by trainees, compared to 98.1% (95% CI 97.1 to 99.2) for THRs performed by consultants (figure 2). There was no strong evidence of an association between surgeon grade and net implant survival at this interval of follow up (Wald test: p=0.74).

Meta-analysis was not possible for the ten-year data given the availability of only one study for this timepoint. In a cohort of 1082 reverse hybrid THRs, Jain *et al* demonstrated overall 97.2% implant survival at ten years. Additional data provided by the author indicate that they found no evidence of a difference in implant survival according to surgeon grade (Trainee: 98.1%; 95% CI 95.9 to 99.1; Consultant: 96.7%; 95% CI 94.7 to 97.9).[27]

Secondary outcome: Crude revision rate (THR)

Meta-analysis showed no strong evidence of an association between surgeon grade and the crude revision rate at five, or ten years. The RR of revision at five and ten years was 0.88 (95% CI 0.46 to 1.70) and 0.68 (95% CI 0.37 to 1.26), respectively (figure 3).

Knee replacement

The four knee studies represent 1177 knee replacements (TKR n=386; UKR n=791) performed by trainees and 1116 knee replacements (TKR n=550; UKR n=566) performed by consultants, with follow up ranging from five to fifteen years. All four were retrospective cohort studies.[15, 16, 29, 30] Two studies reported on TKRs,[15, 29]and two studies reported on UKRs.[16, 30]

With regards to the two TKR studies, Faulkner *et al* provided additional unpublished survival data from which we calculated corresponding CIs for their published survival estimates.[15] Net survival estimates and CIs were thus extracted from both TKR studies at ten years, which permitted meta-analysis of this primary outcome measure. Crude revision rates were only available from one TKR study at each five-year interval of follow up.

For peer review only - http://bmjopen.bmj.com/site/about/guidelines.xhtml

With regards to the two UKR papers, net survival estimates were only available from one study.[16] Crude revision rates were available from one study at five years and one study at ten years.[16, 30] Meta-analysis was not feasible, thus we provide a narrative summary of UKR outcomes.

Primary outcome: Net implant survival (TKR)

Meta-analysis showed net survivorship of 96.2% (95% CI 94.0 to 98.4) at ten years for TKRs performed by trainees, compared to 95.1% (95% CI 93.0 to 97.2) for TKRs performed by consultants (figure 4). There was no strong evidence of an association between surgeon grade and net implant survival at this interval of follow up (Wald test: p=0.49).

Secondary outcome: Crude revision rate (TKR)

Two studies reported crude revision rates according to surgeon grade; however, with data from only one study available at each interval of follow up, meta-analysis was not feasible. Instead, we provide a narrative summary. Faulkner *et al* provided additional unpublished data, which indicated crude revision rates at five years for trainees and consultants of 2.1% and 4.4%, respectively.[15] This rises to 3.4% (trainees) and 5.8% (consultants) at ten years. These data represent a RR of revision of 0.49 (95% CI 0.19 to 1.28) at five years and 0.60 (95% CI 0.28 to 1.31) at ten years. Hernigou published crude revision rates at 15 years of 2.7% for junior surgeons and 4.0% for senior surgeons, which represents a RR of revision of 0.68 (95% CI 0.17 to 2.64).[29]

Unicompartmental knee replacement (UKR)

Both UKR studies were conducted in the same centre but capture separate cohorts of patients.[16, 30] Bottomley *et al* conducted a retrospective cohort study of 1084 consecutive UKRs performed between 1998 and 2008. They demonstrated that consultants and trainees had cumulative 9-year survival estimates of 93.9% and 93.0%, respectively. They found no strong evidence of a difference in implant survival between the groups (log rank: p=0.30).[16] These data represent crude revision rates at 10 years

For peer review only - http://bmjopen.bmj.com/site/about/guidelines.xhtml

of 4.6% and 3.6% for trainees and consultants, respectively (RR 1.26; 95% CI 0.69 to 2.31). Trainees were supervised by a scrubbed consultant in 48% of cases.

Alvand *et al* reported a series of 273 UKRs performed between 2009 and 2015. They did not report net survival estimates according to surgeon grade. However, they reported crude revision rates at 5 years of 0.8% and 2.6% for trainees and consultants, respectively. These data represent a RR of revision of 0.33 (95% CI 0.04 to 2.90). Trainees were supervised by a scrubbed consultant in 100% of cases.

Assessment of the quality of evidence

The GRADE assessment of the quality of evidence for each outcome indicates a low, or very low quality of evidence for all outcomes (table 2).

For peer review only - http://bmjopen.bmj.com/site/about/guidelines.xhtml

Discussion

The results of this study suggest that, in the context of contemporary practice, trainees do not achieve worse hip and knee replacement survival outcomes compared to their consultant colleagues at five to ten years follow up. We found no strong evidence of an association between surgeon grade and the net survival of THRs at five years (trainees: 97.9% vs. consultants: 98.1%). There was no association between surgeon grade and the crude revision rate of THRs at either five, or ten years follow up. Furthermore, we found no strong evidence of an association between surgeon grade and the net survival of TKRs at ten years (trainees: 96.2% vs consultants: 95.1%). Our narrative summary of two studies, highlights that there is no evidence in the existing literature of an association between trainee performed UKR and an increased risk of revision.

Strengths and limitations

This review has a number of strengths. We conducted a comprehensive systematic review with an exhaustive search according to current best practice guidelines and published the protocol for the methodology at inception. However, the data captured by this review have several limitations, which we have attempted to address through quality of evidence assessment and risk of bias analysis. The GRADE assessment indicates a low to very low quality of evidence for each outcome. Furthermore, the ROBINS-I assessment indicates a moderate to severe risk of bias in the included studies. These findings are generally consistent with the predominantly retrospective design of the included studies. The conclusions of this review are therefore limited by the strength and quality of the existing published data, which originate from a relatively small number of observational studies.

Meta-analysis of the primary outcome measure was only possible at five and ten years for THRs and ten years for TKRs, which limits the generalisability of our findings to these short and medium-term intervals of follow up. Therefore, this review does not capture any differences in early failure rates that might exist between trainee and consultant cohorts before five years. The included studies

For peer review only - http://bmjopen.bmj.com/site/about/guidelines.xhtml

originated from the UK, France, Switzerland, and Japan, which limits the generalisability of the findings to countries with established orthopaedic training programmes.

Formal orthopaedic training is a long process (lasting up to ten years in some countries); therefore, individual trainees have varying levels of experience, which are not captured by the binary variables used in this study, or in the existing literature. The included studies did not provide sufficient data to perform meaningful adjustment or sensitivity analysis according to specific training grade, or the level of senior supervision. Furthermore, our study captures cases performed between 1990 and 2015 (table 1) and we were unable to adjust for variations in training practice (such as the level of senior supervision) that may have occurred over this 25-year period.

Implant survival is a key determinant of good outcome in joint replacement surgery and is the sole variable considered in the current benchmarking strategies for the assessment of implant components. However, this review did not consider other factors that may be important when evaluating surgical outcomes, such as patient reported outcome measures, or complications other than failure, which have previously been found to occur in higher rates when joint replacements are performed by less experienced surgeons.[7, 8]

Published literature did not consistently report age, sex, comorbidities, implant design, or the level of senior supervision; making it very difficult to adjust for these variables. Methods of categorising the procedural complexity of a hip or knee replacement are not widely used in the orthopaedic literature and were not reported by any of the studies included in this review. Therefore, it was not possible to adjust for this factor. It is reasonable to suggest that the predominantly superior survival outcomes observed in the trainee cohorts are a product of patient selection and close senior supervision, with good trainers selecting appropriately complex cases for their trainees.

Comparison with other studies

A single study was excluded because the THRs under follow up were performed prior to 1990;[31] thus not considered representative of contemporary training practices. The authors of this ten-year study of

For peer review only - http://bmjopen.bmj.com/site/about/guidelines.xhtml

413 THRs reported a significantly higher rate of revision for trainees, with 15 of 16 revised hips performed by trainees. Inclusion of this study in our meta-analysis of ten-year THR crude revision rates increases the RR of revision to 1.12 (95% CI 0.66 to 1.92), in favour of THRs performed by consultants. One explanation for this is that the model of training in the UK at the time differed, with trainees more often operating without appropriate senior supervision.

Our findings are consistent with those of the New Zealand Joint Registry.[32, 33] In a cohort of 35 415 THRs, of which 4049 were performed by trainees, the authors reported no significant difference in the revision rate between surgeon groups.[33] In a further cohort of 79 671 TKRs and 8854 UKRs, of which approximately 10% were performed by trainees, they reported no significant difference in the revision rates of knee replacements performed by trainees and consultants.[32] These studies were not included in this meta-analysis because the authors did not report net survival estimates and revision rates were reported 'per 100 component years', rather than for clearly defined periods of follow up, which cannot be calculated from the data presented.

Implications

There is a delicate balance between ensuring optimal outcomes for patients and the necessity to train the next generation of surgeons. Reidy and Faulkner suggest that the availability of surgeon level registry data as a means of benchmarking performance, may lead to a desire to avoid perceived poor performance and thus a reluctance among consultants to let trainees operate.[13, 15] However, the findings of this review are encouraging and support the notion that in the context of contemporary practice, in countries with established and regulated orthopaedic training programmes, trainees can achieve implant survival outcomes equivalent to their consultant colleagues. The senior supervision of trainees was inconsistently reported in the studies included in this review but is likely to play an important role in the successful outcome of trainee performed hip and knee replacements.

An adequately powered non-inferiority RCT with ten years follow up assuming an acceptable revision rate of 5% and a 1% absolute non-inferiority delta ($\alpha = 0.05$; power = 0.80; 1:1 allocation ratio),

For peer review only - http://bmjopen.bmj.com/site/about/guidelines.xhtml

would require a sample size of 6400 patients.[34] However, factors inherent to the training process, such as variation amongst trainees, the need for case selection according to complexity and varying levels of supervision based on a trainee's experience, may preclude an inclusive and therefore generalisable RCT. Further investigation should focus on the associations between senior supervision, specific surgeon training grade, and the risk of revision following trainee-performed hip and knee replacements. Future work should also investigate the risk of early revision and the specific indications for revision following trainee-performed procedures. The analysis of unselected patient data recorded in a mandatory national joint replacement registry would be an appropriate means of further investigation.

Conclusions

In conclusion, there is no strong evidence in the existing literature that trainee surgeons have worse outcomes than their consultant surgeon colleagues, in terms of the net survival, or crude revision rate of hip and knee replacements at five to ten years follow up. This may mean that there is no difference, or that appropriate case mix selection and supervision of trainees is currently employed and is safe to continue. Our results are concordant with published registry data,[32, 33] and represent the best available evidence, but are limited by the quality of the existing published studies.

For peer review only

Ethical Approval Statement

Not applicable

References

1. Ferguson RJ, Palmer AJ, Taylor A, et al. Hip replacement. Lancet. 2018;**392**(10158):1662-71.

2. Price AJ, Alvand A, Troelsen A, et al. Knee replacement. Lancet. 2018;**392**(10158):1672-82.

3. NJR Centre. The National Joint Registry for England, Wales, Northern Ireland and the Isle of Man: 17th Annual Report, 2020.

4. Evans JT, Evans JP, Walker RW, et al. How long does a hip replacement last? A systematic review and meta-analysis of case series and national registry reports with more than 15 years of follow-up. Lancet. 2019;**393**(10172):647-54.

5. Evans JT, Walker RW, Evans JP, et al. How long does a knee replacement last? A systematic review and meta-analysis of case series and national registry reports with more than 15 years of follow-up. Lancet. 2019;**393**(10172):655-63.

6. Sayers A, Evans JT, Whitehouse MR, et al. Are competing risks models appropriate to describe implant failure? Acta Orthop. 2018;**89**(3):256-8.

7. Hedlundh U, Ahnfelt L, Hybbinette CH, et al. Surgical experience related to dislocations after total hip arthroplasty. J Bone Joint Surg Br. 1996;**78**(2):206-9.

8. Schoenfeld AJ, Serrano JA, Waterman BR, et al. The impact of resident involvement on post-operative morbidity and mortality following orthopaedic procedures: a study of 43,343 cases. Arch Orthop Trauma Surg. 2013;**133**(11):1483-91.

9. Moran M, Yap SL, Walmsley P, et al. Clinical and radiologic outcome of total hip arthroplasty performed by trainee compared with consultant orthopedic surgeons. J Arthroplasty. 2004;**19**(7):853-7.

10. Kim SC, Lim YW, Kwon SY, et al. Level of surgical experience is associated with change in hip center of rotation following cementless total hip arthroplasty: A radiographic assessment. PLoS One. 2017;**12**(5):e0178300.

For peer review only

For peer review only - http://bmjopen.bmj.com/site/about/guidelines.xhtml

11. Kazarian GS, Lawrie CM, Barrack TN, et al. The Impact of Surgeon Volume and Training Status on Implant Alignment in Total Knee Arthroplasty. J Bone Joint Surg Am. 2019;**101**(19):1713-23.

12. Palan J, Gulati A, Andrew JG, et al. The trainer, the trainee and the surgeons' assistant: clinical outcomes following total hip replacement. J Bone Joint Surg Br. 2009;**91**(7):928-34.

13. Reidy MJ, Faulkner A, Shitole B, et al. Do trainee surgeons have an adverse effect on the outcome after total hip arthroplasty?: a ten-year review. Bone Joint J. 2016;**98-B**(3):301-6.

14. Beattie N, Maempel JF, Roberts S, et al. Surgery performed by supervised registrars does not adversely affect medium-term functional outcomes after total knee replacement. Ann R Coll Surg Engl. 2018;**100**(1):57-62.

15. Faulkner A, Reidy MJ, Davies PSE, et al. Balancing training and outcomes in total knee replacement: A ten-year review. Surgeon. 2018;**16**(4):227-31.

16. Bottomley N, Jones LD, Rout R, et al. A survival analysis of 1084 knees of the Oxford unicompartmental knee arthroplasty: a comparison between consultant and trainee surgeons. Bone Joint J. 2016;**98-B**(10 Supple B):22-7.

17. Higgins JPT, Green S, Cochrane Collaboration. Cochrane handbook for systematic reviews of interventions. Chichester, England ; Hoboken, NJ: Wiley-Blackwell; 2008. xxi, 649 p. p.

18. Stroup DF, Berlin JA, Morton SC, et al. Meta-analysis of observational studies in epidemiology: a proposal for reporting. Meta-analysis Of Observational Studies in Epidemiology (MOOSE) group. JAMA. 2000;**283**(15):2008-12.

19. Ouzzani M, Hammady H, Fedorowicz Z, et al. Rayyan-a web and mobile app for systematic reviews. Syst Rev. 2016;**5**(1):210.

20. Sterne JA, Hernan MA, Reeves BC, et al. ROBINS-I: a tool for assessing risk of bias in non-randomised studies of interventions. BMJ. 2016;**355**:i4919.

21. Atkins D, Best D, Briss PA, et al. Grading quality of evidence and strength of recommendations. BMJ. 2004;**328**(7454):1490.

22. Mantel N, Haenszel W. Statistical aspects of the analysis of data from retrospective studies of disease. J Natl Cancer Inst. 1959;**22**(4):719-48.

For peer review only

23. Higgins JP, Thompson SG. Quantifying heterogeneity in a meta-analysis. Stat Med. 2002;**21**(11):1539-58.

24. Egger M, Davey Smith G, Schneider M, et al. Bias in meta-analysis detected by a simple, graphical test. BMJ. 1997;**315**(7109):629-34.

25. Sterne JA, Sutton AJ, Ioannidis JP, et al. Recommendations for examining and interpreting funnel plot asymmetry in meta-analyses of randomised controlled trials. BMJ. 2011;**343**:d4002.

26. Muller DA, Zingg PO, Dora C. Anterior minimally invasive approach for total hip replacement: five-year survivorship and learning curve. Hip Int. 2014;**24**(3):277-83.

27. Jain S, Magra M, Dube B, et al. Reverse hybrid total hip arthroplasty. Bone Joint J. 2018;**100-B**(8):1010-7.

28. Hasegawa Y, Amano T. Surgical skills training for primary total hip arthroplasty. Nagoya J Med Sci. 2015;**77**(1-2):51-7.

29. Hernigou P, Manicom O, Flouzat-Lachaniete CH, et al. Fifteen year outcome of the ceraver hermes posterior-stabilized total knee arthroplasty: safety of the procedure with experienced and inexperienced surgeons. Open Orthop J. 2009;**3**:36-9.

30. Alvand A, Wilson HA, Sabah SA, et al. New instrumentation system for cementless mobile-bearing unicompartmental knee arthroplasty improves surgical performance particularly for trainees. Knee. 2021;**31**:46-53.

31. Marston RA, Cobb AG, Bentley G. Stanmore compared with Charnley total hip replacement. A prospective study of 413 arthroplasties. J Bone Joint Surg Br. 1996;**78**(2):178-84.

32. Storey R, Frampton C, Kieser D, et al. Does Orthopaedic Training Compromise the Outcome in Knee Joint Arthroplasty? J Surg Educ. 2018;**75**(5):1292-8.

33. Inglis T, Dalzell K, Hooper G, et al. Does orthopedic training compromise the outcome in total hip arthroplasty? J Surg Educ. 2013;**70**(1):76-80.

34. Sayers A, Crowther MJ, Judge A, et al. Determining the sample size required to establish whether a medical device is non-inferior to an external benchmark. BMJ Open. 2017;**7**(8):e015397.

For peer review only

For peer review only - http://bmjopen.bmj.com/site/about/guidelines.xhtml

Figure and Title Legends

Figure 1 – Study flow diagram

Figure 2 – Meta-analysis of net implant survival of THRs at five years according to surgeon grade

Figure 3 – Meta-analysis of the relative risk of revision of THRs at five and ten years according to surgeon grade

Figure 4 – Meta-analysis of net implant survival of TKRs at ten years according to surgeon grade

Table 1 – Characteristics of included studies

Table 2 – GRADE Summary of Findings Table

Supplementary Table 1 – Risk of bias (ROBINS-I) assessment of methodological quality

For peer review only

Contributors

TF, AB, AS, and MW conceived and designed the study; TF and AA independently screened the articles and performed data extraction in duplicate; TF and AS were responsible for data analysis; all authors were responsible for interpreting the data; TF drafted the manuscript; AB, AA, AS, and MW revised the article critically for important intellectual content; all authors reviewed the final version of the manuscript and gave approval for submission for publication. The corresponding author attests that all listed authors meet authorship criteria and that no others meeting the criteria have been omitted.

Funding

This study was supported by the National Institute for Health Research (NIHR) Biomedical Research Centre at the University Hospitals Bristol NHS Foundation Trust and the University of Bristol (grant number: N/A). TF was supported by a NIHR Academic Clinical Fellowship. AS was supported by an MRC strategic skills fellowship (grant number: MR/L01226X/1).

Disclaimer

The views expressed in this publication are those of the authors and not necessarily those of the NHS, the NIHR, or the Department of Health and Social Care. The NIHR had no role in the design and conduct of the study; the collection, management, analysis, and interpretation of the data; the preparation, review, or approval of the manuscript; or the decision to submit the manuscript for publication.

Competing Interests

AB and MW declare support from The Healthcare Quality Improvement Partnership/The NJR in the form of the Lot 2 contract for statistical analysis of the NJR, outside the submitted work; AB and MW report grants from the NIHR investigating the outcomes of joint replacement, outside the submitted work; AB and MW are editors of an Orthopaedic textbook for which they receive royalty payments

For peer review only

For peer review only - http://bmjopen.bmj.com/site/about/guidelines.xhtml

from Taylor Francis; MW reports fees paid to their institution for delivering teaching at courses organised by DePuy and Heraeus; no other relationships or activities that could appear to have influenced the submitted work.

Patient consent for publication

Not required

Provenance and peer review

Not commissioned; externally peer reviewed

Data availability statement

All data relevant to the study are included in the article or as online supplementary material.

For peer review only

Table 1 - Characteristics of included studies

Source, Year	Country	Study period	Study design	Implant	Surgeon grade terminology (primary exposure)	Follow up (years)	Number of cases (trainee)	Implant brand (stem/cup if hip)	Sex (%female)	Mean age (SD or range)	Indication (%OA)	Supervision reported	Survival analysis	Revision rates reported	ROBINS-I overall risk of bias†
Hasegawa,[28] 2015	Japan	2006-10	PC	THR	Trainee vs. instructor	5	483 (259)	Multiple	-	61.3 (SD 11.6)	-	No	Yes	No	Serious
Jain,[27] 2018	UK	2005-12	RC	THR	Trainee vs. consultant	5, 10	1082 (348)	Corail/multiple	61.3	69.2 (21-94)	91.0	No	Yes (Add.)	Yes	Moderate
Muller,[26] 2013	Switzerland	2005-06	RC	THR	Junior vs. senior	5	130 (43)	Quadra-H /Versafit-CC	52.0	64 (SD 12.36)	86.0	No	Yes	Yes	Serious
Palan,[12] 2009	UK	1999-02	RC	THR	Trainee vs. consultant trainer	5	1501 (528)	Exeter/multiple	-	68.4 (21-94)	-	No	No	Yes	Moderate
Reidy,[13] 2016	UK	2003-04	RC	THR	Trainee vs. consultant	10	870 (286)	Multiple	60.5	69.5 (37-94)	94.8	Yes	Yes (no CIs)	Yes	Moderate
Faulkner,[15] 2017	UK	2003-04	RC	TKR	Trainee vs. consultant	5, 10	686 (236)	Multiple	-	69.9 (30-94)	93.1	No	Yes (Add.)	Yes	Moderate
Hernigou,[29] 2009	France	1990-95	RC	TKR	Young (<30) vs. senior	10, 15	250 (150)	Ceraver Hermes	69.7	73 (46-88)	-	No	Yes	No	Serious
Bottomley,[16] 2016	UK	1998-08	RC	UKR	Trainee vs. consultant	10	1084 (673)	Oxford	51.4	66.5 (SD 9.6)	100	Yes	Yes	Yes	Moderate
Alvand,[30] 2021	UK	2009-15	RC	UKR	Trainee vs. consultant	5	273 (118)	Oxford	49.5	67.8 (SD 10.1)	98.2	Yes	No	Yes	Moderate

PC, prospective cohort; RC, retrospective cohort; Add., additional data provided by author; CIs, confidence intervals; SD, standard deviation; † see supplementary table 1 for full risk of bias assessment

For peer review only - http://bmjopen.bmj.com/site/about/guidelines.xhtml

Table 2 – GRADE Summary of Findings Table

Outcomes	Follow up (years)	Trainee revision/cases†, n	Consultant revisions/cases†, n	Net survival/relative risk (95% CI)	Participants (studies), n	Quality of Evidence	Comments
THR: net implant survival	5	650	1045	NS: Trainee 97.9% (96.6 to 99.2)<br>NS: Consultant 98.1% (97.1 to 99.2)	1695 (3)[26-28]	Very low	Serious ROB, indirectness, and imprecision
	10	348	734	NS: Trainee 98.1% (95.9 to 99.1)<br>NS: Consultant 96.7% (94.7 to 97.9)	1082 (1)[27]	Low	Serious indirectness and imprecision
THR: crude revision rate	5	13/919	29/1794	RR: 0.88 (0.46 to 1.70)	2713 (3)[12, 26, 27]	Very low	Serious ROB, indirectness, and imprecision
	10	13/634	40/1318	RR: 0.68 (0.37 to 1.26)	1952 (2)[13, 27]	Low	Serious indirectness and imprecision
TKR: net implant survival	5	236	450	NS: Trainee 97.9% (95.0 to 99.2)<br>NS: Consultant 95.4% (93.0 to 97.0)	686 (1)[15]	Low	Serious imprecision
	10	386	550	NS: Trainee 96.2% (94.0 to 98.4)<br>NS: Consultant 95.1% (93.0 to 97.2)	936 (2)[15, 29]	Very low	Serious inconsistency and imprecision
	15	150	100	NS: Trainee 91.0% (85.0 to 97.0)<br>NS: Consultant 92.0% (90.0 to 94.0)	250 (1)[29]	Very low	Serious inconsistency and very serious imprecision
TKR: crude revision rate	5	5/236	20/450	RR: 0.47 (0.18 to 1.25)	686 (1)[15]	Low	Serious imprecision
	10	8/236	26/450	RR: 0.58 (0.27 to 1.27)	686 (1)[15]	Low	Serious imprecision
	15	4/150	4/100	RR: 0.67 (0.17 to 2.60)	250 (1)[29]	Very low	Serious inconsistency and very serious imprecision
UKR: net implant survival	10	673	411	NS: Trainee 93.0% (90.3 to 95.7)<br>NS: Consultant 93.9% (90.2 to 97.6)	1084 (1)[16]	Low	Serious imprecision
UKR: crude revision rate	5	1/118	4/155	RR: 0.33 (0.04 to 2.90)	273 (1)[30]	Low	Serious imprecision
	10	31/673	15/411	RR: 1.26 (0.69 to 2.31)	1084 (1)[16]	Low	Serious imprecision

For peer review only - http://bmjopen.bmj.com/site/about/guidelines.xhtml

GRADE, Grading of Recommendations Assessment, Development and Evaluation; CI, confidence interval; NS, net survival; RR, relative risk; †, number of revisions not reported for net implant survival; ROB, risk of bias

For peer review only

For peer review only - http://bmjopen.bmj.com/site/about/guidelines.xhtml

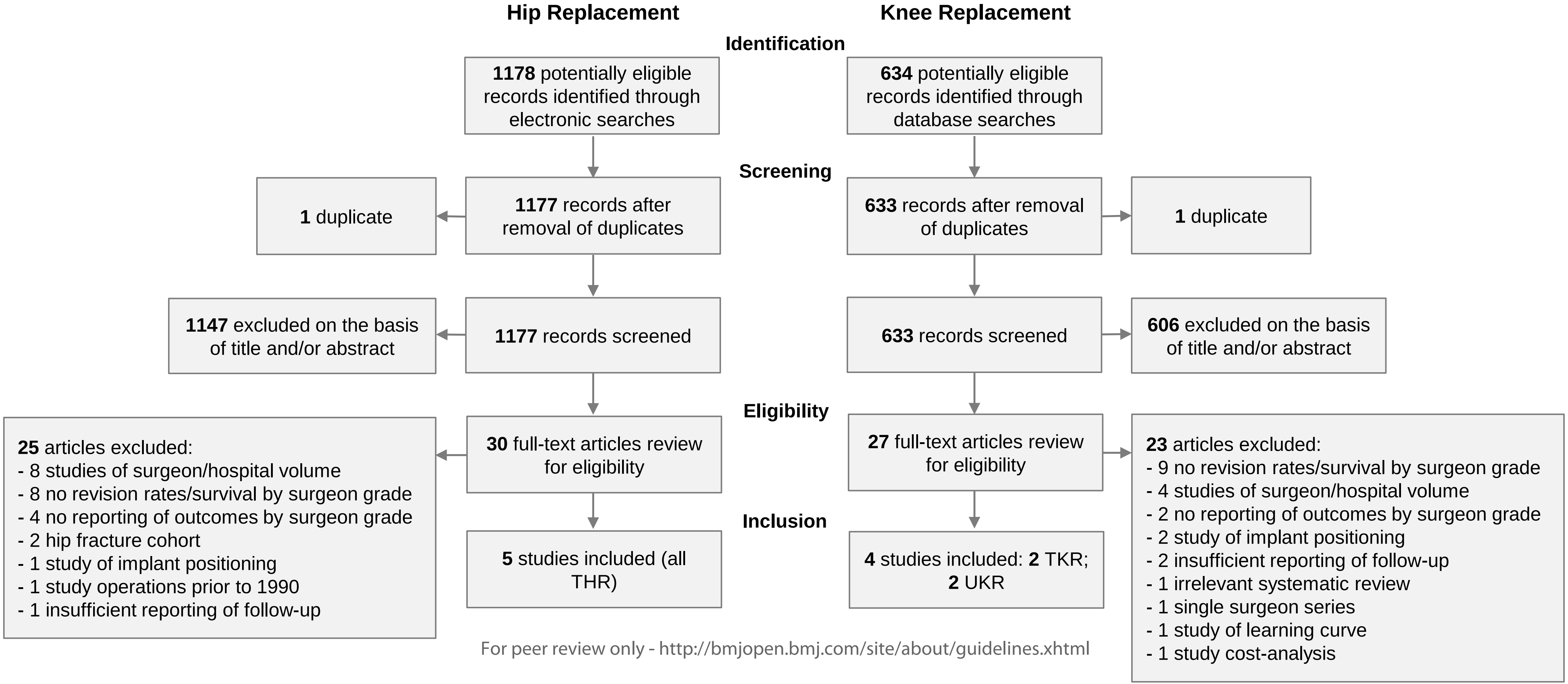

For peer review only - http://bmjopen.bmj.com/site/about/guidelines.xhtml

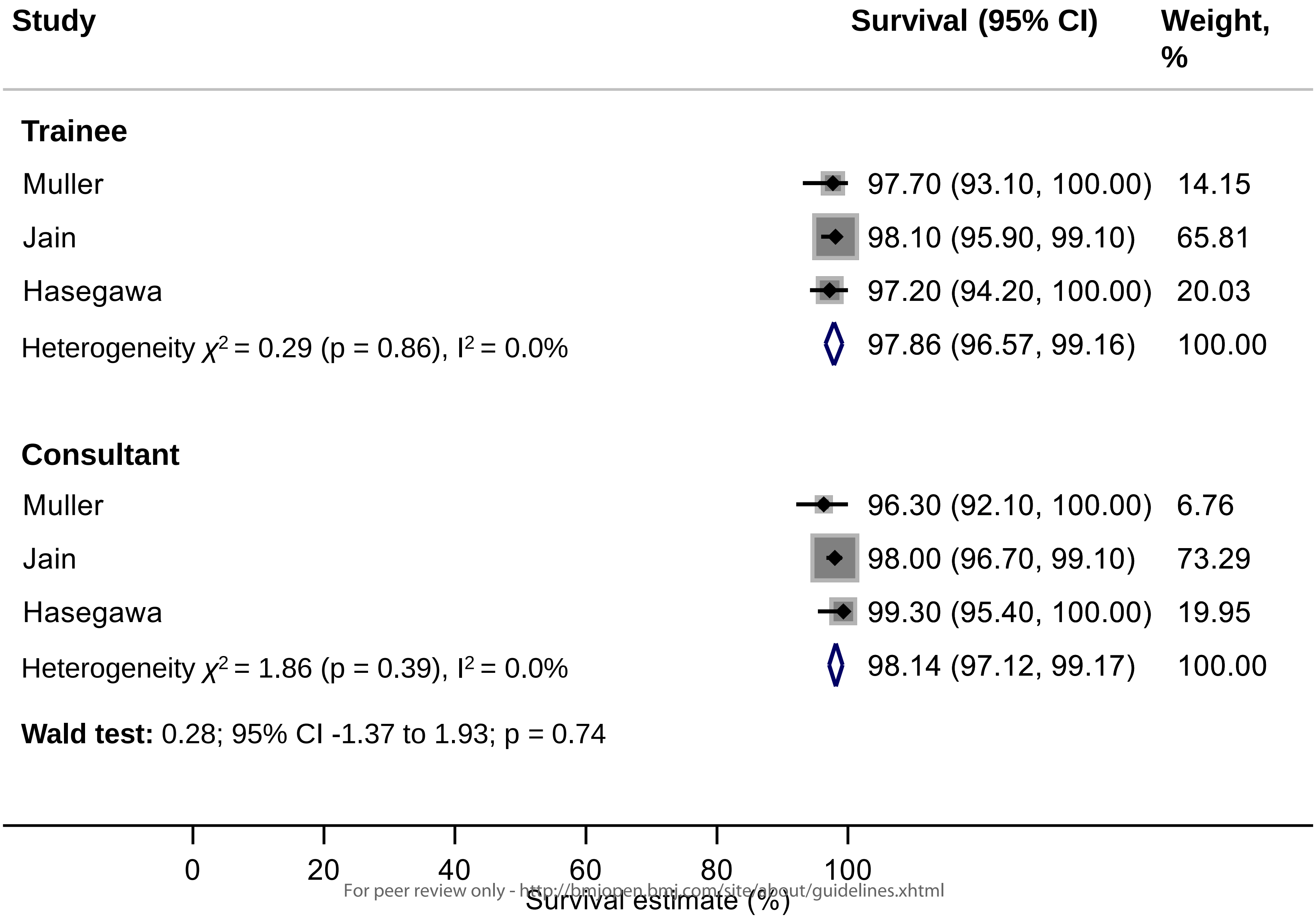
Study
Survival (95% CI)
Weight, %
Trainee
Muller
97.70 (93.10, 100.00)
14.15
Jain
98.10 (95.90, 99.10)
65.81
Hasegawa
97.20 (94.20, 100.00)
20.03
Heterogeneity χ2 = 0.29 (p = 0.86), I2 = 0.0%
97.86 (96.57, 99.16)
100.00
Consultant
Muller
96.30 (92.10, 100.00)
6.76
Jain
98.00 (96.70, 99.10)
73.29
Hasegawa
99.30 (95.40, 100.00)
19.95
Heterogeneity χ2 = 1.86 (p = 0.39), I2 = 0.0%
98.14 (97.12, 99.17)
100.00
Wald test: 0.28; 95% CI -1.37 to 1.93; p = 0.74
0
20
40
60
80
100
Survival estimate (%)

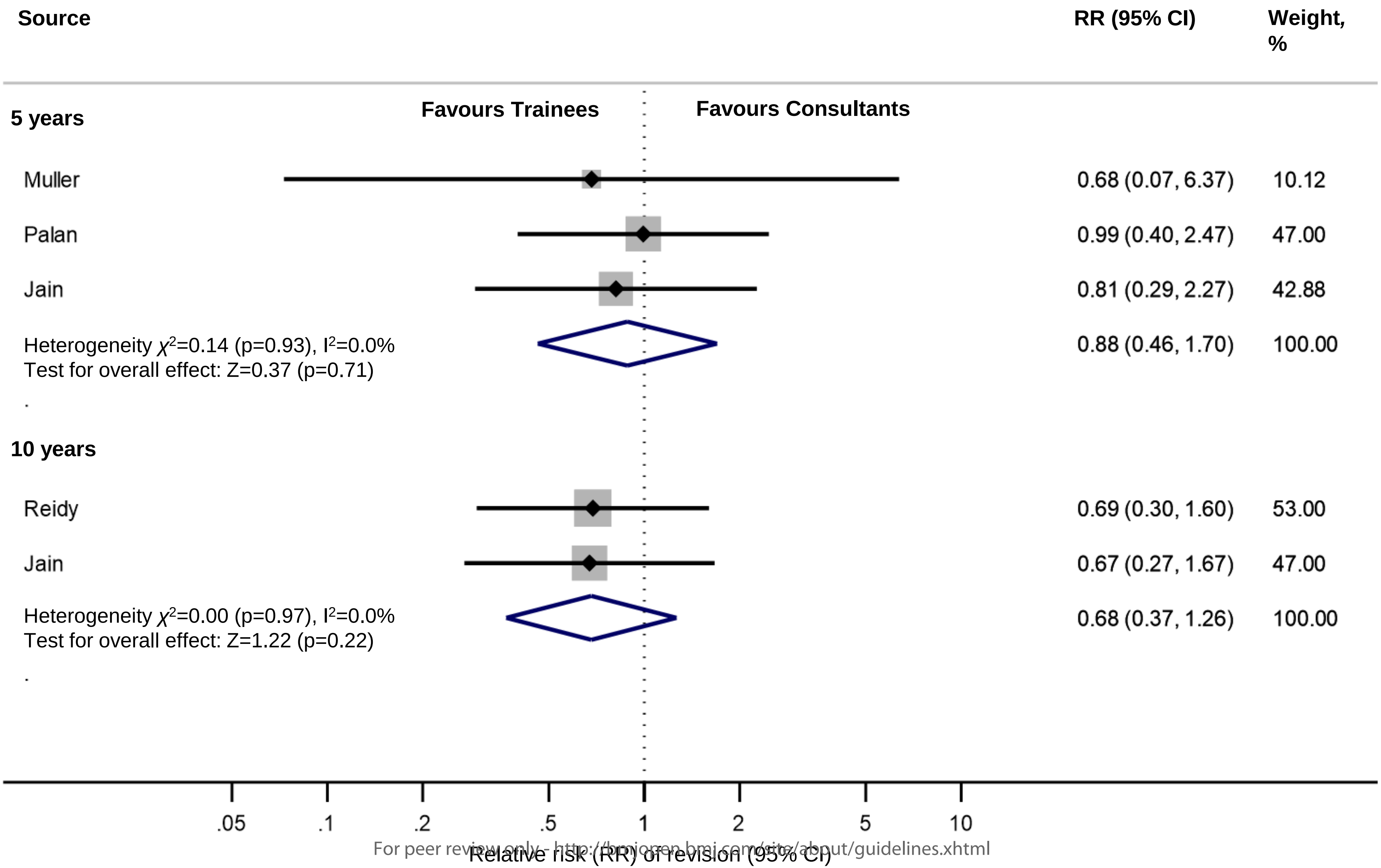

For peer review only - http://bmjopen.bmj.com/site/about/guidelines.xhtml

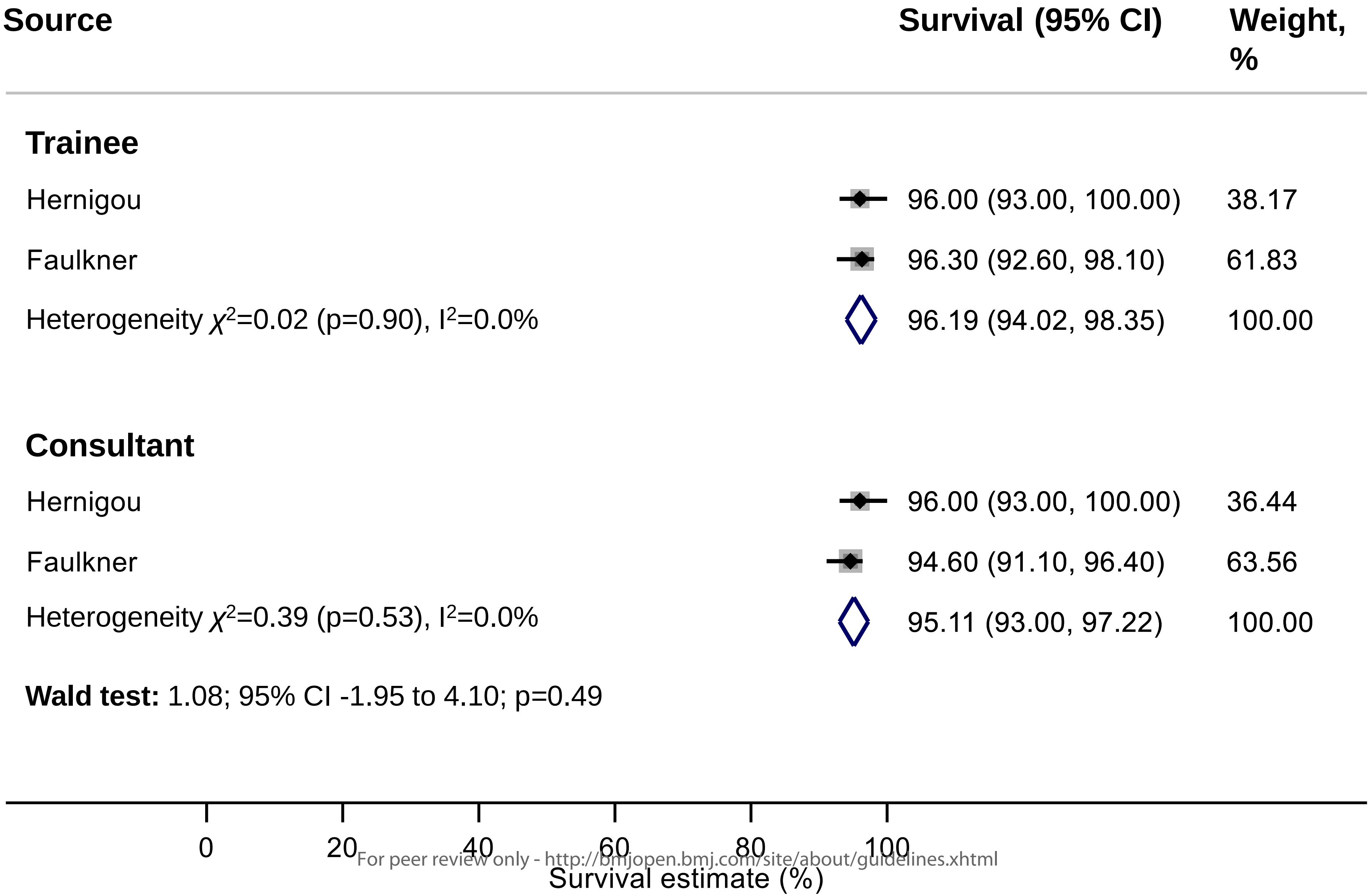

For peer review only - http://bmjopen.bmj.com/site/about/guidelines.xhtml

Online Supplementary Material

Contents:

Supplementary Methods

- Methods (page 2-3): Search strategy using Ovid (Medline + Embase)
- Methods (page 4): Eligibility criteria
- Methods (page 5): Reasons for Exclusion

Supplementary Table

- Table 3 (page 6): Risk of Bias (ROBINS-I) assessment of methodological quality

For peer review only

For peer review only - http://bmjopen.bmj.com/site/about/guidelines.xhtml

Methods: Search strategy using Ovid (Medline + Embase). Performed by TF & AS.

Hip Search

Hip replacement

Hip Prosthesis/ OR Arthroplasty, Replacement, Hip/ OR

(hip adj2 arthroplast$.mp) OR (hip adj2 replacement?.mp) OR (hip adj2 prosthes$.mp) OR

THA.mp OR THR.mp OR (TJR$.mp AND hip$.mp)

AND

Training

exp Education, Medical/ OR exp Inservice Training/ OR Clinical Competence/ OR

training.mp OR trainee.mp OR

experience.mp OR

junior.mp OR

senior$.mp OR

(surgeon adj2 grade).mp OR

consultant.mp OR attending?.mp OR registrar.mp OR SpR.mp OR StR.mp OR ST?.mp OR residen$.mp OR fellow$.mp OR intern.mp OR

(house adj2 officer).mp OR (foundation adj2 doctor).mp

AND

Survival

exp Prosthesis Failure/ OR exp Survival Analysis/ OR Reoperation/ OR

cox.mp OR proportional?hazard?.mp OR proportional hazard?.mp OR

cumulative?incidence?function.mp OR cumulative incidence function.mp OR CIF.mp OR

failure.mp OR

survival.mp OR survivor?ship.mp OR

revision?.mp OR

re?operation.mp OR re operation.mp OR

Kaplan?meier.mp OR Kaplan meier.mp OR KM.mp OR

product?limit?method.mp OR product limit method.mp

AND

Case-series

exp Cohort Studies/ OR Controlled Clinical Trials

follow?up.mp OR follow up.mp OR series.mp OR cohort.mp OR observational.mp OR longitudinal.mp OR prospective.mp OR retrospective.mp OR registry.mp OR registries.mp

For peer review only - http://bmjopen.bmj.com/site/about/guidelines.xhtml

<u>Knee search</u>

Knee replacement

Knee Prosthesis/ OR Arthroplasty, Replacement, Knee/ OR

(knee adj2 arthroplast$.mp) OR (knee adj2 replacement?.mp) OR (knee adj2 prosthes$.mp) OR

TKA.mp OR TKR.mp OR (TJR$.mp AND knee$.mp) OR

UKA.mp OR UKR.mp

AND

Training

exp Education, Medical/ OR exp Inservice Training/ OR Clinical Competence/ OR

training.mp OR trainee.mp OR

experience.mp OR

junior.mp OR

senior$.mp OR

(surgeon adj2 grade).mp OR

consultant.mp OR attending?.mp OR registrar.mp OR SpR.mp OR StR.mp OR ST?.mp OR residen$.mp OR fellow$.mp OR intern.mp OR

(house adj2 officer).mp OR (foundation adj2 doctor).mp

AND

Survival

exp Prosthesis Failure/ OR exp Survival Analysis/ OR Reoperation/ OR

cox.mp OR proportional?hazard?.mp OR proportional hazard?.mp OR

cumulative?incidence?function.mp OR cumulative incidence function.mp OR CIF.mp OR

failure.mp OR

survival.mp OR survivor?ship.mp OR

revision?.mp OR

re?operation.mp OR re operation.mp OR

Kaplan?meier.mp OR Kaplan meier.mp OR KM.mp OR

product?limit?method.mp OR product limit method.mp

AND

Case-series

exp Cohort Studies/ OR Controlled Clinical Trials

follow?up.mp OR follow up.mp OR series.mp OR cohort.mp OR observational.mp OR longitudinal.mp OR prospective.mp OR retrospective.mp OR registry.mp OR registries.mp

For peer review only - http://bmjopen.bmj.com/site/about/guidelines.xhtml

Eligibility criteria

Inclusion criteria:

- Study of predominantly adult patients (≥ 18 years old) undergoing primary hip or knee replacement (including THR, TKR, UKR and hip resurfacing), predominantly for the treatment of osteoarthritis.
- Included articles needed to report the primary and/or secondary outcome measure for two different groups of surgeons defined according to their grade (e.g. trainee vs. consultant). Additional terms used to describe this variable were deemed eligible during screening:
    - **Trainee:** registrar; resident; junior/young surgeon; fellow.
    - **Consultant:** attending; senior surgeon; trainer.
- Minimum follow-up of five years with clearly defined length of follow up.

Exclusion criteria:

- Index operation performed prior to 1990.
- Follow-up not clearly defined.
- Irrelevant study design, or outcomes (therefore not meeting inclusion criteria above).

Specific examples for exclusion (documented in online supplementary materials page 5 and figure 1):

- Principally a study of surgeon/hospital volume
- Principally a study of implant positioning
- No revision rates/survival analysis reported according to surgeon grade
- No reporting of outcomes according to surgeon grade
- Insufficient reporting of follow-up
- Study of operations performed prior to 1990
- Hip fracture cohort
- Single surgeon series
- Irrelevant systematic review
- Study of cost-analysis

For peer review only

For peer review only - http://bmjopen.bmj.com/site/about/guidelines.xhtml

Reasons for Exclusion – Hip Papers	
**First author/Year of study**	**Reason for Exclusion**
De Vries, 2011	Principally a study of surgeon/hospital volume
Fender, 2003	Principally a study of surgeon/hospital volume
Hooper, 2009	Principally a study of surgeon/hospital volume
Johnsson, 1994	Principally a study of surgeon/hospital volume
Namba, 2012	Principally a study of surgeon/hospital volume
Ravi, 2014	Principally a study of surgeon/hospital volume
Canadian Arthroplasty Soc.,	Principally a study of surgeon/hospital volume
MacBride, 2010	Principally a study of surgeon/hospital volume
Enocson, 2009	No revision rates/survival analysis reported according to surgeon grade
Field, 2006	No revision rates/survival analysis reported according to surgeon grade
Leguerrand, 2018	No revision rates/survival analysis reported according to surgeon grade
Moran, 2004	No revision rates/survival analysis reported according to surgeon grade
Smith, 2018	No revision rates/survival analysis reported according to surgeon grade
Wilson, 2016	No revision rates/survival analysis reported according to surgeon grade
Wroblewski, 1998	No revision rates/survival analysis reported according to surgeon grade
Schoenfeld, 2013	No revision rates/survival analysis reported according to surgeon grade
Inglis, 2013	Insufficient reporting of follow-up
Marston, 1996	Study of operations performed prior to 1990
Khatod, 2014	No reporting of outcomes according to surgeon grade
Whitehouse, 2014	No reporting of outcomes according to surgeon grade
Williams, 2002	No reporting of outcomes according to surgeon grade
Zwartele, 2005	No reporting of outcomes according to surgeon grade
Kim, 2017	Principally a study of implant positioning
MacDonald, 2020	Hip fracture cohort
DeAngelis, 2020	Hip fracture cohort
N.B. Multiple reasons for some papers	

Reasons for Exclusion – Knee Papers	
**First author/Year of study**	**Reason for Exclusion**
Bini, 2013	Principally a study of surgeon/hospital volume
Namba, 2012	Principally a study of surgeon/hospital volume
Zambianchi, 2014	Principally a study of surgeon/hospital volume
Rissolio, 2021	Principally a study of surgeon/hospital volume
Liddle, 2014	No revision rates/survival analysis reported according to surgeon grade
Beattie, 2016	No revision rates/survival analysis reported according to surgeon grade
Haughom, 2014	No revision rates/survival analysis reported according to surgeon grade
Khakha, 2015	No revision rates/survival analysis reported according to surgeon grade
Schoenfeld, 2013	No revision rates/survival analysis reported according to surgeon grade
Windisch, 2017	No revision rates/survival analysis reported according to surgeon grade
Wilson, 2016	No revision rates/survival analysis reported according to surgeon grade
Woolson, 2007	No revision rates/survival analysis reported according to surgeon grade
Atrey, 2014	No reporting of outcomes according to surgeon grade
Back, 2000	No reporting of outcomes according to surgeon grade
Singh, 2021	No reporting of outcomes according to surgeon grade
Gaillard, 2016	Principally a study of implant positioning
Mahaluxmivala, 2001	Principally a study of implant positioning
Storey, 2018	Insufficient reporting of follow-up
Theelen, 2018	Insufficient reporting of follow-up
Jasper, 2016	Irrelevant systematic review
Lacko, 2018	Single surgeon series
Matas-Diez, 2018	Principally a study of learning curve
Lavernia, 2000	Study of cost-analysis
N.B. Multiple reasons for some papers	

For peer review only - http://bmjopen.bmj.com/site/about/guidelines.xhtml

Supplementary Table 1: Risk of Bias (ROBINS-I) assessment

ROBINS-I	Alvand, 2021	Bottomley, 2016	Faulkner, 2017	Hernigou, 2009	Hasegawa, 2015	Jain, 2018	Muller, 2013	Palan, 2009	Reidy, 2016
Bias due to confounding	⊕⊕	⊕⊕	⊕	⊕⊕	⊕⊕	⊕	⊕⊕	⊕	⊕
Bias in selection of patients	⊕	⊕	⊕	⊕	⊕	⊕	⊕	⊕	⊕
Bias in classification of interventions	⊖	⊖	⊕	⊕	⊕⊕	⊕	⊕⊕	⊕	⊕
Bias due to deviations from interventions	⊕	⊕	⊕	⊕⊕	⊕⊕	⊕	⊕⊕	⊕	⊕
Bias due to missing data	⊖	⊖	⊕	⊕⊕	⊕⊕	⊕	⊕	⊖	⊕
Bias in measurement of outcome	⊖	⊖	⊕	⊖	⊕	⊖	⊖	⊖	⊕
Bias in selection of the reported result	⊖	⊖	⊖	⊖	⊕	⊖	⊕	⊖	⊖
Overall risk of Bias	⊕	⊕	⊕	⊕⊕	⊕⊕	⊕	⊕⊕	⊕	⊕
**Key:** ⊖ = low risk of bias; ⊕ = moderate risk of bias; ⊕⊕ = serious risk of bias; ⊕⊕⊕ = critical risk of bias									

For peer review only

MOOSE Checklist for Meta-analyses of Observational Studies

Item No	Recommendation	Reported on Page No
Reporting of background should include		
1	Problem definition	4-5
2	Hypothesis statement	4-5
3	Description of study outcome(s)	4
4	Type of exposure or intervention used	4
5	Type of study designs used	6
6	Study population	6-7
Reporting of search strategy should include		
7	Qualifications of searchers (e.g., librarians and investigators)	Online supplementary methods
8	Search strategy, including time period included in the synthesis and key words	6 & online supplementary methods
9	Effort to include all available studies, including contact with authors	7, 9
10	Databases and registries searched	7
11	Search software used, name and version, including special features used (e.g., explosion)	7-8
12	Use of hand searching (e.g., reference lists of obtained articles)	6
13	List of citations located and those excluded, including justification	Online supplementary methods
14	Method of addressing articles published in languages other than English	6
15	Method of handling abstracts and unpublished studies	6-7
16	Description of any contact with authors	7, 10-12
Reporting of methods should include		
17	Description of relevance or appropriateness of studies assembled for assessing the hypothesis to be tested	6-8
18	Rationale for the selection and coding of data (e.g., sound clinical principles or convenience)	6-8
19	Documentation of how data were classified and coded (e.g., multiple raters, blinding and interrater reliability)	7-8
20	Assessment of confounding (e.g., comparability of cases and controls in studies where appropriate)	8
21	Assessment of study quality, including blinding of quality assessors, stratification or regression on possible predictors of study results	8
22	Assessment of heterogeneity	8
23	Description of statistical methods (e.g., complete description of fixed or random effects models, justification of whether the chosen models account for predictors of study results, dose-response models, or cumulative meta-analysis) in sufficient detail to be replicated	8-9
24	Provision of appropriate tables and graphics	Table 1-2, Figures 1-4
Reporting of results should include		
25	Graphic summarizing individual study estimates and overall estimate	Figures 2-4
26	Table giving descriptive information for each study included	Table 1-2
27	Results of sensitivity testing (e.g., subgroup analysis)	N/A, justification

For peer review only - http://bmjopen.bmj.com/site/about/guidelines.xhtml

		13-14
28	Indication of statistical uncertainty of findings	10-15

Item No	Recommendation	Reported on Page No
Reporting of discussion should include		
29	Quantitative assessment of bias (e.g., publication bias)	10
30	Justification for exclusion (e.g., exclusion of non-English language citations)	13-14
31	Assessment of quality of included studies	10, Table 2 & supplementary Table 3
Reporting of conclusions should include		
32	Consideration of alternative explanations for observed results	13-15
33	Generalization of the conclusions (i.e., appropriate for the data presented and within the domain of the literature review)	13-15
34	Guidelines for future research	15
35	Disclosure of funding source	20

From: Stroup DF, Berlin JA, Morton SC, et al, for the Meta-analysis Of Observational Studies in Epidemiology (MOOSE) Group. Meta-analysis of Observational Studies in Epidemiology. A Proposal for Reporting. *JAMA*. 2000;283(15):2008-2012. doi: 10.1001/jama.283.15.2008.

For peer review only
